# Rapid Characterization of hERG Channel Kinetics II: Temperature Dependence

**DOI:** 10.1016/j.bpj.2019.07.030

**Published:** 2019-07-25

**Authors:** Chon Lok Lei, Michael Clerx, Kylie A. Beattie, Dario Melgari, Jules C. Hancox, David J. Gavaghan, Liudmila Polonchuk, Ken Wang, Gary R. Mirams

**Affiliations:** 1 Computational Biology, Department of Computer Science, University of Oxford, Oxford, United Kingdom; 2 School of Physiology, Pharmacology and Neuroscience, and Cardiovascular Research Laboratories, School of Medical Sciences, University of Bristol, Bristol, United Kingdom; 3 Pharma Research and Early Development, Innovation Center Basel, F. Hoffmann-La Roche, Basel, Switzerland; 4 Centre for Mathematical Medicine and Biology, School of Mathematical Sciences, University of Nottingham, Nottingham, United Kingdom

## Abstract

Ion channel behavior can depend strongly on temperature, with faster kinetics at physiological temperatures leading to considerable changes in currents relative to room temperature. These temperature-dependent changes in voltage-dependent ion channel kinetics (rates of opening, closing, inactivating, and recovery) are commonly represented with Q_10_ coefficients or an Eyring relationship. In this article, we assess the validity of these representations by characterizing channel kinetics at multiple temperatures. We focus on the human Ether-à-go-go-Related Gene (hERG) channel, which is important in drug safety assessment and commonly screened at room temperature so that results require extrapolation to physiological temperature. In Part I of this study, we established a reliable method for high-throughput characterization of hERG1a (Kv11.1) kinetics, using a 15-second information-rich optimized protocol. In this Part II, we use this protocol to study the temperature dependence of hERG kinetics using Chinese hamster ovary cells overexpressing hERG1a on the Nanion SyncroPatch 384PE, a 384-well automated patch-clamp platform, with temperature control. We characterize the temperature dependence of hERG gating by fitting the parameters of a mathematical model of hERG kinetics to data obtained at five distinct temperatures between 25 and 37°C and validate the models using different protocols. Our models reveal that activation is far more temperature sensitive than inactivation, and we observe that the temperature dependency of the kinetic parameters is not represented well by Q_10_ coefficients; it broadly follows a generalized, but not the standardly-used, Eyring relationship. We also demonstrate that experimental estimations of Q_10_ coefficients are protocol dependent. Our results show that a direct fit using our 15-s protocol best represents hERG kinetics at any given temperature and suggests that using the Generalized Eyring theory is preferable if no experimental data are available to derive model parameters at a given temperature.

## Significance

Ion channel currents are highly sensitive to temperature changes. Yet, because many experiments are performed more easily at room temperature, it is common to extrapolate findings to physiological temperatures using Q_10_ coefficients or Eyring rate theory. By applying short, information-rich protocols developed in Part I of this study, we identify how kinetic parameters change over temperature. We find that the commonly used Q_10_ and Eyring formulations are incapable of describing the parameters’ temperature dependence. A more generalized Eyring relationship works well, but remeasuring kinetics by refitting a model is optimal. The findings have implications for the accuracy of applications of Q_10_ coefficients in electrophysiology, and care is needed to avoid misleading extrapolations in their many scientific and industrial pharmaceutical applications.

## Introduction

**Figure 1:**
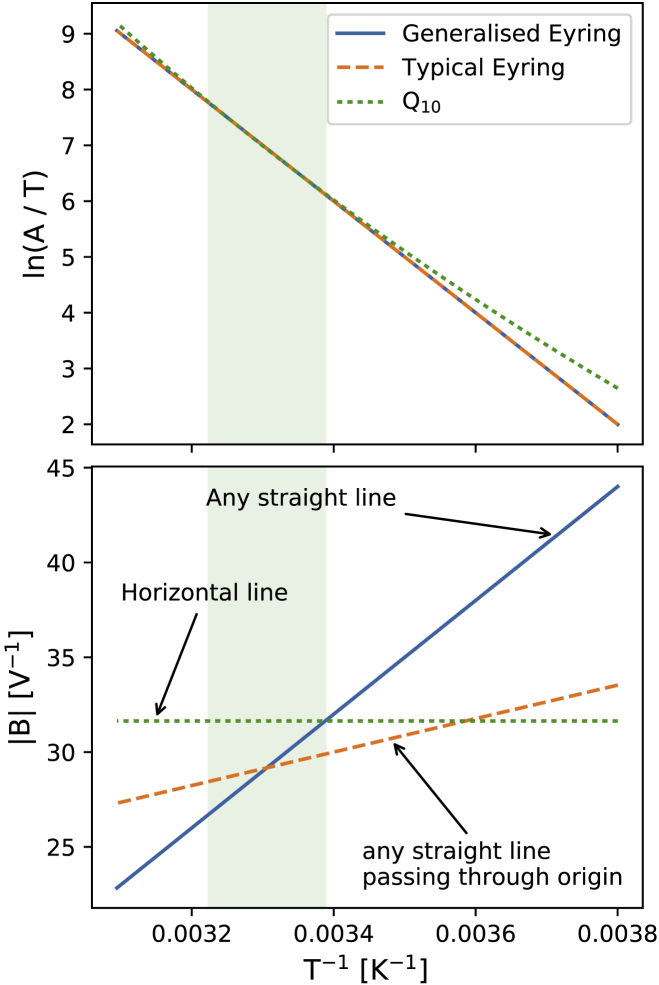
An Eyring plot illustrating the difference between a Generalized Eyring equation (Eq. 4), a Typical Eyring equation (Eq. 3), and a Q_10_ formulation (Eq. 9). This plot extends from −10 to 50°C to highlight the differences between the three formulations. The green shaded region marks the temperature range of interest, from 22 to 37°C. The Generalized Eyring relationship shown has [ln*a*_GE_, *b*_GE_, *c*_GE_, *d*_GE_] = [40, 1000, 3000, −70], and the Typical Eyring and Q_10_ relationships are the best fits to the generated Generalized Eyring relationship. Both Eyring formulations give the same straight-line dependence for ln(*A*/*T*), and even the nonlinear Q_10_ formulation is indistinguishable (for practical purposes) within the relevant temperature range. However, the three formulations can display very different behavior when examining the temperature dependence of the voltage-dependence parameter *B*. To see this figure in color, go online.

Ion channel behavior can depend strongly on temperature (1, 2), with physiological temperatures typically leading to faster kinetics and different magnitudes of current than at room temperature (see for example Fig. 1 in (3)). These temperature-dependent changes in voltage-dependent ion channel kinetics (e.g., rates of activation, deactivation, inactivation, and recovery) are commonly represented with either Q_10_ coefficients or an Eyring relationship. Here, we characterize channel kinetics at multiple temperatures and test the validity of Q_10_ and Eyring rate theories by testing whether the kinetic parameters follow the trends that these theories assume. For this case study, we use the hERG channel, which has been shown to have temperature-dependent kinetics (3, 4, 5).

The *human Ether-à-go-go-Related Gene* (*hERG*) encodes the pore-forming *α* subunit of the ion channel Kv11.1 that conducts the rapidly activating cardiac delayed rectifier potassium current (*I*_Kr_) (6). Unless otherwise specified, we refer to hERG1a simply as “hERG” in the remainder of this article. Pharmaceutical compounds that block *I*_Kr_ can prolong the cardiac ventricular action potential (7) and are associated with both increased QT intervals on the body-surface electrocardiogram and elevated risk of Torsade de Pointes arrhythmia in patients (8). The existing International Council for Harmonization S7B regulatory guidelines for pharmaceutical development require the evaluation of drug effects on the hERG channel as part of the preclinical safety testing during drug development (9).

Drug effects on hERG are typically characterized by the concentration at which *I_Kr_* conductivity is reduced by 50% (the “IC_50_”) (10). However, no single measurement temperature nor method is used consistently across different laboratories for measuring hERG IC_50_ values. Zhou et al. (4) and Vandenberg et al. (3) measured hERG1a temperature dependence and compared room and physiological temperature kinetics under typical activation and inactivation current-voltage (I-V) protocols. A similar study with hERG1a/1b was performed more recently by Mauerhöfer and Bauer (5). These studies consistently report that hERG kinetics are highly temperature sensitive, which is perhaps a property of potassium channels in general (2). The use of different temperatures and voltage protocols is therefore thought to be a large source of (deterministic) variation in IC_50_ values (11, 12, 13).

In addition, drug screening data are often collected at room temperature and requires extrapolation to physiological temperature. The temperature extrapolation relies heavily on the accuracy of models of temperature dependence. Some effort has been made to model temperature effects on hERG kinetics based upon literature data (3, 4); for example, Fink et al. (14) attempted to use an Eyring relationship and Li et al. (15) used Q_10_ coefficients. However, a detailed comparison and assessment of the applicability of these representations has not yet been undertaken.

In this article, we study and model the temperature dependence of hERG kinetics using a cell-specific fitting technique for a range of room-to-physiological temperatures. We employ a staircase protocol that is applicable in automated high-throughput patch-clamp systems, developed in Part I of this study (16). We use a mechanistic model and its parameterization to characterize hERG kinetics at multiple temperatures and compare whether these follow the temperature dependence of rate theories. Below, we discuss commonly used temperature adjustments/models for kinetic rates in voltage-gated ion channels—the Eyring relationship and the Q_10_ coefficient—and the consequences of these theories for the temperature dependence of parameters within an ion channel model.

### Models of transition rates and their temperature dependence

Mathematical ion channel models are often expressed as a Hodgkin-Huxley model (17) or a Markov state model (18), and both have rates (which we will call *k*) for transitions between the channel gates/states. To derive the rate *k* of transition between two states, the occupancy of two states—*p*(*a*) and *p*(*b*)—at equilibrium is assumed to follow a Maxwell-Boltzmann distribution:

(1) $$\frac{p\left(a\right)}{p\left(b\right)}=\text{exp}\left(-\frac{\Delta G}{RT}\right),$$

where *ΔG* is the Gibbs free energy difference between the *a* and *b* states, *R* is the ideal gas constant, and *T* is the absolute temperature. The Gibbs free energy *ΔG* is assumed to be linearly proportional to the membrane potential *V*. Assuming a simple energy barrier model, where only one rate-limiting step is required to transition between two states, the transition rate *k* is then directly proportional to the fraction of system in the excited state, which leads to the commonly used exponential form (19, 20, 21):

(2) k=Aexp(BV),

where *A* and *B* are model parameters (constants). In this study, we use the terms “parameter *A*” and “parameter *B*” to refer to *A* and *B* in Eq. 2.

#### Eyring formulations

The temperature dependence of channel transitions is embodied in the Eyring equation. The original Eyring equation was derived from basic thermodynamics and statistical mechanics, following from the the concepts of Gibbs free energy, entropy, and enthalpy (22). The typical form used to model voltage-dependent transition rates previously (14, 19, 21, 23) is as follows:

(3) $$k_{\text{TypicalEyring}}=\frac{k_{B}}{h}\cdot T\cdot \text{exp}\left(\frac{\Delta S}{R}-\frac{\Delta H}{R}\frac{1}{T}+\frac{z_{e}F}{R}\frac{1}{T}V\right),$$

with physical constants: *k*_*B*_ the Boltzmann constant, *R* the ideal gas constant, *h* the Planck constant, *F* the Faraday constant, *T* the absolute temperature, and *V* the transmembrane voltage. The following are unknowns (or “kinetic parameters”) to be determined: *ΔS* the entropy difference, *ΔH* the enthalpy difference, and *z*_*e*_ the effective valency of the structure undergoing conformational change. A more generalized Eyring relationship can be given by the following:

(4) $$k_{\text{GeneralisedEyring}}=\frac{k_{B}}{h}\cdot T\cdot \text{exp}\left(\frac{\Delta S}{R}-\frac{\Delta H}{R}\frac{1}{T}+\frac{z_{e}F}{R}\frac{1}{T}V+DV\right),$$

where *D* is a coefficient that describes a temperature-independent effect of voltage on the transition rate. The Generalized Eyring relationship is commonly used in the field of engineering (for example (24, 25, 26, 27)), although to the best of our knowledge, it has not been directly applied to ion channel modeling.

Without loss of generality, we can rewrite (reparametrize) Eq. 4, using unknowns *a*_GE_, *b*_GE_, *c*_GE_, and *d*_GE_, absorbing all other constants into these four new parameters, as follows:

(5) $$k_{\text{GeneralisedEyring}}=a_{\text{GE}}\cdot T\cdot \text{exp}\left(-b_{\text{GE}}\cdot T^{-1}\right)\cdot \text{exp}\left(\left(c_{\text{GE}}\cdot T^{-1}+d_{\text{GE}}\right)V\right),$$

where *a*_GE_ = (*k*_*B*_/*h*) exp(*ΔS*/*R*), *b*_GE_ = *ΔH*/*R*, *c*_GE_ = (*z*_*e*_*F*)/*R*, and *d*_GE_ = *D*. By comparing Eqs. 2 and 5, then we have as follows:

(6) $$A=a_{\text{GE}}\cdot T\cdot \text{exp}\left(-b_{\text{GE}}\cdot T^{-1}\right),$$

(7) $$\text{ln}\left(A/T\right)=\text{ln}\left(a_{\text{GE}}\right)-b_{\text{GE}}\cdot T^{-1},$$

(8) $$\text{and}\;B=c_{\text{GE}}\cdot T^{-1}+d_{\text{GE}}.$$

Therefore, plotting ln(*A*/*T*) against *T*^−1^ should yield a linear relationship if the Generalized Eyring relationship holds. Similarly, from Eq. 8, we see that plotting *B* against *T*^−1^ yields a linear relationship for the Generalized Eyring relationship or a proportional relationship for the Typical Eyring relationship (*d*_GE_ = 0). We refer to a plot of ln(*A*/*T*) or *B* as a function of *T*^−1^ as an “Eyring plot.”

#### Q_10_ coefficients

Another approach that is commonly used to describe temperature dependence in biological and chemical processes is the use of Q_10_ coefficients. The Q_10_ relationship is an empirical expression (28), which assumes reaction rate increases exponentially with temperature, and has been applied extensively to ion channel kinetics from Hodgkin and Huxley’s work to this day (3, 4, 5, 15, 29, 30). Using Q_10_ coefficients, we can express rates as follows:

(9) $$k_{\text{Q}10}=Q_{10}^{\left(T-T_{\text{ref}}\right)/\left(10^{\circ }\text{C}\right)}\cdot \alpha \cdot \text{exp}\left(\beta V\right).$$

Here, *α* and *β* are parameters for the rate, and *T*_ref_ is the reference temperature for the extrapolation. A *Q*_10_ coefficient is, by definition, calculated using the ratio of the rates at *T*_ref_ + 10°C and *T*_ref_. Comparing Eqs. 2 and 9, we have

(10) $$\ln\;A=a_{\text{Q}10}T+c_{\text{Q}10},$$

(11) $$\text{ln}\left(A/T\right)=\frac{a_{\text{Q}10}}{T^{-1}}+\text{ln}\left(T^{-1}\right)+c_{\text{Q}10},$$

(12) and B=β,

where *a*_Q10_ = (ln *Q*_10_)/10°C, and *c*_Q10_ = ln *α* − (*T*_ref_ ln *Q*_10_)/10°C. Therefore, if the Q_10_ formulation is accurate, then plotting ln(*A*/*T*) against *T*^−1^ should yield a nonlinear relationship, and *B* against *T*^−1^ is a horizontal line.

### A theoretical comparison of the Eyring formulation and Q_10_ coefficient

We now compare the Generalized Eyring relationship (Eq. 4), the Typical Eyring relationship (Eq. 3), and the Q_10_ expression (Eq. 9). Note that the Eyring relationships have been related to the Q_10_ expression (19, 31) to interpret the Q_10_ coefficient as the change of entropy and enthalpy. However, in this study, we treat the two formulations independently.

For parameter *A* in Eq. 2, under the Eyring plot, which we plot ln(*A*/*T*) (on the *y* axis) against 1/*T* (*x* axis), both the Generalized Eyring and Typical Eyring relationships (Eq. 7) give *y* = *mx* + *c*, which is a straight line, whereas the Q_10_ expression (Eq. 11) becomes *y* = *a*/*x* + ln(*x*) + *b*, which is not. This difference could be used to tell which theory is correct, but within our temperature regime, the Q_10_ expression on the Eyring plot gives a curve that is indistinguishable, in practical terms, from a linear Eyring relationship, as shown in the top of Fig. 1.

Therefore, the only practically measurable difference between the potential temperature relationships is in *B* parameters (which set the voltage dependence of the transition rate) in Eq. 2. The Generalized Eyring relationship implies that *B* has a linear relationship with *T*^−1^; the Typical Eyring relationship restricts *B* to be directly proportional to *T*^−1^; and under the Q_10_ coefficient formulation, *B* is a constant that does not depend on temperature. These differences are illustrated in the bottom panel of Fig. 1.

The Typical Eyring relationship is a special case of the Generalized Eyring relationship, and therefore, the Typical Eyring relationship would hold if *D* = 0 were obtained when fitting the Generalized Eyring relationship; it will become clear that this is not the case for our data. We hence compare the Generalized Eyring relationship and the Q_10_ formulation in the rest of this study.

There have been previous temperature-dependent hERG modeling studies. Fink et al. (14) expressed hERG kinetics using the Typical Eyring relationship (Eq. 3), but its parameters were derived from experimentally estimated Q_10_ values in Vandenberg et al. (3), yielding an incomplete form of the Eyring relationship based on Q_10_ values. Li et al. (15) used a Q_10_ formulation (Eq. 9) to model the temperature dependence of hERG kinetics for simplicity but did not investigate to what extent this captured temperature-dependent changes in the kinetics.

Modeling temperature effects in ion channel kinetics not only has applications in cardiac safety pharmacology, it is also commonly used in action potential modeling more generally. Many cardiac action potential models (32, 33, 34, 35) adapted the Mazhari et al. (36) hERG model, which used Q_10_ values from Zhou et al. (4) to extrapolate room temperature recordings to physiological temperature. These extrapolations cause considerable changes to rates, often exceeding changes introduced when modeling diseases or other conditions (37). Similarly, the Christé et al. (38) hERG model was based on measurements at room temperature and extrapolated to 37°C using Q_10_ values from Vandenberg et al. (3). Within action potential models, many other ion current models (such as *I*_Na_, *I*_CaL_, etc.) are also based on experiments performed at different temperatures (39), most of which are then corrected via Q_10_ extrapolations (40, 41, 42, 43, 44).

## Materials and Methods

The experimental methods, mathematical model of *I*_Kr_, and the *I*_Kr_ model parameter inference methods used in this article were identical to the methods detailed in our companion article (16). We provide only a brief outline of these methods (for details, please refer to Lei et al. (16)). Here, we focus on the methods used specifically for studying the temperature dependence of the channel.

### Experimental methods

Whole-cell patch-clamp voltage clamp experiments were performed on Chinese hamster ovary (CHO) cells stably transfected with hERG1a (Kv11.1). Measurements were performed using the Nanion SyncroPatch 384PE (Nanion Technologies, Munich, Germany), an automated high-throughput platform in which each run (or chip) is able to measure 384 wells (with one cell per well) simultaneously. The temperature of machine’s “cell hotel” was set to ∼15°C. Single hole chips with medium resistance (Nanion, #221102) were used. Solutions used in all measurements are provided in Table S1.

A total of nine voltage clamp protocols were used, including the staircase protocol (16), an activation I-V protocol, a steady-state inactivation I-V protocol, a hERG screening protocol, a delayed afterdepolarization (DAD)-like protocol, an early afterdepolarization (EAD)-like protocol, and action potential-like protocols with beating frequencies of 0.5, 1, and 2 Hz. A schematic of the experimental procedure is shown in Lei et al. (16). The whole sequence of protocols was applied to every well. Details of these protocols can be found in Lei et al. (16).

Only the staircase protocol was used in fitting (or calibrating) the mathematical model. The fitted models for each cell were then validated by comparing their predictions for the other eight protocols to the experimental recordings.

#### Temperature control

The SyncroPatch platform has a temperature control unit with software PE384TemperatureControl, which consists of a temperature controller and several temperature monitors placed around the machine compartment. The machine compartment contains all the solutions on standby and is where the measurements occurred. Because the temperature controller consists of a heater with a fan, the platform can only maintain temperatures higher than room temperature. The lowest temperature we could maintain indefinitely was 25°C, which is determined by room temperature (∼22°C) plus heat generated by the machine’s operation (∼3°C), even if the heat controller itself was set to a lower temperature.

To ensure that we recorded the temperature correctly, an external K-Type thermometer was used to ensure the temperature difference between the measuring stage, and the machine in-built temperature monitors was ≤0.5°C. Note that the temperature readouts could differ from the temperature set on the controller even after equilibrium, particularly close to room temperature, so we used the thermometer and temperature monitors’ readouts as the true temperature of the experiments. The temperatures of the five experiments were 25, 27, 30, 33, and 37°C, and the uncertainty of our temperature measurements was estimated to be ±1°C by comparing the temperature differences at various locations of the compartment. Because of the machine taking a substantial amount of time to change temperature, distinct experiments were performed at different temperatures using different cells (the same cell line but different individual cells in each well on sometimes different days).

#### Postprocessing experimental data

We performed a series of quality control checks and corrections (in postprocessing) to ensure the currents recorded represent only *I*_Kr_. Leak corrections were applied to all measurements to eliminate leak current (16). E-4031 subtraction was applied to remove any native voltage-dependent ion currents that were present in CHO cells besides the overexpressed hERG1a, usually known as endogenous currents. Cells were then selected based on partially automated quality control described in Part I of this article (16), resulting in *N*_*e*_ = 124, 91, 85, 84, and 45 cells being selected for measurements at 25, 27, 30, 33, and 37°C, respectively, and our 25°C data were examined in Part I (16). The lower yield of cells at higher temperatures was mostly due to reduced success in the cell capture step, before any recording started, and to a lesser extent, the deterioration of the patch clamp. The full analysis of which quality control criteria removed cells at the various temperatures is shown in Supporting Materials and Methods, Section S12.

#### Data visualization

Each hERG-transfected CHO cell was expected to have a different total conductance, hence giving a different magnitude for the current recording. Therefore, normalization was applied for visual comparison. Note that the validation of model predictions was performed without normalization (a conductance was fitted for each cell individually). To avoid any circular reasoning involved in normalizing based on the *g*_Kr_ parameter fit within the models (which, at this point, may or may not vary with temperature), we used an experimental maximal conductance estimate. The experimental estimate is approximated by extrapolating the negative tail current, after the first +40 to −120 mV step, back to the time the voltage step occurred (see Fig. S1). Note that this normalization method is imperfect as it relies on a particular gating process (activation gate *a* ≈ 1 at the end of the +40 mV step), which has some dependence on the kinetics we aim to compare, but the 22°C parameterization of the model (45) suggests *a* ≈ 1 is a reasonable approximation (even for lower temperatures) at this point in the protocol. However, because this method removes the conductance dependency, it has a benefit over the normalization-to-a-reference-trace method used in Lei et al. (16) by preserving the different magnitudes of currents from different temperatures.

### Mathematical model

We used the same Hodgkin and Huxley-style structure hERG model described in Lei et al. (16) and in Beattie et al. (45). In this model, the current, *I*_Kr_, is modeled with a standard Ohmic expression as follows:

(13) $$I_{\text{Kr}}=g_{\text{Kr}}\cdot a\cdot r\cdot \left(V-E_{K}\right),$$

where *g*_Kr_ is the maximal conductance, *a* is a Hodgkin and Huxley (17) activation gate, and *r* is an inactivation gate. *E*_*K*_ is the reversal potential, also known as the Nernst potential, which is not inferred but is calculated directly using the following:

(14) $$E_{K}=\frac{RT}{zF}\text{ln}\left(\frac{{\left[{\text{K}}^{+}\right]}_{\text{o}}}{{\left[{\text{K}}^{+}\right]}_{\text{i}}}\right),$$

where *R* is the ideal gas constant, *T* is the absolute temperature, *F* is the Faraday constant, and *z* is the valency of the ions (equal to 1 for K^+^). [K^+^]_o_ and [K^+^]_i_ denote the extracellular and intracellular concentrations of K^+^, respectively, which are determined by the experimental solutions, 4 and 110 mM, respectively. The two gates are governed by the following:

(15) $$\frac{\text{d}a}{\text{d}t}=\frac{a_{\infty }-a}{\tau_{a}},\;\frac{\text{d}r}{\text{d}t}=\frac{r_{\infty }-r}{\tau_{r}},$$

(16) $$a_{\infty }=\frac{k_{1}}{k_{1}+k_{2}},\;r_{\infty }=\frac{k_{4}}{k_{3}+k_{4}},$$

(17) $$\tau_{a}=\frac{1}{k_{1}+k_{2}},\;\tau_{r}=\frac{1}{k_{3}+k_{4}},$$

where

(18) $$k_{1}=p_{1}\text{exp}\left(p_{2}V\right),\;k_{3}=p_{5}\text{exp}\left(p_{6}V\right),$$

(19) $$k_{2}=p_{3}\text{exp}\left(-p_{4}V\right),\;k_{4}=p_{7}\text{exp}\left(-p_{8}V\right).$$

Therefore, our model consists of nine positive parameters ***θ*** = {*g_Kr_, p*_1_, …, *p*_8_}, each of which is to be inferred from the experimental current recordings.

Simulations were run using Myokit (46), with tolerance settings for the CVODE solver (47) set to abs_tol = 10^−8^ and rel_tol = 10^−10^. All codes and data are freely available at https://github.com/CardiacModelling/hERGRapidCharacterisation, a permanently archived version is available at https://doi.org/10.6084/m9.figshare.9677369.v1.

### Independent parameter fits at each temperature

The fitting procedure described briefly here follows exactly that laid out in Part I (16) but is repeated for each of the five temperatures.

First, we defined a transformation ϕ=ln(θ) to turn our positively constrained model parameters into unconstrained parameters. For each temperature, we specified a statistical model to relate the mathematical model and the observed experimental data:

(20) $$I_{\text{Kr}}^{\text{data}}=I_{\text{Kr}}^{\text{model}}+\epsilon ,$$

where we assumed the noise term *ε* follows a normal distribution *ε* ∼ *N*(0, *σ*^2^). Writing **y** = {*y*_*k*_} for the experimental data $\left(I_{\text{Kr}}^{\text{data}}\right)$ and **z** = {*z*_*k*_} for a simulated vector $\left(I_{\text{Kr}}^{\text{model}}\right)$, the likelihood of observing a data set **y**, given ϕ, is as follows:

(21) $$p\left(y|\phi ,\sigma \right)=\frac{1}{\sqrt{2\pi \sigma^{2}}}\text{exp}\left(-\sum_{k}\frac{{\left(z_{k}\left(\phi \right)-y_{k}\right)}^{2}}{2\sigma^{2}}\right).$$

Bayes’ theorem can then be applied to calculate the likelihood of a parameter set given experimental data as follows:

(22) $$p\left(\phi ,\sigma |y\right)=\frac{p\left(\phi \right)p\left(y|\phi ,\sigma \right)}{p\left(y\right)}\;\propto \;p\left(\phi \right)p\left(y|\phi ,\sigma \right),$$

with the prior

(23) $$\;p\left(\phi \right)∼U\left(\phi^{\text{min}},\phi^{\text{max}}\right),$$

where U(⋅) represents a uniform distribution (for details see Lei et al. (16)).

For each temperature *T*, we combined multiple experimental recordings using a hierarchical Bayesian model, as in Lei et al. (16). The full hierarchical Bayesian likelihood is given by the following:

(24) $$L\left(\mu ,\Sigma ,{\left\{\theta_{j},\sigma_{j}\right\}}_{j=1}^{N_{e}}|{\left\{y_{j}\right\}}_{j=1}^{N_{e}}\right)\propto \prod_{j=1}^{N_{e}}p\left(y_{j}|\theta_{j},\sigma_{j}\right)\;\times p\left({\left\{\theta_{j}\right\}}_{j=1}^{N_{e}}|\mu ,\Sigma \right)\;\times p\left(\mu ,\Sigma \right)\times \prod_{j=1}^{N_{e}}p\left(\sigma_{j}\right),$$

where ***μ*** and **Σ** are the hyperparameters of the hierarchical model representing the mean vector and covariance matrix from which the individual “low-level” (well-specific) parameters are drawn. ${\left\{\theta_{j},\sigma_{j}\right\}}_{j=1}^{N_{e}}$ are the set of individual “low-level” parameters for each of the *N*_*e*_ repeats of the experimental recordings ${\left\{y_{j}\right\}}_{j=1}^{N_{e}}$. The four terms in Eq. 24 correspond to 1) the likelihood of all the individual (low-level) experiments, 2) the likelihood of the hyperparameters (top-level), 3) the prior distribution of the hyperparameters, and 4) the prior distribution of *σ*_*j*_.

We assumed $\phi_{j}$ for a particular cell (experiment) *j* follows a multivariate normal distribution, namely $\phi_{j}∼N\left(\mu ,\Sigma \right)$. Two distributions include variability across wells in this hierarchical Bayesian model: they are described by samples of the mean parameter vector ***μ***, and the covariance matrix **Σ**. As described in the discussion of Lei et al. (16), if we believe the well-well variability represented by **Σ** is primarily due to different patch-clamp artifacts in each well, then the uncertainty in ***μ*** represents our uncertainty in the underlying physiology, and we therefore believe it corresponds to our uncertainty in the physiological hERG temperature response rather than our expected variability in the results of future experiments, which would require **Σ** too.

For the choice of likelihoods, prior distributions, and sampling algorithms, we used the simplified pseudo-Metropolis within Gibbs (MwG) algorithm introduced in Part I ((16); Supporting Material and Methods, Section 6). All inference and sampling were done via our open source Python package, PINTS (48); the code is provided as described above.

### Fitting Eyring and Q_10_ relationships

To investigate how well the two temperature models, the Generalized Eyring and the Q_10_ relationships, can explain the temperature dependency of hERG kinetics, we fitted the two temperature models to the inferred distribution of the mean parameter vector ***μ***(*T*) for all temperatures *T*. To do so, first, we transformed both the temperature models and ***μ***(*T*) to the Eyring plot form (see Fig. 1). Second, we modeled the marginal distribution of ***μ***(*T*) of *p*_*i*_ at each *T* in the Eyring plot using a normal distribution with mean ${\bar{\mu }}_{i}\left(T^{-1}\right)$ and standard deviation (SD) *σ*_*μ,i*_(*T*^−1^). We further assumed both ${\bar{\mu }}_{i}\left(T^{-1}\right)$ and *σ*_*μ,i*_(*T*^−1^) follow the temperature models, given by Eqs. 7 and 8 for the Generalized Eyring relationship and Eqs. 11 and 12 for the Q_10_ formulation.

Finally, given ${\bar{\mu }}_{i}\left(T^{-1}\right)$ and *σ*_*μ,i*_(*T*^−1^) (Fig. S8), we applied linear regression for parameters *A* and *B* in the Generalized Eyring model (Eqs. 7 and 8 to infer *a*_GE,_ *b*_GE_, *c*_GE_, and *d*_GE_) and a least-squares method for only parameters *A* in the Q_10_ relationship (Eq. 11 to infer *a*_Q10_ and *c*_Q10_) with the Levenberg-Marquardt algorithm provided in SciPy (49): once to fit the mean and once to fit the SD of each parameter as a function of temperature. Because of the simplicity of the problem after our transformation, a relatively simple optimization algorithm was sufficient. For the constant *B* parameter in the Q_10_ relationship (Eq. 12), we followed the standard way of using a Q_10_ relationship in which rates are extrapolated from room temperature. Therefore, we extrapolated to other temperatures using ${\bar{\mu }}_{i}\left(T^{-1}\right)$ and *σ*_*μ,i*_(*T*^−1^) at *T* = 25°C.

The estimated mean as a function of temperature was used to perform predictions for each temperature model; the estimated SD as a function of temperature allowed us to compute the uncertainty bounds for the *I*_Kr_ model parameters for each temperature model.

## Results

### Temperature dependence of recordings

**Figure 2:**
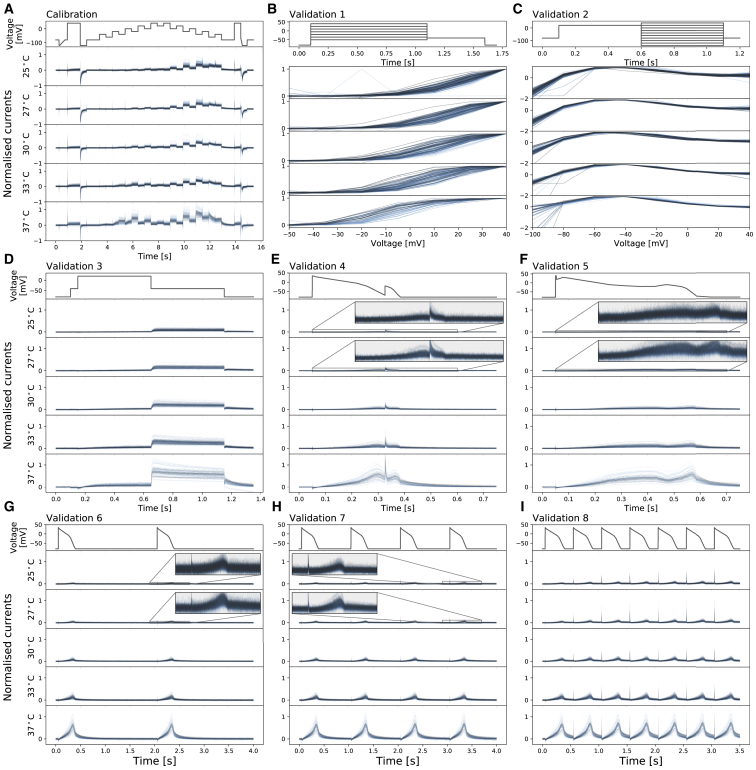
Whole-cell patch-clamp voltage clamp recordings under nine different protocols, which were all measured in each cell, at five temperatures. Each panel, from top to bottom, shows the voltage clamp protocol (*black*) and normalized current recordings (*blue*) that passed quality control at 25, 27, 30, 33, and 37°C, respectively. Currents were normalized with the method described in the text (see Fig. S1). (*A*) The calibration protocol and the staircase protocol are shown. (*B*–*I*) Shown are the eight different protocols used as validation of the model calibration, which are the activation current-voltage (I-V) protocol, the steady-state inactivation I-V protocol, the hERG screening protocol, the DAD-like protocol, the EAD-like protocol, and the cardiac action potential-like protocol at 0.5, 1, and 2 Hz, respectively. In (*B* and *C*), validation 1 and 2 show the I-V relations extracted from the currents. To see this figure in color, go online.

Fig. 2 shows the normalized voltage clamp recordings measured with the nine different protocols, and the corresponding voltage protocols, at the five temperatures. Each panel, from top to bottom, shows the voltage clamp protocol (black) then the normalized recordings (blue) that passed quality control at 25, 27, 30, 33, and 37°C, respectively. All results shown are the first of the two repeats of our recordings.

Fig. 2 *A* shows the staircase calibration protocol (in black) and the corresponding experimental recordings (in blue). The change in the recorded current as temperature increased was prominent. It increased the size of the current but also highlighted alterations to the kinetics. During the first half (3–8 s) of the staircase protocol, at low temperature, there was almost no current recorded; however, at physiological temperature, the current was almost as big as the current recorded during the second half (8–13 s) of the staircase protocol. Furthermore, the shape of the current during the second half (8–13 s) of the staircase protocol also changed as temperature increased. This demonstrates that the staircase protocol contains useful information on how kinetics change with temperature.

Fig. 2, *B*–I shows experimental recordings for the other eight validation protocols from the same cells. In validation protocol 1 (Fig. 2 *B*), we saw the activation I-V curve shifting to a lower voltage at higher temperatures. In validation protocol 3 (Fig. 2 *D*) and validation protocols 6–9 (Fig. 2, *G*–I), larger hERG currents were observed at higher temperatures. Both these responses for hERG have been reported previously (3).

### Temperature-dependent fits and predictions

In Lei et al. (16), we showed exclusively the quality of fits and predictions for the hERG models at 25°C as this could be most easily compared with previous manual patch results (45); the models replicated both the experimental training and validation data very well.

**Figure 3:**
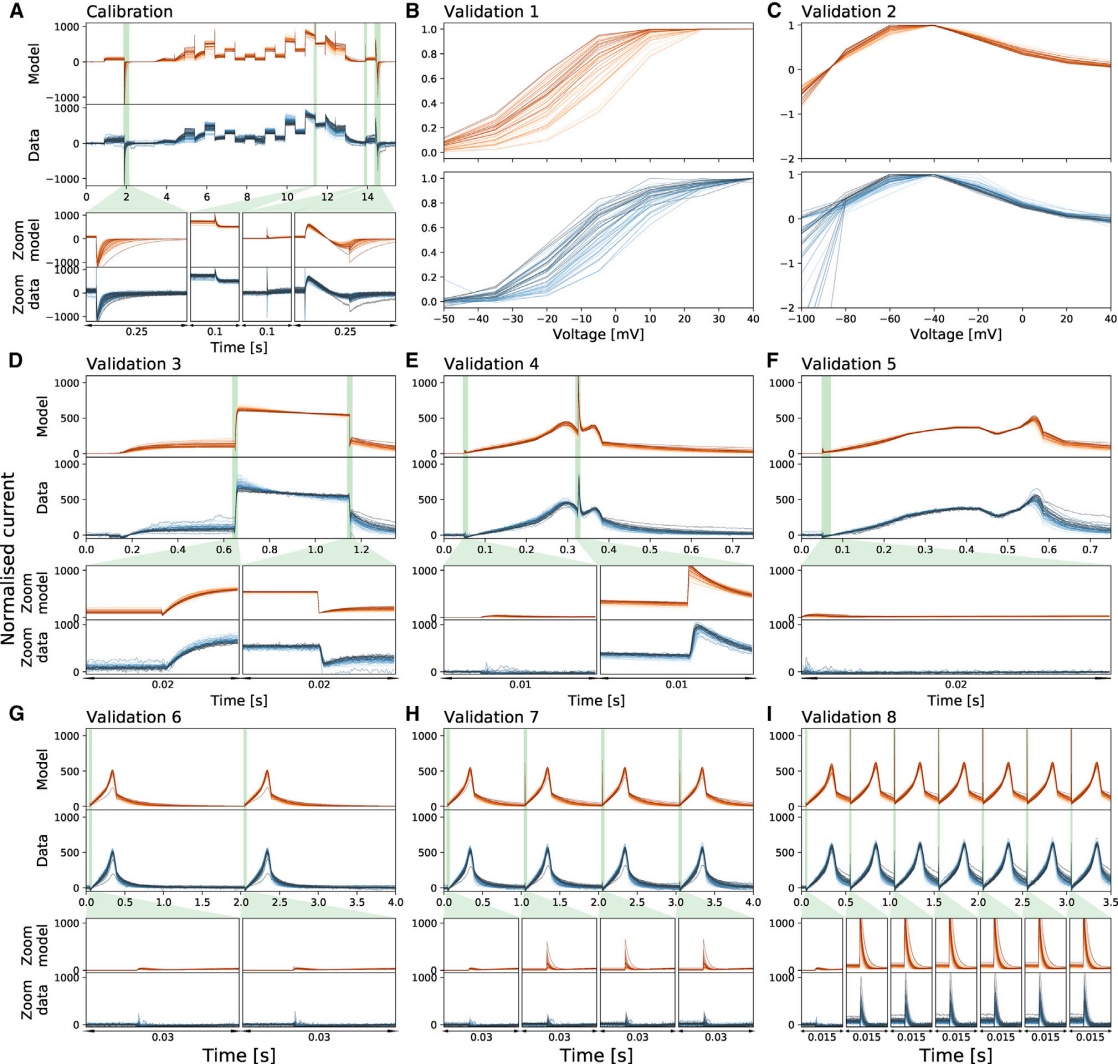
Whole-cell patch-clamp voltage clamp recordings under nine different protocols and the model fitting and validation results at 37°C. All currents are normalized by scaling them to minimize the absolute difference between each trace and a reference trace. From (*A*) to (*I*): Shown are the results of the staircase protocol, which is used as the calibration protocol, the activation current-voltage (I-V) protocol, the steady-state inactivation I-V protocol, the hERG screening protocol, the DAD-like protocol, the EAD-like protocol, and the cardiac action potential-like protocol at 0.5, 1, and 2 Hz, respectively. All the model calibration results and validation predictions are shown in the top panels (*orange*) and are compared against the experimental recordings shown in the bottom panels (*blue*). Zoomed-in of the green shaded regions are shown underneath each panel to reveal the details of the spikes, in which our models show extraordinary good predictions to the details. The normalized current for all protocols are shown except for the activation I-V protocol and the steady-state inactivation I-V protocol in which the summary statistic I-V relationships are shown. Each cell is shown with a unique color. To see this figure in color, go online.

Fig. 3 shows the model fitting and validation results for all recorded cells at 37°C alongside the experimental recordings measured under the nine different protocols. We fitted the model to the staircase protocol (Fig. 3 *A*) and validated against the other eight protocols (Fig. 3, *B*–I). To visually compare the variability in hERG kinetics (and not conductance), currents are normalized by scaling them to minimize the absolute difference between each trace and a reference trace (as in (16)). Similar plots for all the intermediate temperatures are shown in Figs. S2–S4.

We applied the same error measure as in Part I of the study to quantify the fits and predictions—the relative root mean-square error (RRMSE), defined as follows:

(25) $$\text{RRMSE}=\sqrt{\sum {\left(I_{\text{Kr}}^{\text{model}}-I_{\text{Kr}}^{\text{data}}\right)}^{2}/\sum {\left(I_{\text{Kr}}^{\text{data}}\right)}^{2}}.$$

**Figure 4:**
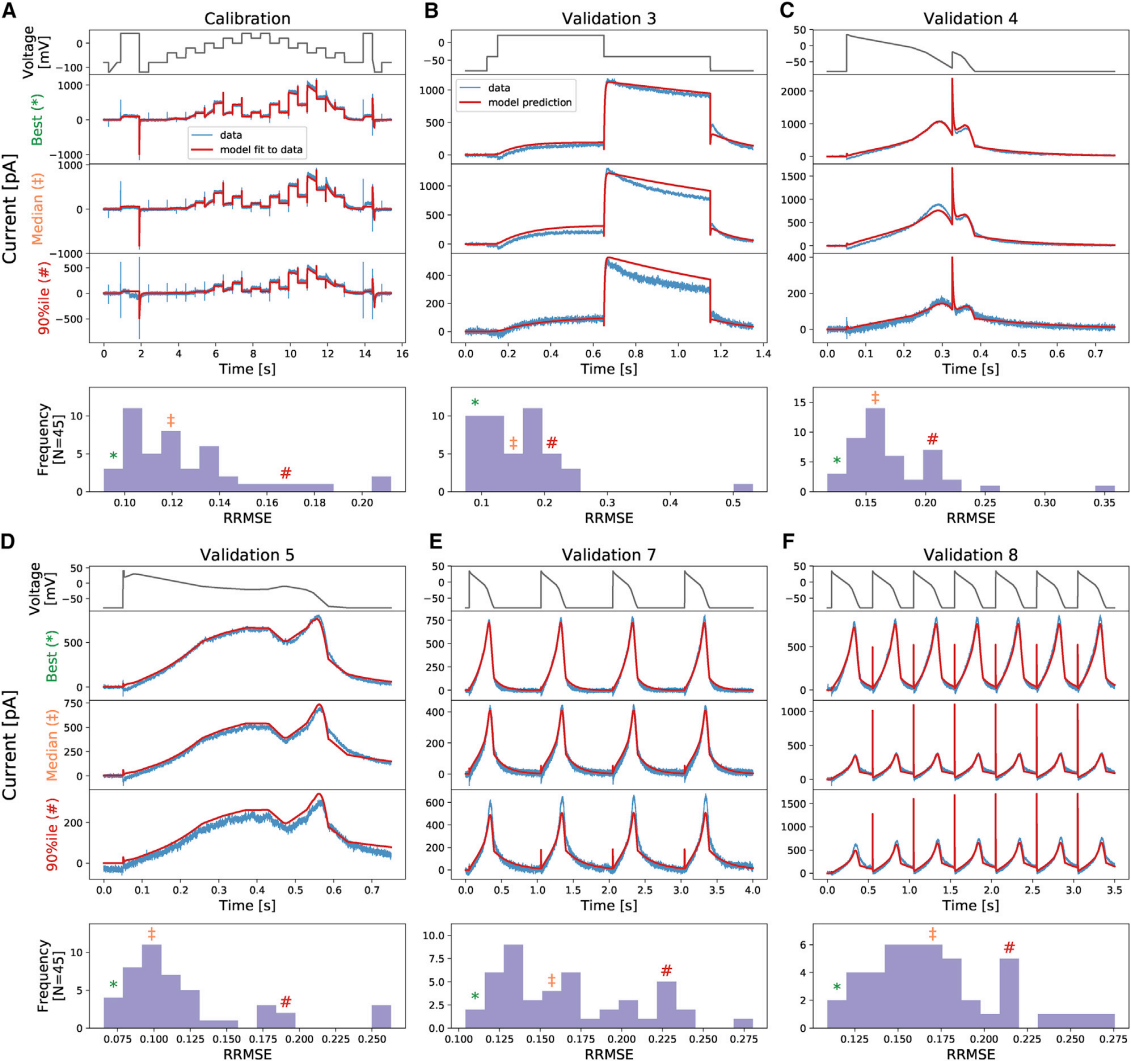
The relative root mean-square error (RRMSE, given by Eq. 25) histograms for all cells and for six protocols (*A*–*F*) at 37°C. Markers indicate the best (^∗^), median (‡), and 90th percentile (#) RRMSE values. The raw traces with the best, median, and 90th percentile RRMSE values, for both the model (*red*) and data (*blue*), are shown in the panels above, together with the voltage protocol shown on top. Note that the currents are shown on different *y* axis limits to reveal the details of the traces. The same analysis is presented for the remaining protocols in Fig. S16. To see this figure in color, go online.

Here, $I_{\text{Kr}}^{\text{model}},I_{\text{Kr}}^{\text{data}}$ are the model predictions and recordings of *I*_Kr_, respectively. Fig. 4 shows the RRMSE histograms for all cells and for the six current trace protocols at 37°C. Markers indicate the best (^∗^), median (‡), and 90th percentile (#) RRMSE values, and corresponding raw traces and predictions are shown in the three panels above. The same analysis is presented for the remaining protocols in Fig. S16. We note that the models only show single exponential decays because of the limitations of the model structure, whereas the data seem to show double exponential decays. These results demonstrate that the hERG model remains a very good representation of the current kinetics, even at 37°C, the highest temperature. The same analysis has been applied to the intermediate temperatures; the results are shown in Figs. S5–S7 and S13–S15.

### Temperature dependence of inferred model parameters

**Figure 5:**
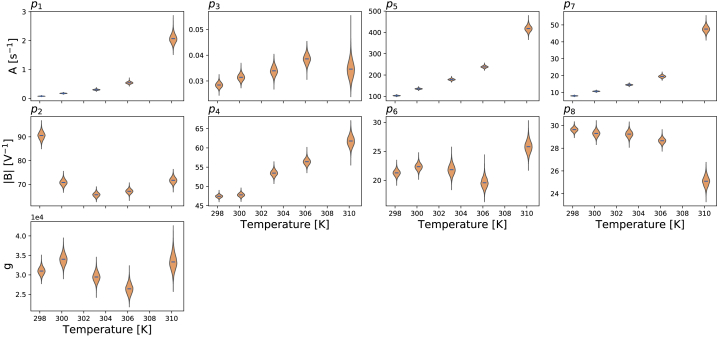
Model parameters plotted as a function of temperature. Here, only the inferred distribution of the hyperparameter mean vector ***μ*** (Eq. 24) using the simplified pseudo-MwG at each temperature is shown. Parameters *A* and *B* refer to Eq. 2. Model parameters show different degrees of temperature dependency. The conductance *g* does not show a prominent change as the temperature increases.

Fig. 5 shows the inferred parameter values, which are used in the model predictions in Figs. 3 and S2–S4, as a function of temperature. The figure shows the inferred distribution of the hyperparameter mean vector ***μ*** (Eq. 24) using the simplified pseudo-MwG at each temperature in a violin plot. The mean values and 95% credible intervals of the hyperparameter mean vector ***μ*** for all temperatures are provided in Tables S2 and S3.

If the model kinetics were exhibiting temperature dependence following Q_10_ or Eyring rate theory, then lines whose function is specified by these principles would fit the inferred parameters in Fig. 5.

In Fig. 5, most parameters show an obvious monotonic trend as temperature increases, although a handful take a slightly more complicated form. It is obvious that the *B* parameters in the second row, p_*i*_ with even *i*, are not constant over temperatures as would be expected from the Q_10_ relationship. An Eyring plot version of Fig. 5 is shown in Fig. S8. We will compare these inferred parameters with the theoretical relationships in detail in the next section.

**Figure 6:**
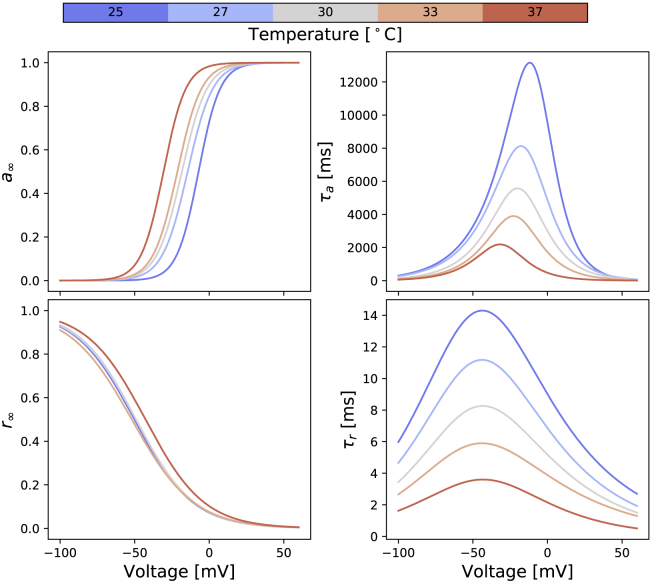
Predicted voltage dependency of steady states and time constants of the model gates *a* and *r* at different temperatures. These lines are calculated directly from inferred parameters using Eqs. 16 and 17 with the independently fitted hierarchical Bayesian model mean values. To see this figure in color, go online.

We then applied Eqs. 16 and 17 to calculate the steady states *a*_∞_ and *r*_∞_ and time constants *τ*_*a*_ and *τ*_*r*_ at the five temperatures, using the mean of the inferred distribution of ***μ*** at each temperature. Fig. 6 shows the resulting voltage dependency of the steady states and time constants of the model gates *a* and *r*, in which each temperature is indicated by a different color (25°C, blue to 37°C, red).

Fig. 6 shows that as temperature increases, the steady state of the activation gate *a* shifts in a negative voltage direction, a prediction from the fitted model that is in agreement with the experimental observations in validation protocol 1; the voltage of half-maximal activation (*V*_1/2_) of *a*_∞_ shifts from 7.5 mV at 25°C to 30.9 mV at 37°C, without a noticeable change in the slope factor. However, the steady state of the inactivation gate *r* does not show a prominent change over temperatures.

The time constant of both gates *τ*_*a*_ and *τ*_*r*_ show a similar effect as temperature increases; the maximal *τ*_*a*_ drops from 13.2 s at 25°C to 2.2 s at 37°C, and the maximal *τ*_*r*_ drops from 14.3 ms at 25°C to 3.6 ms at 37°C. Note that *τ*_*a*_ is in the order of seconds, whereas *τ*_*r*_ is in milliseconds. The voltage that maximizes the time constant shifts from 11.6 mV at 25°C to 31.7 mV at 37°C for the activation gate, although it does not show a noticeable change for the inactivation gate.

We compared the model given by the mean of the posterior for ***μ*** at 37°C (Table S2) with existing *I*_Kr_ models from within action potential models by using the Cardiac Electrophysiology Web Lab (50, 51). The CellML description (www.cellml.org (52)) is available in the Supporting Materials and Methods. Interestingly, the new model shows a striking concordance for predicted current under action potential clamps with the Markov model by Fink et al. (14); results are shown in Fig. S21.

### Comparing models of temperature dependence

**Figure 7:**
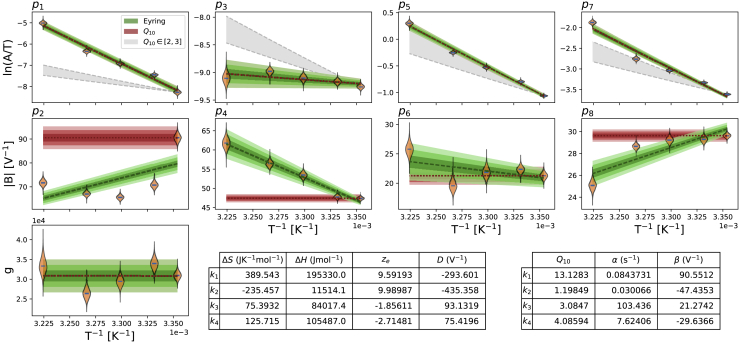
Fitting of Generalized Eyring equation and Q_10_ equation to the distribution of the mean parameter values (mean over all wells, ***μ***, shown with an *orange* violin plot) on the Eyring axes. The obtained Generalized Eyring fits are shown as green fan charts with the first three standard deviations; the obtained Q_10_ fits are shown in red. The fitted parameters for the Generalized Eyring and Q_10_ equations are shown in the bottom right tables, one set for each *k*_*i*_, with *i* = 1, 2, 3, and 4. For Q_10_ equations, *T*_ref_ = 298.15 K was used. Note that the nonzero estimations of *D* in the Generalized Eyring relationship indicate that the Typical Eyring cannot fit to all *B* parameters as it is required to go through the origin. For comparison to typical Q_10_ values in literature, in which Q_10_ values are commonly assumed to be around 2 to 3, we show a Q_10_*∈*[2, 3] relationship with the gray shaded region. To see this figure in color, go online.

Fig. 7 shows the Generalized Eyring relationship and the Q_10_ equation fitted to the inferred parameters shown in Fig. 5 (orange violin plot). The results are shown in the Eyring plot form: ln(*A*/*T*) and |B| as functions of *T*^−1^. A version of Fig. 7 with model parameters plotted directly against the temperature is shown in Fig. S9. The Generalized Eyring fits are shown as green fan charts with the first three SDs; the Q_10_ fits are shown similarly in red. The obtained parameters for the Generalized Eyring equation (Eq. 4) and the Q_10_ equation (Eq. 9) are given in the bottom right tables, one set for each rate *k*_*i*_, for *i* = 1, 2, 3, and 4. Reassuringly, the values in the tables are comparable to (the same orders of magnitude as) typical literature values for ion channel models (1, 3, 4, 14, 19, 23).

From the illustration in Fig. 1, we expect the Generalized Eyring and Q_10_ formulations to be indistinguishable for the *A* parameters, and indeed, in Fig. 7, the green fan charts (Generalized Eyring) are on top of the red fan charts (Q_10_) in the first row; both formulations are able to fit to the model's inferred *A* parameters.

Fig. 7 shows that the Generalized Eyring equations fit better to the inferred *B* parameters than the Q_10_ equations. The Generalized Eyring equations are able to fit the inferred model parameters to a large extent, except for *p*_2_, whereas the *B* parameters in the Q_10_ equations are not temperature dependent (by definition), which is contradicted by our observations.

Furthermore, it is evident that for parameters *p*_4_ and *p*_6_, the two lines cannot intercept the *y* axis close to the origin because they are decreasing rather than increasing on these plots. Parameters *p*_2_ and *p*_8_ also have nonzero estimates of *D* in the Generalized Eyring relationship, indicating that the Typical Eyring relationship cannot be fit to any of our *B* parameters. The example shown earlier in the bottom panel of Fig. 1 is based on the Generalised Eyring, Typical Eyring and Q_10_ fits for *k_4_* (*p_7_* and *p_8_*) shown in Fig. 7. The gradient of the Generalized Eyring fit is approximately twice as steep as the Typical Eyring fit would require for *p_8_*.

In the literature, Q_10_ coefficients for biological processes such as channel gating are commonly thought to take values from around 2 to 3 (53). To investigate this assumption, we projected our 25°C model parameters directly using Eq. 9 with Q_10_ *∈*[2,3], which is shown as the gray shaded region in Fig. 7. Parameter *p*_5_ in the inactivation rate (*k*_3_) gives a Q_10_ just above 3, but none of our other inferred relationships for parameter *A* is close to the range Q_10_ *∈*[2,3].

**Figure 8:**
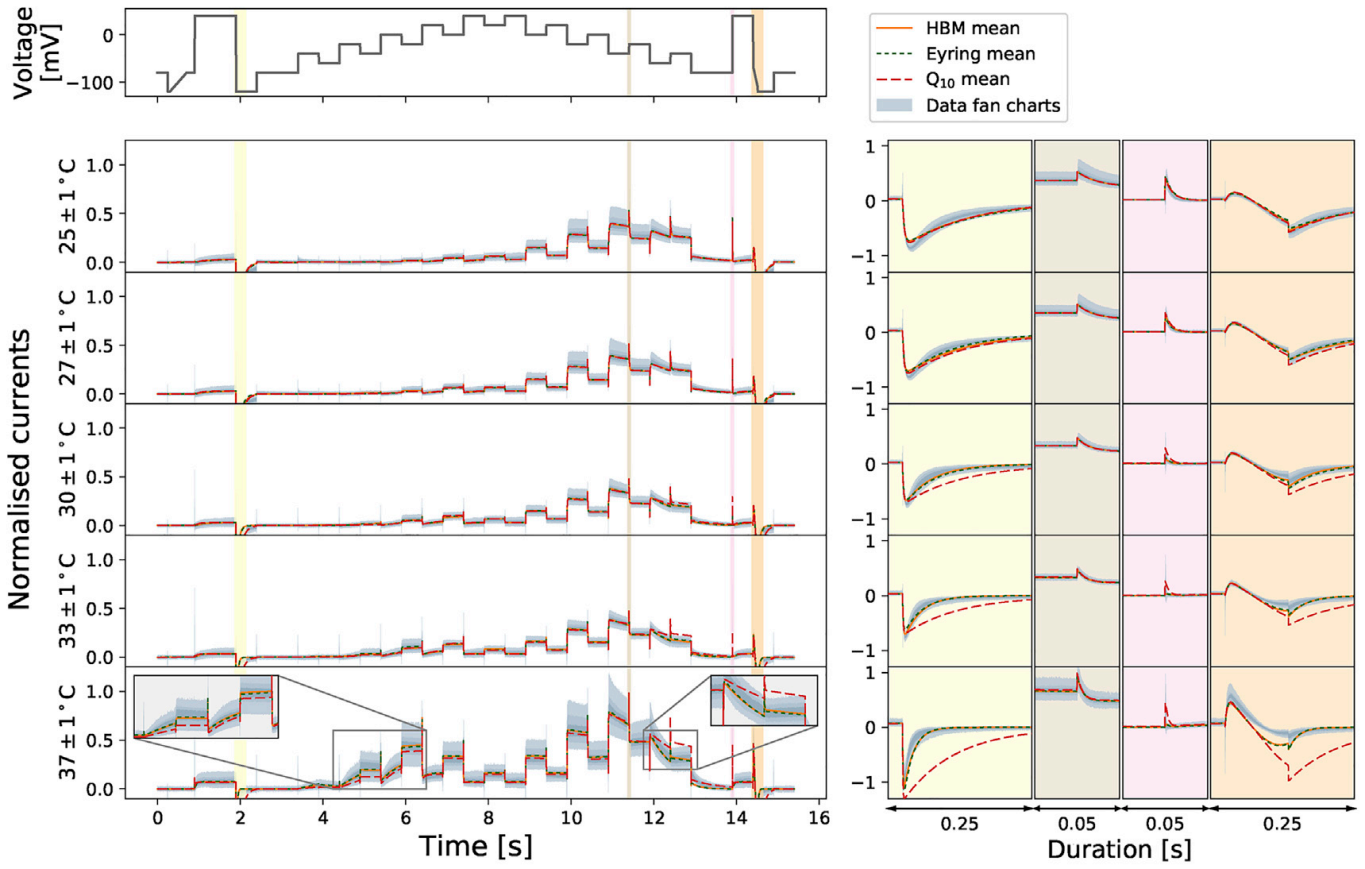
Comparison of the Generalized Eyring formulation (*dotted green*) and Q_10_ coefficient (*dashed red*) mean predictions for the staircase protocol. Top figure shows the staircase protocol, followed by the normalized current at five different temperatures. Data (in Fig. 2*A*) are shown in fan charts style with the 90^th^, 60^th^, and 30^th^ percentiles in blue. The mean prediction from the hierarchical Bayesian model (HBM) is shown in orange. Zoomed-in regions are shown on the right with colors matching the highlighted regions of the main plots on the left. To see this figure in color, go online.

We further assess the performance of the temperature dependence models by comparing their mean model predictions against the data and the temperature-specific models. Fig. 8 shows the mean model predictions from the temperature-specific parameters (orange), the Generalized Eyring formulation (dotted green), and the Q_10_ coefficient (dashed red) for the staircase protocol. All predictions are generated with the same mathematical model Eq. 13, where the rate constants in Eqs. 16 and 17 are replaced by Eq. 5 (for the Generalized Eyring formulation) and Eq. 9 (for the Q_10_ coefficient) computed with the inferred parameters shown in the tables of Fig. 7, with Q_10_-based predictions based on extrapolation from 25°C. The top panel shows the staircase protocol, followed by the normalized current at five different temperatures. Data (in Fig. 2 *A*) are shown in fan chart style with the 30^th^, 60^th^, and 90^th^ percentiles in blue. At low temperatures, all three models agree with the data. At higher temperatures, particularly at 37°C, the predictions from the Generalized Eyring formulation (dotted green) still agree reasonably with the temperature-specific independently fitted parameters (orange), and both fit the data (blue) well. However, the prediction from the Q_10_ coefficient deviates from the data during the spikes (see zoomed-in images on the right) and does not predict the time course accurately during 4–7 and 12–13 s of the staircase protocol (see insets in Fig. 8).

**Figure 9:**
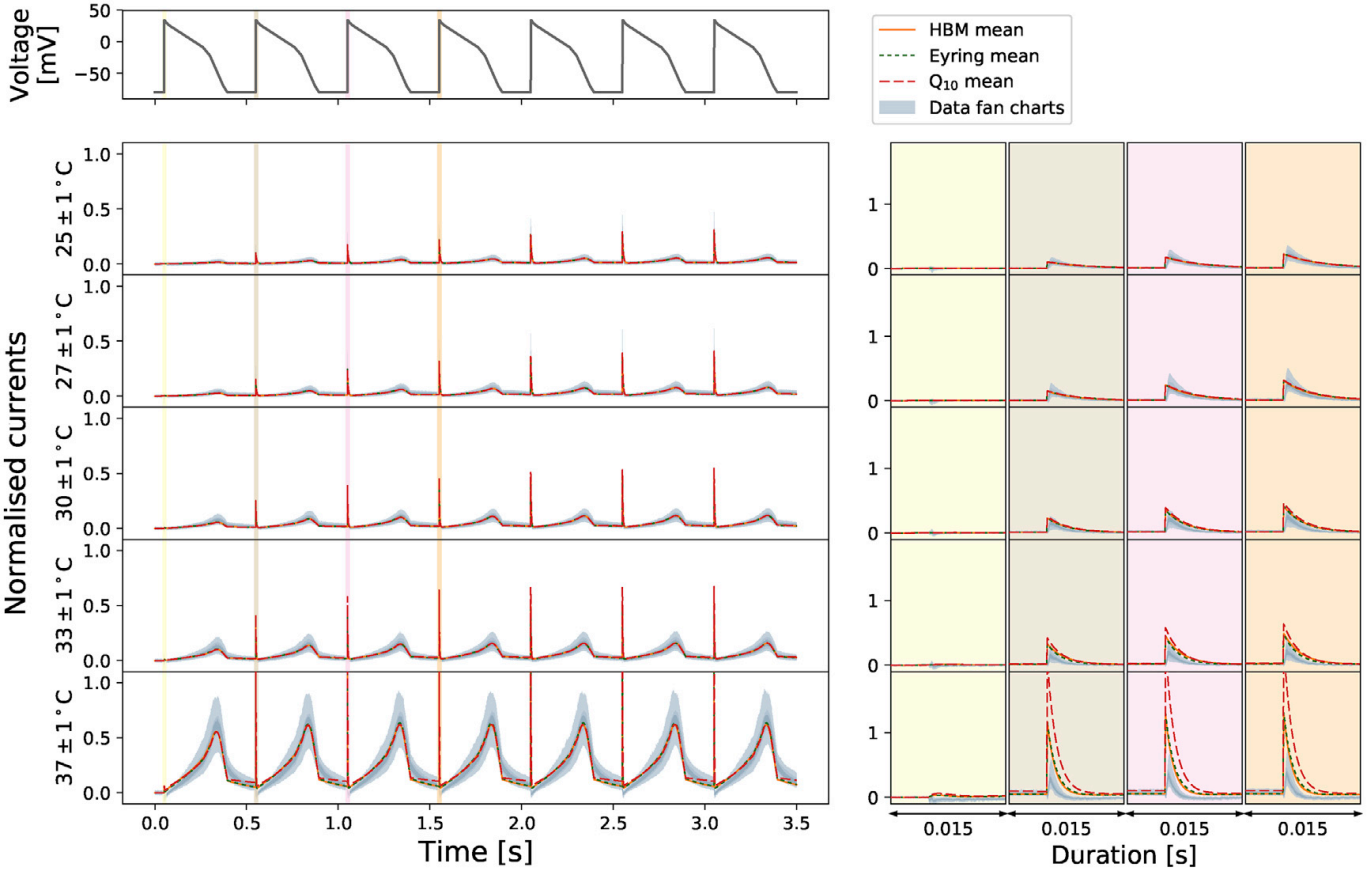
Comparison of the Generalized Eyring formulation (*dotted green*) and Q_10_ coefficient (*dashed red*) mean predictions for the 2 Hz action potential-like protocol. Top figure shows the staircase protocol, followed by the normalized current at five different temperatures. Data (Fig. 2*A*) are shown in fan charts style with the 90^th^, 60^th^, and 30^th^ percentiles in blue. The mean prediction from the hierarchical Bayesian model (HBM) is shown in orange. Zoomed-in regions are shown on the right with colors matching the highlighted regions of the main plots on the left. To see this figure in color, go online.

Fig. 9 shows a 2 Hz action potential-like protocol prediction version of Fig. 8. All the three mean models are able to predict the current during the repolarization of the action potential clamp very well. The spikes during the upstrokes are, however, badly predicted by the Q_10_ coefficient mean model, whereas the Generalized Eyring formulation, similar to the temperature-specific parameters, gives a prediction closer to the data.

## Discussion

In this study, we have examined the temperature dependence of hERG kinetics, at five temperatures ranging from room to body temperature, with 45–124 cells per temperature. We have used a mechanistic model and its parameterization to capture our knowledge of the hERG kinetics. By assuming that all cells share the same mechanism underlying hERG kinetics, we have based our study on the inferred model parameters at different temperatures to reveal the temperature dependence of hERG gating kinetics. This is, to our knowledge, the first systematic effort to have taken this approach.

Using the staircase protocol, we were able to characterize hERG kinetics to the extent that our model can replicate both the experimental training and validation data very well for all of the measured temperatures. Our models can predict the current response to the physiologically relevant action potential protocols with a very high accuracy, demonstrating that our *I*_Kr_ models are robust in predicting hERG current, in both healthy and arrhythmic situations. This gives us confidence that the cell-specific model parameters do represent and capture hERG kinetics at the given temperatures.

The directly fitted models reveal that the activation gate has a much higher temperature sensitivity than the inactivation gate. This effect is shown in both the comparison of steady states and time constants (Fig. 6) and the inferred Q_10_ coefficients (Fig. 7) in which the Q_10_ values for the activation gate (*k*_1_ and *k*_2_) are overall higher than the inactivation gate (*k*_3_ and *k*_4_). Our inferred Q_10_ coefficient for the rate of activation (*k*_1_) is relatively high compared to literature results (3, 4). However, our findings are not implausible when compared to other potassium channels, such as Kv2.1 and Kv4.3, which can have maximal Q_10_ values up to the 20–30 range (2). Other ion channels can also exhibit a very high temperature sensitivity, such as transient receptor potential ion channels, which were reported to have Q_10_ values ranging from 2 to 15 in Dhaka et al. (1). We then further compare our model predictions with the literature results in Vandenberg et al. (3).

**Figure 10:**
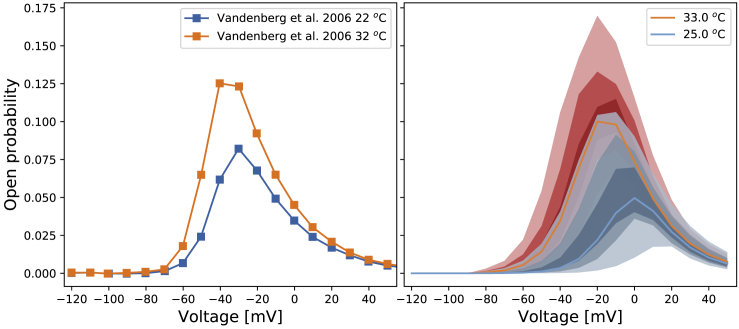
Voltage dependence of steady-state “open probability” as defined in Vandenberg et al. ((3), Fig. 6) using a multiplication of experimental approximations for the product *a*_∞_*r*_∞_. Left: Data were extracted from Vandenberg et al. ((3), Fig. 6). Right: The fan charts show the 90th, 60th, and 30th percentiles of the hierarchical Bayesian model simulations, representing the experiment-experiment variability. Orange/red represents 32 or 33°C, and blue represents 22 or 25°C in the respective studies. To see this figure in color, go online.

Our hierarchical Bayesian models at different temperatures are not only able to predict our validation data but also able to reproduce the temperature dependence seen in previous studies (3), in which the increase of temperature caused a large increase in the overall “steady-state open probability.” In Supporting Materials and Methods, Section S8, we describe how we reproduced Fig. 6 of Vandenberg et al. (3). Fig. 10 shows that our simulations (right panel) are broadly consistent with the temperature effect observed in Vandenberg et al. (3) (left panel). The fan charts show the 30th, 60th, and 90th percentiles of the simulations, representing the inter-experiment (well-well) variability. There are differences between our simulations and their experimental results, with a smaller open probability at low temperatures in our simulations and a slight shift of the curves to the right. Nevertheless, our results are broadly consistent with the temperature effect observed in Vandenberg et al. (3) and predict a very similar “width” for this steady-state window of open probability and also agree with the absolute values of the probabilities at the higher temperature very well.

Q_10_ formulations have often been estimated in the past with different protocols, even for the same gating process (e.g., activation). For example, two well-known experimental studies of temperature dependence of hERG kinetics, by Zhou et al. (4) and Vandenberg et al. (3), estimated the Q_10_ coefficients using different protocols and analyses and reported two different sets of Q_10_ coefficients (see Table 1) for various gating processes. We asked the following question: if the two experiments were to be repeated with the same underlying kinetics, would they agree with one another? Using our directly fitted models at 25 and 37°C, we simulated the two different sets of experiments described in (3, 4) (for details, see Supporting Materials and Methods, Section S9). We then estimated two sets of Q_10_ coefficients following the protocols and analysis in each of the articles, and the obtained values are shown in Table 1. The findings in Table 1 show strong evidence that because of different protocols, the estimated Q_10_ coefficients can disagree. Furthermore, neither of the protocols reproduces the direct estimate of Q_10_ coefficients from the model parameter temperature relationships (shown in the bottom right of Fig. 7). We conclude that extreme caution should be used when directly modifying rates in models with experimental estimations of Q_10_ coefficients.

**Table 1:** The Protocol Dependence of Q_10_ Coefficient Estimates for Each Gating Process

	Zhou et al. (4)		Vandenberg et al. (3)	
	Reported Values	Model Estimation	Reported Values	Model Estimation
Activation	6.25 ± 2.55	10.668 ± 7.482	2.1 ± 0.30	7.400 ± 4.111
Deactivation	–	2.016 ± 0.764	17 ± 0.30	3.692 ± 1.224
Inactivation	3.55 ± 0.87	3.421 ± 1.028	2.5 ± 0.53	2.750 ± 0.900
Recovery	3.65 ± 0.73	2.991 ± 0.730	2.6 ± 0.26	4.436 ± 2.763

The model estimates were derived from simulated currents using the same temperature-specific parameters (from fits at 25 and 37°C) under the different protocols performed in the two literature studies.

Fitting directly to the staircase protocol at different temperatures does not require any assumption about the underlying temperature dependence of the kinetic parameters, except that the model structure does not change. The existing well-known models/approximations for temperature dependence of ion channel transition rates are the Q_10_ and Typical Eyring formulations. Our study has raised concerns about how accurate these relationships are. In terms of parameter values (Fig. 7), neither of these methods is able to capture the full temperature dependence of the directly fitted parameters, ***μ***(*T*), and predictably, this impairs their ability to fit and predict currents (Fig. 8). However, using a Generalized Eyring relationship (not commonly used in ion channel modeling) can closely mimic our full direct fitting approach (Figs. 8 and 9). Although the model predictions using the Q_10_ formulation can generally predict overall trends in temperature effects, the predictions cannot capture the details of the current compared to the Generalized Eyring relationship or the full direct fitting approach (Figs. 8 and 9). We therefore suggest neither Q_10_ formulations nor the Typical Eyring relationship should be used; the Generalized Eyring relationship is much better for temperature predictions. But for the best results, the model should be refitted at any temperature of interest using an information-rich protocol, such as our staircase protocol (16).

The nonlinearity of some kinetic parameters on the Eyring plots implies the Generalized Eyring relationship is a reasonable but imperfect temperature model. Under the assumption that the model structure is correct, we accurately captured the kinetics at each temperature, and the model structure stays the same for all temperatures. However, we could challenge these assumptions and suppose that the Generalized, or even Typical, Eyring relationship is true for any transition of ion channel from one conformational state to another. In this case, the Eyring formulation not matching the individual temperature parameter sets could imply that, either 1) the hERG model structure that we have assumed is incorrect (i.e., the relationship not holding is a consequence of discrepancy between the model and reality); 2) our procedure did not accurately capture the kinetic parameters at each temperature, but the fact that the parameters give excellent fits and predictions (and many parameters do follow expected trends) perhaps alleviates this concern; or 3) in reality, the energy landscape of ion channel conformations changes with the temperature, and a given transition in the model represents a different jump in conformational state (i.e., the model structure should change with temperature, which has been modeled previously (54)).

In any case, applying a simple treatment such as the Q_10_ coefficient to an imperfect model that violates the assumptions above would not automatically alleviate any mismatch. Because our temperature-specific fits can replicate both the experimental training data and the validation data very well at all temperatures, the model is a good representation of hERG kinetics. Hence, it is better to apply a rapid and reproducible procedure, as illustrated here, for generating all the parameters within a model at a new temperature, whenever possible. However, if necessary, then the Generalized Eyring relationship would be a preferable choice for predicting kinetics at a new temperature in which measurements cannot be, or have not been, taken. Although further work might show our results are more generally applicable to other channels, for now, they should be interpreted as being specific to hERG1a.

Our results have strong implications for how drug screening assays should be performed and interpreted. Because many of the drug screening platforms work only at an ambient temperature, measurements at different temperatures not only give rise to a large source of (deterministic) variation but also introduce the problem of translation of their findings to physiological temperatures. This translation is particularly problematic when an imperfect temperature model is used, such as the commonly used Q_10_ coefficient, as shown in this study. Extreme caution should be taken when using temperature-extrapolated in vitro drug screening data in in silico models for risk prediction.

Given Q_10_ coefficients cannot capture the full temperature dependence of hERG kinetics (as shown in Fig. 7) and different drugs target different kinetics, then a previous finding that there are no common sets of Q_10_ coefficients to describe the kinetics of drug block (55) is consistent with our results. In future, one could use our models to study some of the temperature effects observed in drug studies (11).

## Conclusions

We have studied the temperature dependence of hERG kinetics using a 15-second high-information content protocol developed in Part I of this study (16). We characterized the temperature dependence by fitting a mathematical model of hERG channel kinetics to data obtained at five distinct temperatures between 25 and 37°C. We constructed between 45 and 124 cell-specific hERG models at each temperature using the 15-second calibration protocol, and our cell-specific variants of the hERG model were able to predict currents under eight independent validation protocols with high accuracy. We represented the variability in parameters using a hierarchical Bayesian model and were able to reproduce the temperature dependence observed in previous literature studies. Our models reveal that the hERG activation process has a higher temperature sensitivity than the inactivation process. The temperature dependence of the kinetic parameters we obtained takes a more complicated form than that predicted by Q_10_ coefficients or a Typical Eyring approach, although it broadly follows a Generalized Eyring relationship. Our results show that a direct fit to the 15-second protocol is the best representation of hERG kinetics at a given temperature, although predictions from the Generalized Eyring theory may be preferentially used if no such data are available.

## Author Contributions

C.L.L., M.C., K.A.B., D.J.G., L.P., K.W., and G.R.M. designed the research. C.L.L., D.M., J.C.H., K.A.B., L.P., and K.W. carried out pilot studies and the experiments shown here. C.L.L., M.C., D.J.G., and G.R.M. designed the computational analysis. C.L.L. wrote simulation codes, performed the analysis, and generated the results figures. All authors wrote and approved the final version of the manuscript.

## Supporting Citations

Reference (56) appears in the Supporting Material

## Supporting Material

Document S1. Supporting Materials and Methods, Figs. S1–S21, Tables S1–S3

Document S2. Article plus Supporting Material
