# Supplementary material for: Rapid Characterization of hERG Channel Kinetics II: Temperature Dependence
Source: Biophys J. 2019 Jul 25;117(12):2455–70. doi: 10.1016/j.bpj.2019.07.030 (PMC6990152; doi:10.1016/j.bpj.2019.07.030)
Supplement: Document S2. Article plus Supporting Material [file mmc2.pdf]

Biophysical Journal
Article

Rapid Characterization of hERG Channel Kinetics II: Temperature Dependence

Chon Lok Lei,[1] Michael Clerx,[1] Kylie A. Beattie,[1] Dario Melgari,[2] Jules C. Hancox,[2] David J. Gavaghan,[1] Liudmila Polonchuk,[3] Ken Wang,[3] and Gary R. Mirams[4,*]

[1]Computational Biology, Department of Computer Science, University of Oxford, Oxford, United Kingdom; [2]School of Physiology, Pharmacology and Neuroscience, and Cardiovascular Research Laboratories, School of Medical Sciences, University of Bristol, Bristol, United Kingdom; [3]Pharma Research and Early Development, Innovation Center Basel, F. Hoffmann-La Roche, Basel, Switzerland; and [4]Centre for Mathematical Medicine and Biology, School of Mathematical Sciences, University of Nottingham, Nottingham, United Kingdom

ABSTRACT Ion channel behavior can depend strongly on temperature, with faster kinetics at physiological temperatures leading to considerable changes in currents relative to room temperature. These temperature-dependent changes in voltage-dependent ion channel kinetics (rates of opening, closing, inactivating, and recovery) are commonly represented with Q_{10} coefficients or an Eyring relationship. In this article, we assess the validity of these representations by characterizing channel kinetics at multiple temperatures. We focus on the human Ether-à-go-go-Related Gene (hERG) channel, which is important in drug safety assessment and commonly screened at room temperature so that results require extrapolation to physiological temperature. In Part I of this study, we established a reliable method for high-throughput characterization of hERG1a (Kv11.1) kinetics, using a 15-second information-rich optimized protocol. In this Part II, we use this protocol to study the temperature dependence of hERG kinetics using Chinese hamster ovary cells overexpressing hERG1a on the Nanion SyncroPatch 384PE, a 384-well automated patch-clamp platform, with temperature control. We characterize the temperature dependence of hERG gating by fitting the parameters of a mathematical model of hERG kinetics to data obtained at five distinct temperatures between 25 and 37°C and validate the models using different protocols. Our models reveal that activation is far more temperature sensitive than inactivation, and we observe that the temperature dependency of the kinetic parameters is not represented well by Q_{10} coefficients; it broadly follows a generalized, but not the standardly-used, Eyring relationship. We also demonstrate that experimental estimations of Q_{10} coefficients are protocol dependent. Our results show that a direct fit using our 15-s protocol best represents hERG kinetics at any given temperature and suggests that using the Generalized Eyring theory is preferable if no experimental data are available to derive model parameters at a given temperature.

SIGNIFICANCE Ion channel currents are highly sensitive to temperature changes. Yet, because many experiments are performed more easily at room temperature, it is common to extrapolate findings to physiological temperatures using Q_{10} coefficients or Eyring rate theory. By applying short, information-rich protocols developed in Part I of this study, we identify how kinetic parameters change over temperature. We find that the commonly used Q_{10} and Eyring formulations are incapable of describing the parameters' temperature dependence. A more generalized Eyring relationship works well, but remeasuring kinetics by refitting a model is optimal. The findings have implications for the accuracy of applications of Q_{10} coefficients in electrophysiology, and care is needed to avoid misleading extrapolations in their many scientific and industrial pharmaceutical applications.

INTRODUCTION

Ion channel behavior can depend strongly on temperature (1,2), with physiological temperatures typically leading to faster kinetics and different magnitudes of current than at room temperature (see for example Fig. 1 in (3)). These temperature-dependent changes in voltage-dependent ion channel kinetics (e.g., rates of activation, deactivation, inactivation, and recovery) are commonly represented with either Q_{10} coefficients or an Eyring relationship. Here, we characterize channel kinetics at multiple temperatures and test the validity of Q_{10} and Eyring rate theories by testing whether the kinetic parameters follow the trends that these theories assume. For this case study, we use the hERG

Submitted April 15, 2019, and accepted for publication July 17, 2019.

*Correspondence: gary.mirams@nottingham.ac.uk

Editor: Zhilin Qu.

https://doi.org/10.1016/j.bpj.2019.07.030

© 2019 Biophysical Society.

This is an open access article under the CC BY license (http://creativecommons.org/licenses/by/4.0/).

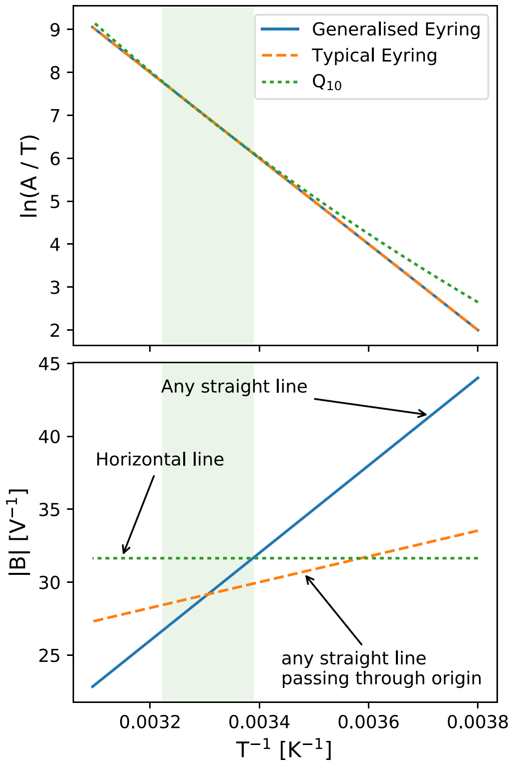

FIGURE 1 An Eyring plot illustrating the difference between a Generalized Eyring equation (Eq. 4), a Typical Eyring equation (Eq. 3), and a Q_{10} formulation (Eq. 9). This plot extends from -10 to 50°C to highlight the differences between the three formulations. The green shaded region marks the temperature range of interest, from 22 to 37°C. The Generalized Eyring relationship shown has [$\ln a_{GE}$, b_{GE}, c_{GE}, d_{GE}] = [40, 1000, 3000, -70], and the Typical Eyring and Q_{10} relationships are the best fits to the generated Generalized Eyring relationship. Both Eyring formulations give the same straight-line dependence for ln(A/T), and even the nonlinear Q_{10} formulation is indistinguishable (for practical purposes) within the relevant temperature range. However, the three formulations can display very different behavior when examining the temperature dependence of the voltage-dependence parameter B. To see this figure in color, go online.

channel, which has been shown to have temperature-dependent kinetics (3–5).

The *human Ether-à-go-go-Related Gene* (*hERG*) encodes the pore-forming α subunit of the ion channel Kv11.1 that conducts the rapidly activating cardiac delayed rectifier potassium current (I_{Kr}) (6). Unless otherwise specified, we refer to hERG1a simply as "hERG" in the remainder of this article. Pharmaceutical compounds that block I_{Kr} can prolong the cardiac ventricular action potential (7) and are associated with both increased QT intervals on the body-surface electrocardiogram and elevated risk of Torsade de Pointes arrhythmia in patients (8). The existing International Council for Harmonization S7B regulatory guidelines for pharmaceutical development require the evaluation of drug effects on the hERG channel as part of the preclinical safety testing during drug development (9).

Drug effects on hERG are typically characterized by the concentration at which I_{Kr} conductivity is reduced by 50% (the "IC_{50}") (10). However, no single measurement temperature nor method is used consistently across different laboratories for measuring hERG IC_{50} values. Zhou et al. (4) and Vandenberg et al. (3) measured hERG1a temperature dependence and compared room and physiological temperature kinetics under typical activation and inactivation current-voltage (I-V) protocols. A similar study with hERG1a/1b was performed more recently by Mauerhöfer and Bauer (5). These studies consistently report that hERG kinetics are highly temperature sensitive, which is perhaps a property of potassium channels in general (2). The use of different temperatures and voltage protocols is therefore thought to be a large source of (deterministic) variation in IC_{50} values (11–13).

In addition, drug screening data are often collected at room temperature and requires extrapolation to physiological temperature. The temperature extrapolation relies heavily on the accuracy of models of temperature dependence. Some effort has been made to model temperature effects on hERG kinetics based upon literature data (3,4); for example, Fink et al. (14) attempted to use an Eyring relationship and Li et al. (15) used Q_{10} coefficients. However, a detailed comparison and assessment of the applicability of these representations has not yet been undertaken.

In this article, we study and model the temperature dependence of hERG kinetics using a cell-specific fitting technique for a range of room-to-physiological temperatures. We employ a staircase protocol that is applicable in automated high-throughput patch-clamp systems, developed in Part I of this study (16). We use a mechanistic model and its parameterization to characterize hERG kinetics at multiple temperatures and compare whether these follow the temperature dependence of rate theories. Below, we discuss commonly used temperature adjustments/models for kinetic rates in voltage-gated ion channels—the Eyring relationship and the Q_{10} coefficient—and the consequences of these theories for the temperature dependence of parameters within an ion channel model.

Models of transition rates and their temperature dependence

Mathematical ion channel models are often expressed as a Hodgkin-Huxley model (17) or a Markov state model (18), and both have rates (which we will call k) for transitions between the channel gates/states. To derive the rate k of transition between two states, the occupancy of two states—$p(a)$ and $p(b)$—at equilibrium is assumed to follow a Maxwell-Boltzmann distribution:

$$\frac{p(a)}{p(b)} = \exp\left(-\frac{\Delta G}{RT}\right), \qquad (1)$$

where ΔG is the Gibbs free energy difference between the a and b states, R is the ideal gas constant, and T is the absolute temperature. The Gibbs free energy ΔG is assumed to be linearly proportional to the membrane potential V. Assuming a simple energy barrier model, where only one rate-limiting step is required to transition between two states, the transition rate k is then directly proportional to the fraction of system in the excited state, which leads to the commonly used exponential form (19–21):

$$k = A\exp(BV), \tag{2}$$

where A and B are model parameters (constants). In this study, we use the terms "parameter A" and "parameter B" to refer to A and B in Eq. 2.

Eyring formulations

The temperature dependence of channel transitions is embodied in the Eyring equation. The original Eyring equation was derived from basic thermodynamics and statistical mechanics, following from the the concepts of Gibbs free energy, entropy, and enthalpy (22). The typical form used to model voltage-dependent transition rates previously (14,19,21,23) is as follows:

$$k_{\text{TypicalEyring}} = \frac{k_B}{h}\cdot T\cdot\exp\left(\frac{\Delta S}{R} - \frac{\Delta H}{R}\frac{1}{T} + \frac{z_e F}{R}\frac{1}{T}V\right), \tag{3}$$

with physical constants: k_B the Boltzmann constant, R the ideal gas constant, h the Planck constant, F the Faraday constant, T the absolute temperature, and V the transmembrane voltage. The following are unknowns (or "kinetic parameters") to be determined: ΔS the entropy difference, ΔH the enthalpy difference, and z_e the effective valency of the structure undergoing conformational change. A more generalized Eyring relationship can be given by the following:

$$k_{\text{GeneralisedEyring}} = \frac{k_B}{h}\cdot T\cdot\exp\left(\frac{\Delta S}{R} - \frac{\Delta H}{R}\frac{1}{T} + \frac{z_e F}{R}\frac{1}{T}V + DV\right), \tag{4}$$

where D is a coefficient that describes a temperature-independent effect of voltage on the transition rate. The Generalized Eyring relationship is commonly used in the field of engineering (for example (24–27)), although to the best of our knowledge, it has not been directly applied to ion channel modeling.

Without loss of generality, we can rewrite (reparametrize) Eq. 4, using unknowns a_{GE}, b_{GE}, c_{GE}, and d_{GE}, absorbing all other constants into these four new parameters, as follows:

$$k_{\text{GeneralisedEyring}} = a_{\text{GE}}\cdot T\cdot\exp\left(-b_{\text{GE}}\cdot T^{-1}\right)\cdot \exp\left(\left(c_{\text{GE}}\cdot T^{-1} + d_{\text{GE}}\right)V\right), \tag{5}$$

where $a_{\text{GE}} = (k_B/h)\exp(\Delta S/R)$, $b_{\text{GE}} = \Delta H/R$, $c_{\text{GE}} = (z_e F)/R$, and $d_{\text{GE}} = D$. By comparing Eqs. 2 and 5, then we have as follows:

$$A = a_{\text{GE}}\cdot T\cdot\exp\left(-b_{\text{GE}}\cdot T^{-1}\right), \tag{6}$$

$$\ln(A/T) = \ln(a_{\text{GE}}) - b_{\text{GE}}\cdot T^{-1}, \tag{7}$$

$$\text{and}\quad B = c_{\text{GE}}\cdot T^{-1} + d_{\text{GE}}. \tag{8}$$

Therefore, plotting $\ln(A/T)$ against T^{-1} should yield a linear relationship if the Generalized Eyring relationship holds. Similarly, from Eq. 8, we see that plotting B against T^{-1} yields a linear relationship for the Generalized Eyring relationship or a proportional relationship for the Typical Eyring relationship ($d_{\text{GE}} = 0$). We refer to a plot of $\ln(A/T)$ or B as a function of T^{-1} as an "Eyring plot."

Q_{10} coefficients

Another approach that is commonly used to describe temperature dependence in biological and chemical processes is the use of Q_{10} coefficients. The Q_{10} relationship is an empirical expression (28), which assumes reaction rate increases exponentially with temperature, and has been applied extensively to ion channel kinetics from Hodgkin and Huxley's work to this day (3–5,15,29,30). Using Q_{10} coefficients, we can express rates as follows:

$$k_{\text{Q10}} = Q_{10}^{\left(T-T_{\text{ref}}\right)/\left(10^{\circ}\text{C}\right)}\cdot\alpha\cdot\exp(\beta V). \tag{9}$$

Here, α and β are parameters for the rate, and T_{ref} is the reference temperature for the extrapolation. A Q_{10} coefficient is, by definition, calculated using the ratio of the rates at $T_{\text{ref}} + 10^{\circ}$C and T_{ref}. Comparing Eqs. 2 and 9, we have

$$\ln A = a_{\text{Q10}}T + c_{\text{Q10}}, \tag{10}$$

$$\ln(A/T) = \frac{a_{\text{Q10}}}{T^{-1}} + \ln\left(T^{-1}\right) + c_{\text{Q10}}, \tag{11}$$

$$\text{and } B = \beta, \tag{12}$$

where $a_{\text{Q10}} = (\ln Q_{10})/10^{\circ}$C, and $c_{\text{Q10}} = \ln\alpha - (T_{\text{ref}}\ln Q_{10})/10^{\circ}$C. Therefore, if the Q_{10} formulation is accurate, then plotting $\ln(A/T)$ against T^{-1} should yield a nonlinear relationship, and B against T^{-1} is a horizontal line.

A theoretical comparison of the Eyring formulation and Q_{10} coefficient

We now compare the Generalized Eyring relationship (Eq. 4), the Typical Eyring relationship (Eq. 3), and the Q_{10} expression (Eq. 9). Note that the Eyring relationships

have been related to the Q_{10} expression (19,31) to interpret the Q_{10} coefficient as the change of entropy and enthalpy. However, in this study, we treat the two formulations independently.

For parameter A in Eq. 2, under the Eyring plot, which we plot $\ln(A/T)$ (on the y axis) against $1/T$ (x axis), both the Generalized Eyring and Typical Eyring relationships (Eq. 7) give $y = mx + c$, which is a straight line, whereas the Q_{10} expression (Eq. 11) becomes $y = a/x + \ln(x) + b$, which is not. This difference could be used to tell which theory is correct, but within our temperature regime, the Q_{10} expression on the Eyring plot gives a curve that is indistinguishable, in practical terms, from a linear Eyring relationship, as shown in the top of Fig. 1.

Therefore, the only practically measurable difference between the potential temperature relationships is in B parameters (which set the voltage dependence of the transition rate) in Eq. 2. The Generalized Eyring relationship implies that B has a linear relationship with T^{-1}; the Typical Eyring relationship restricts B to be directly proportional to T^{-1}; and under the Q_{10} coefficient formulation, B is a constant that does not depend on temperature. These differences are illustrated in the bottom panel of Fig. 1.

The Typical Eyring relationship is a special case of the Generalized Eyring relationship, and therefore, the Typical Eyring relationship would hold if $D = 0$ were obtained when fitting the Generalized Eyring relationship; it will become clear that this is not the case for our data. We hence compare the Generalized Eyring relationship and the Q_{10} formulation in the rest of this study.

There have been previous temperature-dependent hERG modeling studies. Fink et al. (14) expressed hERG kinetics using the Typical Eyring relationship (Eq. 3), but its parameters were derived from experimentally estimated Q_{10} values in Vandenberg et al. (3), yielding an incomplete form of the Eyring relationship based on Q_{10} values. Li et al. (15) used a Q_{10} formulation (Eq. 9) to model the temperature dependence of hERG kinetics for simplicity but did not investigate to what extent this captured temperature-dependent changes in the kinetics.

Modeling temperature effects in ion channel kinetics not only has applications in cardiac safety pharmacology, it is also commonly used in action potential modeling more generally. Many cardiac action potential models (32–35) adapted the Mazhari et al. (36) hERG model, which used Q_{10} values from Zhou et al. (4) to extrapolate room temperature recordings to physiological temperature. These extrapolations cause considerable changes to rates, often exceeding changes introduced when modeling diseases or other conditions (37). Similarly, the Christé et al. (38) hERG model was based on measurements at room temperature and extrapolated to 37°C using Q_{10} values from Vandenberg et al. (3). Within action potential models, many other ion current models (such as I_{Na}, I_{CaL}, etc.) are also based on experiments performed at different temperatures (39), most of which are then corrected via Q_{10} extrapolations (40–44).

MATERIALS AND METHODS

The experimental methods, mathematical model of I_{Kr}, and the I_{Kr} model parameter inference methods used in this article were identical to the methods detailed in our companion article (16). We provide only a brief outline of these methods (for details, please refer to Lei et al. (16)). Here, we focus on the methods used specifically for studying the temperature dependence of the channel.

Experimental methods

Whole-cell patch-clamp voltage clamp experiments were performed on Chinese hamster ovary (CHO) cells stably transfected with hERG1a (Kv11.1). Measurements were performed using the Nanion SyncroPatch 384PE (Nanion Technologies, Munich, Germany), an automated high-throughput platform in which each run (or chip) is able to measure 384 wells (with one cell per well) simultaneously. The temperature of machine's "cell hotel" was set to ~15°C. Single hole chips with medium resistance (Nanion, #221102) were used. Solutions used in all measurements are provided in Table S1.

A total of nine voltage clamp protocols were used, including the staircase protocol (16), an activation I-V protocol, a steady-state inactivation I-V protocol, a hERG screening protocol, a delayed afterdepolarization (DAD)-like protocol, an early afterdepolarization (EAD)-like protocol, and action potential-like protocols with beating frequencies of 0.5, 1, and 2 Hz. A schematic of the experimental procedure is shown in Lei et al. (16). The whole sequence of protocols was applied to every well. Details of these protocols can be found in Lei et al. (16).

Only the staircase protocol was used in fitting (or calibrating) the mathematical model. The fitted models for each cell were then validated by comparing their predictions for the other eight protocols to the experimental recordings.

Temperature control

The SyncroPatch platform has a temperature control unit with software PE384TemperatureControl, which consists of a temperature controller and several temperature monitors placed around the machine compartment. The machine compartment contains all the solutions on standby and is where the measurements occurred. Because the temperature controller consists of a heater with a fan, the platform can only maintain temperatures higher than room temperature. The lowest temperature we could maintain indefinitely was 25°C, which is determined by room temperature (~22°C) plus heat generated by the machine's operation (~3°C), even if the heat controller itself was set to a lower temperature.

To ensure that we recorded the temperature correctly, an external K-Type thermometer was used to ensure the temperature difference between the measuring stage, and the machine in-built temperature monitors was $\leq$0.5°C. Note that the temperature readouts could differ from the temperature set on the controller even after equilibrium, particularly close to room temperature, so we used the thermometer and temperature monitors' readouts as the true temperature of the experiments. The temperatures of the five experiments were 25, 27, 30, 33, and 37°C, and the uncertainty of our temperature measurements was estimated to be $\pm$1°C by comparing the temperature differences at various locations of the compartment. Because of the machine taking a substantial amount of time to change temperature, distinct experiments were performed at different temperatures using different cells (the same cell line but different individual cells in each well on sometimes different days).

Postprocessing experimental data

We performed a series of quality control checks and corrections (in postprocessing) to ensure the currents recorded represent only I_{Kr}. Leak corrections were applied to all measurements to eliminate leak current (16). E-4031 subtraction was applied to remove any native voltage-dependent ion currents that were present in CHO cells besides the overexpressed hERG1a, usually known as endogenous currents. Cells were then selected based on partially automated quality control described in Part I of this article (16), resulting in $N_e = 124$, 91, 85, 84, and 45 cells being selected for measurements at 25, 27, 30, 33, and 37°C, respectively, and our 25°C data were examined in Part I (16). The lower yield of cells at higher temperatures was mostly due to reduced success in the cell capture step, before any recording started, and to a lesser extent, the deterioration of the patch clamp. The full analysis of which quality control criteria removed cells at the various temperatures is shown in Supporting Materials and Methods, Section S12.

Data visualization

Each hERG-transfected CHO cell was expected to have a different total conductance, hence giving a different magnitude for the current recording. Therefore, normalization was applied for visual comparison. Note that the validation of model predictions was performed without normalization (a conductance was fitted for each cell individually). To avoid any circular reasoning involved in normalizing based on the g_{Kr} parameter fit within the models (which, at this point, may or may not vary with temperature), we used an experimental maximal conductance estimate. The experimental estimate is approximated by extrapolating the negative tail current, after the first +40 to −120 mV step, back to the time the voltage step occurred (see Fig. S1). Note that this normalization method is imperfect as it relies on a particular gating process (activation gate $a \approx 1$ at the end of the +40 mV step), which has some dependence on the kinetics we aim to compare, but the 22°C parameterization of the model (45) suggests $a \approx 1$ is a reasonable approximation (even for lower temperatures) at this point in the protocol. However, because this method removes the conductance dependency, it has a benefit over the normalization-to-a-reference-trace method used in Lei et al. (16) by preserving the different magnitudes of currents from different temperatures.

Mathematical model

We used the same Hodgkin and Huxley-style structure hERG model described in Lei et al. (16) and in Beattie et al. (45). In this model, the current, I_{Kr}, is modeled with a standard Ohmic expression as follows:

$$I_{Kr} = g_{Kr} \cdot a \cdot r \cdot (V - E_K), \tag{13}$$

where g_{Kr} is the maximal conductance, a is a Hodgkin and Huxley (17) activation gate, and r is an inactivation gate. E_K is the reversal potential, also known as the Nernst potential, which is not inferred but is calculated directly using the following:

$$E_K = \frac{RT}{zF} \ln\left(\frac{[K^+]_o}{[K^+]_i}\right), \tag{14}$$

where R is the ideal gas constant, T is the absolute temperature, F is the Faraday constant, and z is the valency of the ions (equal to 1 for K^+). $[K^+]_o$ and $[K^+]_i$ denote the extracellular and intracellular concentrations of K^+, respectively, which are determined by the experimental solutions, 4 and 110 mM, respectively. The two gates are governed by the following:

$$\frac{da}{dt} = \frac{a_\infty - a}{\tau_a}, \quad \frac{dr}{dt} = \frac{r_\infty - r}{\tau_r}, \tag{15}$$

$$a_\infty = \frac{k_1}{k_1 + k_2}, \quad r_\infty = \frac{k_4}{k_3 + k_4}, \tag{16}$$

$$\tau_a = \frac{1}{k_1 + k_2}, \quad \tau_r = \frac{1}{k_3 + k_4}, \tag{17}$$

where

$$k_1 = p_1 \exp(p_2 V), \quad k_3 = p_5 \exp(p_6 V), \tag{18}$$

$$k_2 = p_3 \exp(-p_4 V), \quad k_4 = p_7 \exp(-p_8 V). \tag{19}$$

Therefore, our model consists of nine positive parameters $\boldsymbol{\theta} = \{g_{Kr}, p_1, \ldots, p_8\}$, each of which is to be inferred from the experimental current recordings.

Simulations were run using Myokit (46), with tolerance settings for the CVODE solver (47) set to abs_tol = 10^{-8} and rel_tol = 10^{-10}. All codes and data are freely available at https://github.com/CardiacModelling/hERGRapidCharacterisation, a permanently archived version is available at https://doi.org/10.6084/m9.figshare.9677369.v1.

Independent parameter fits at each temperature

The fitting procedure described briefly here follows exactly that laid out in Part I (16) but is repeated for each of the five temperatures.

First, we defined a transformation $\boldsymbol{\phi} = \ln(\boldsymbol{\theta})$ to turn our positively constrained model parameters into unconstrained parameters. For each temperature, we specified a statistical model to relate the mathematical model and the observed experimental data:

$$I_{Kr}^{data} = I_{Kr}^{model} + \epsilon, \tag{20}$$

where we assumed the noise term ε follows a normal distribution $\varepsilon \sim N(0, \sigma^2)$. Writing $\mathbf{y} = \{y_k\}$ for the experimental data (I_{Kr}^{data}) and $\mathbf{z} = \{z_k\}$ for a simulated vector (I_{Kr}^{model}), the likelihood of observing a data set $\mathbf{y}$, given $\boldsymbol{\phi}$, is as follows:

$$p(\mathbf{y} \mid \boldsymbol{\phi}, \sigma) = \frac{1}{\sqrt{2\pi\sigma^2}} \exp\left(-\sum_k \frac{(z_k(\boldsymbol{\phi}) - y_k)^2}{2\sigma^2}\right). \tag{21}$$

Bayes' theorem can then be applied to calculate the likelihood of a parameter set given experimental data as follows:

$$p(\boldsymbol{\phi}, \sigma | \mathbf{y}) = \frac{p(\boldsymbol{\phi}) p(\mathbf{y} \mid \boldsymbol{\phi}, \sigma)}{p(\mathbf{y})} \propto p(\boldsymbol{\phi}) p(\mathbf{y} \mid \boldsymbol{\phi}, \sigma), \tag{22}$$

with the prior

$$p(\boldsymbol{\phi}) \sim \mathcal{U}(\boldsymbol{\phi}^{min}, \boldsymbol{\phi}^{max}), \tag{23}$$

where $\mathcal{U}(\cdot)$ represents a uniform distribution (for details see Lei et al. (16)).

For each temperature T, we combined multiple experimental recordings using a hierarchical Bayesian model, as in Lei et al. (16). The full hierarchical Bayesian likelihood is given by the following:

$$\mathcal{L}\left(\boldsymbol{\mu}, \boldsymbol{\Sigma}, \{\boldsymbol{\theta}_j, \sigma_j\}_{j=1}^{N_e} \,\middle|\, \{\mathbf{y}_j\}_{j=1}^{N_e}\right) \propto \prod_{j=1}^{N_e} p(\mathbf{y}_j \mid \boldsymbol{\theta}_j, \sigma_j)$$

$$\times\, p\left(\{\boldsymbol{\theta}_j\}_{j=1}^{N_e} | \boldsymbol{\mu}, \boldsymbol{\Sigma}\right) \times p(\boldsymbol{\mu}, \boldsymbol{\Sigma}) \times \prod_{j=1}^{N_e} p(\sigma_j), \tag{24}$$

where $\boldsymbol{\mu}$ and $\boldsymbol{\Sigma}$ are the hyperparameters of the hierarchical model representing the mean vector and covariance matrix from which the individual "low-level" (well-specific) parameters are drawn. $\{\boldsymbol{\theta}_j, \sigma_j\}_{j=1}^{N_e}$ are the set of individual "low-level" parameters for each of the N_e repeats of the experimental recordings $\{\mathbf{y}_j\}_{j=1}^{N_e}$. The four terms in Eq. 24 correspond to 1) the likelihood of all the individual (low-level) experiments, 2) the likelihood of the hyperparameters (top-level), 3) the prior distribution of the hyperparameters, and 4) the prior distribution of σ_j.

We assumed $\boldsymbol{\phi}_j$ for a particular cell (experiment) j follows a multivariate normal distribution, namely $\boldsymbol{\phi}_j \sim \mathcal{N}(\boldsymbol{\mu}, \boldsymbol{\Sigma})$. Two distributions include variability across wells in this hierarchical Bayesian model: they are described by samples of the mean parameter vector $\boldsymbol{\mu}$, and the covariance matrix $\boldsymbol{\Sigma}$. As described in the discussion of Lei et al. (16), if we believe the well-well variability represented by $\boldsymbol{\Sigma}$ is primarily due to different patch-clamp artifacts in each well, then the uncertainty in $\boldsymbol{\mu}$ represents our uncertainty in the underlying physiology, and we therefore believe it corresponds to our uncertainty in the physiological hERG temperature response rather than our expected variability in the results of future experiments, which would require $\boldsymbol{\Sigma}$ too.

For the choice of likelihoods, prior distributions, and sampling algorithms, we used the simplified pseudo-Metropolis within Gibbs (MwG) algorithm introduced in Part I ((16); Supporting Material and Methods, Section 6). All inference and sampling were done via our open source Python package, PINTS (48); the code is provided as described above.

Fitting Eyring and Q_{10} relationships

To investigate how well the two temperature models, the Generalized Eyring and the Q_{10} relationships, can explain the temperature dependency of hERG kinetics, we fitted the two temperature models to the inferred distribution of the mean parameter vector $\boldsymbol{\mu}(T)$ for all temperatures T. To do so, first, we transformed both the temperature models and $\boldsymbol{\mu}(T)$ to the Eyring plot form (see Fig. 1). Second, we modeled the marginal distribution of $\boldsymbol{\mu}(T)$ of p_i at each T in the Eyring plot using a normal distribution with mean $\overline{\mu}_i(T^{-1})$ and standard deviation (SD) $\sigma_{\mu,i}(T^{-1})$. We further assumed both $\overline{\mu}_i(T^{-1})$ and $\sigma_{\mu,i}(T^{-1})$ follow the temperature models, given by Eqs. 7 and 8 for the Generalized Eyring relationship and Eqs. 11 and 12 for the Q_{10} formulation.

Finally, given $\overline{\mu}_i(T^{-1})$ and $\sigma_{\mu,i}(T^{-1})$ (Fig. S8), we applied linear regression for parameters A and B in the Generalized Eyring model (Eqs. 7 and 8 to infer a_{GE}, b_{GE}, c_{GE}, and d_{GE}) and a least-squares method for only parameters A in the Q_{10} relationship (Eq. 11 to infer a_{Q10} and c_{Q10}) with the Levenberg-Marquardt algorithm provided in SciPy (49): once to fit the mean and once to fit the SD of each parameter as a function of temperature. Because of the simplicity of the problem after our transformation, a relatively simple optimization algorithm was sufficient. For the constant B parameter in the Q_{10} relationship (Eq. 12), we followed the standard way of using a Q_{10} relationship in which rates are extrapolated from room temperature. Therefore, we extrapolated to other temperatures using $\overline{\mu}_i(T^{-1})$ and $\sigma_{\mu,i}(T^{-1})$ at $T = 25°\mathrm{C}$.

The estimated mean as a function of temperature was used to perform predictions for each temperature model; the estimated SD as a function of temperature allowed us to compute the uncertainty bounds for the I_{Kr} model parameters for each temperature model.

RESULTS

Temperature dependence of recordings

Fig. 2 shows the normalized voltage clamp recordings measured with the nine different protocols, and the corresponding voltage protocols, at the five temperatures. Each panel, from top to bottom, shows the voltage clamp protocol (black) then the normalized recordings (blue) that passed quality control at 25, 27, 30, 33, and 37°C, respectively. All results shown are the first of the two repeats of our recordings.

Fig. 2 *A* shows the staircase calibration protocol (in black) and the corresponding experimental recordings (in blue). The change in the recorded current as temperature increased was prominent. It increased the size of the current but also highlighted alterations to the kinetics. During the first half (3–8 s) of the staircase protocol, at low temperature, there was almost no current recorded; however, at physiological temperature, the current was almost as big as the current recorded during the second half (8–13 s) of the staircase protocol. Furthermore, the shape of the current during the second half (8–13 s) of the staircase protocol also changed as temperature increased. This demonstrates that the staircase protocol contains useful information on how kinetics change with temperature.

Fig. 2, *B*–I shows experimental recordings for the other eight validation protocols from the same cells. In validation protocol 1 (Fig. 2 *B*), we saw the activation I-V curve shifting to a lower voltage at higher temperatures. In validation protocol 3 (Fig. 2 *D*) and validation protocols 6–9 (Fig. 2, *G*–I), larger hERG currents were observed at higher temperatures. Both these responses for hERG have been reported previously (3).

Temperature-dependent fits and predictions

In Lei et al. (16), we showed exclusively the quality of fits and predictions for the hERG models at 25°C as this could be most easily compared with previous manual patch results (45); the models replicated both the experimental training and validation data very well.

Fig. 3 shows the model fitting and validation results for all recorded cells at 37°C alongside the experimental recordings measured under the nine different protocols. We fitted the model to the staircase protocol (Fig. 3 *A*) and validated against the other eight protocols (Fig. 3, *B*–I). To visually compare the variability in hERG kinetics (and not conductance), currents are normalized by scaling them to minimize the absolute difference between each trace and a reference trace (as in (16)). Similar plots for all the intermediate temperatures are shown in Figs. S2–S4.

We applied the same error measure as in Part I of the study to quantify the fits and predictions—the relative root mean-square error (RRMSE), defined as follows:

$$\mathrm{RRMSE} = \sqrt{\sum \left(I_{\mathrm{Kr}}^{\mathrm{model}} - I_{\mathrm{Kr}}^{\mathrm{data}}\right)^2 \Big/ \sum \left(I_{\mathrm{Kr}}^{\mathrm{data}}\right)^2}. \quad (25)$$

Here, $I_{\mathrm{Kr}}^{\mathrm{model}}$, $I_{\mathrm{Kr}}^{\mathrm{data}}$ are the model predictions and recordings of I_{Kr}, respectively. Fig. 4 shows the RRMSE histograms for all cells and for the six current trace protocols at 37°C. Markers indicate the best (*), median (‡), and 90th percentile (#) RRMSE values, and corresponding raw traces and

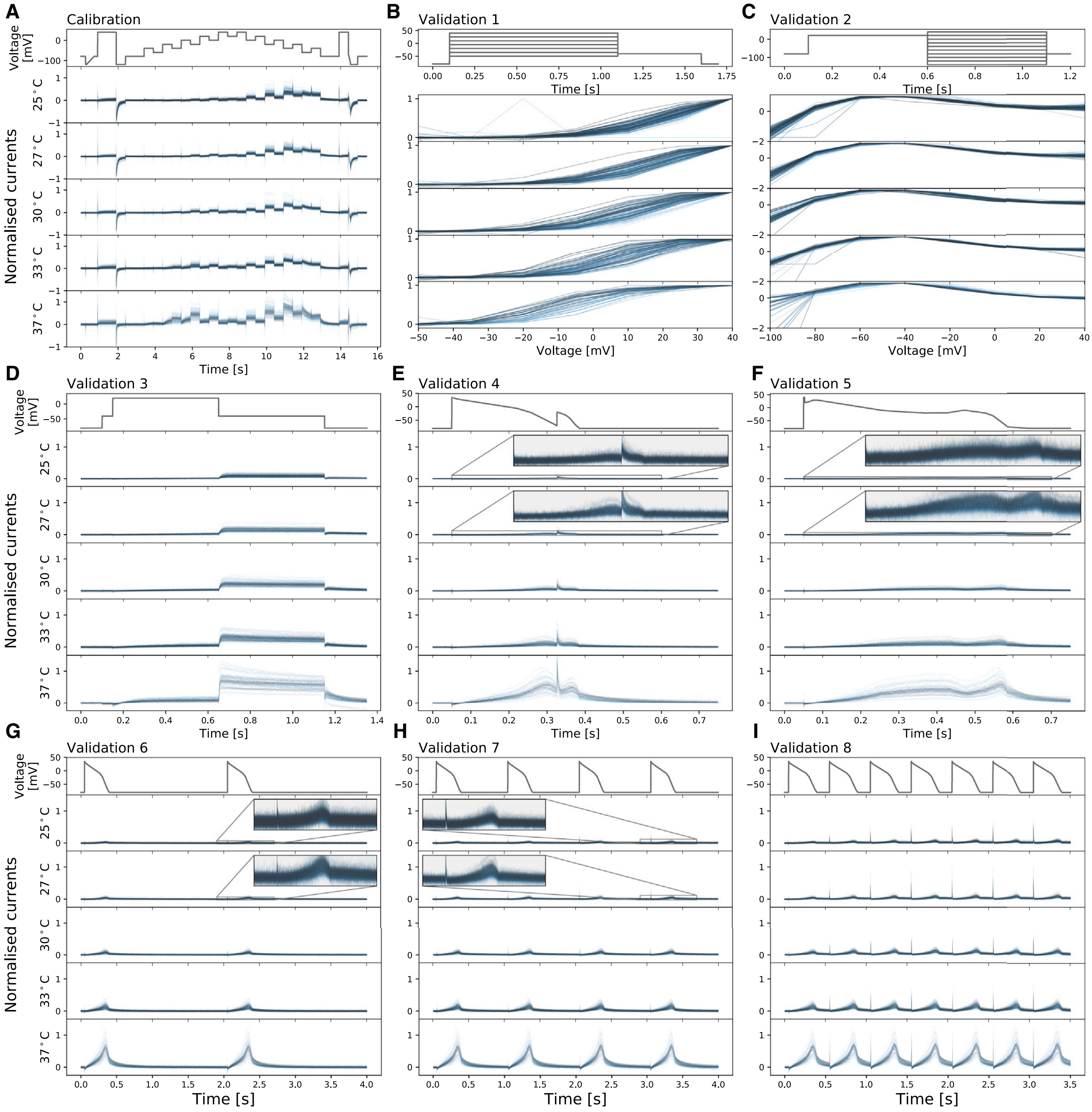

FIGURE 2 Whole-cell patch-clamp voltage clamp recordings under nine different protocols, which were all measured in each cell, at five temperatures. Each panel, from top to bottom, shows the voltage clamp protocol (*black*) and normalized current recordings (*blue*) that passed quality control at 25, 27, 30, 33, and 37°C, respectively. Currents were normalized with the method described in the text (see Fig. S1). (*A*) The calibration protocol and the staircase protocol are shown. (*B–I*) Shown are the eight different protocols used as validation of the model calibration, which are the activation current-voltage (I-V) protocol, the steady-state inactivation I-V protocol, the hERG screening protocol, the DAD-like protocol, the EAD-like protocol, and the cardiac action potential-like protocol at 0.5, 1, and 2 Hz, respectively. In (*B* and *C*), validation 1 and 2 show the I-V relations extracted from the currents. To see this figure in color, go online.

predictions are shown in the three panels above. The same analysis is presented for the remaining protocols in Fig. S16. We note that the models only show single exponential decays because of the limitations of the model structure, whereas the data seem to show double exponential decays. These results demonstrate that the hERG model remains a very good representation of the current kinetics, even at 37°C, the highest temperature. The same analysis has been applied to the intermediate temperatures; the results are shown in Figs. S5–S7 and S13–S15.

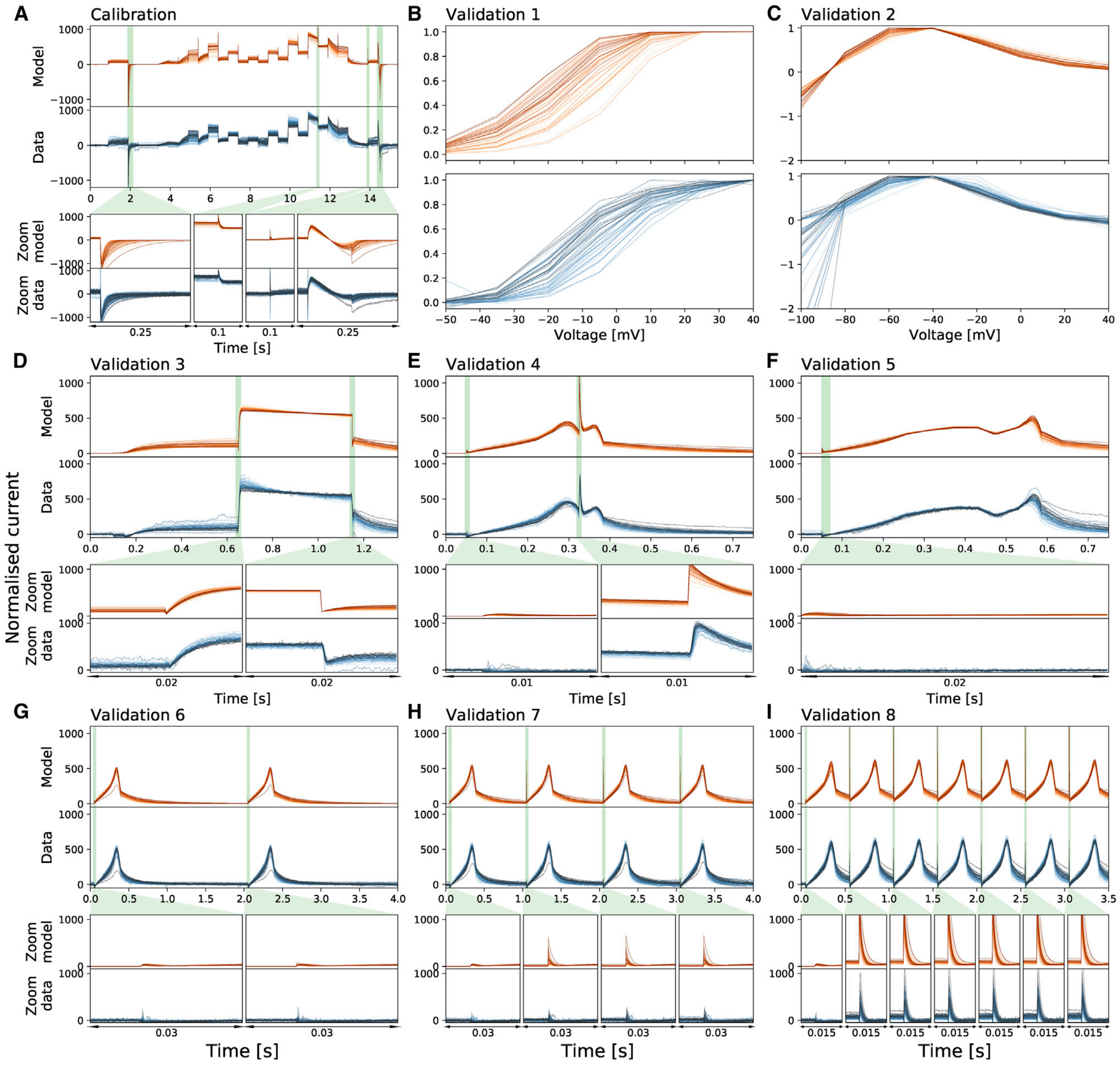

FIGURE 3 Whole-cell patch-clamp voltage clamp recordings under nine different protocols and the model fitting and validation results at 37°C. All currents are normalized by scaling them to minimize the absolute difference between each trace and a reference trace. From (*A*) to (*I*): Shown are the results of the staircase protocol, which is used as the calibration protocol, the activation current-voltage (I-V) protocol, the steady-state inactivation I-V protocol, the hERG screening protocol, the DAD-like protocol, the EAD-like protocol, and the cardiac action potential-like protocol at 0.5, 1, and 2 Hz, respectively. All the model calibration results and validation predictions are shown in the top panels (*orange*) and are compared against the experimental recordings shown in the bottom panels (*blue*). Zoomed-in of the green shaded regions are shown underneath each panel to reveal the details of the spikes, in which our models show extraordinary good predictions to the details. The normalized current for all protocols are shown except for the activation I-V protocol and the steady-state inactivation I-V protocol in which the summary statistic I-V relationships are shown. Each cell is shown with a unique color. To see this figure in color, go online.

Temperature dependence of inferred model parameters

Fig. 5 shows the inferred parameter values, which are used in the model predictions in Figs. 3 and S2–S4, as a function of temperature. The figure shows the inferred distribution of the hyperparameter mean vector $\boldsymbol{\mu}$ (Eq. 24) using the simplified pseudo-MwG at each temperature in a violin plot. The mean values and 95% credible intervals of the hyperparameter mean vector $\boldsymbol{\mu}$ for all temperatures are provided in Tables S2 and S3.

If the model kinetics were exhibiting temperature dependence following Q_{10} or Eyring rate theory, then lines whose

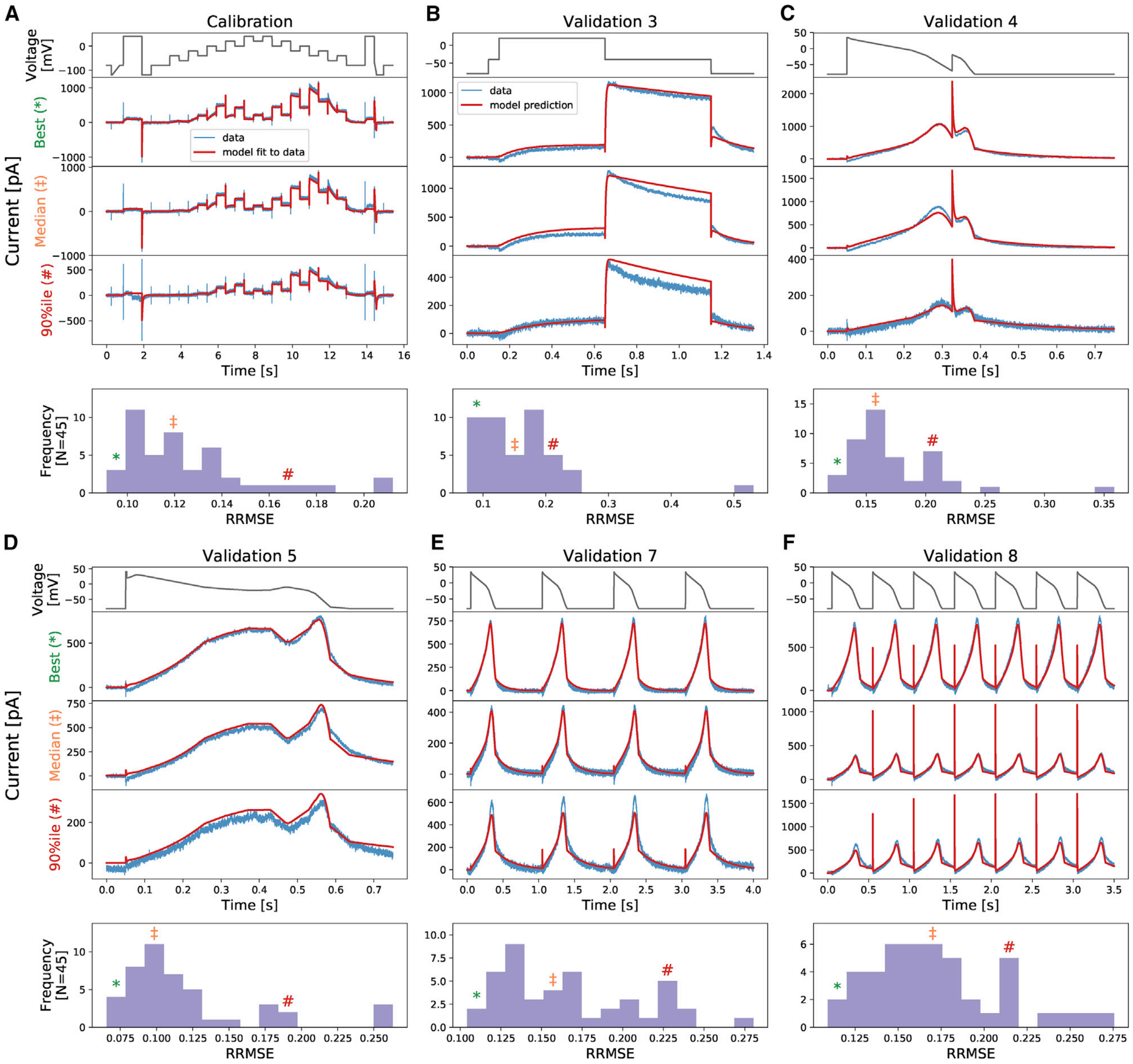

FIGURE 4 The relative root mean-square error (RRMSE, given by Eq. 25) histograms for all cells and for six protocols (*A–F*) at 37°C. Markers indicate the best (*), median (‡), and 90th percentile (#) RRMSE values. The raw traces with the best, median, and 90th percentile RRMSE values, for both the model (*red*) and data (*blue*), are shown in the panels above, together with the voltage protocol shown on top. Note that the currents are shown on different *y* axis limits to reveal the details of the traces. The same analysis is presented for the remaining protocols in Fig. S16. To see this figure in color, go online.

function is specified by these principles would fit the inferred parameters in Fig. 5.

In Fig. 5, most parameters show an obvious monotonic trend as temperature increases, although a handful take a slightly more complicated form. It is obvious that the *B* parameters in the second row, p_i with even i, are not constant over temperatures as would be expected from the Q_{10} relationship. An Eyring plot version of Fig. 5 is shown in Fig. S8. We will compare these inferred parameters with the theoretical relationships in detail in the next section.

We then applied Eqs. 16 and 17 to calculate the steady states a_∞ and r_∞ and time constants τ_a and τ_r at the five temperatures, using the mean of the inferred distribution of $\boldsymbol{\mu}$ at each temperature. Fig. 6 shows the resulting voltage dependency of the steady states and time constants of the model gates a and r, in which each temperature is indicated by a different color (25°C, blue to 37°C, red).

Fig. 6 shows that as temperature increases, the steady state of the activation gate a shifts in a negative voltage direction, a prediction from the fitted model that is in agreement with the experimental observations in validation

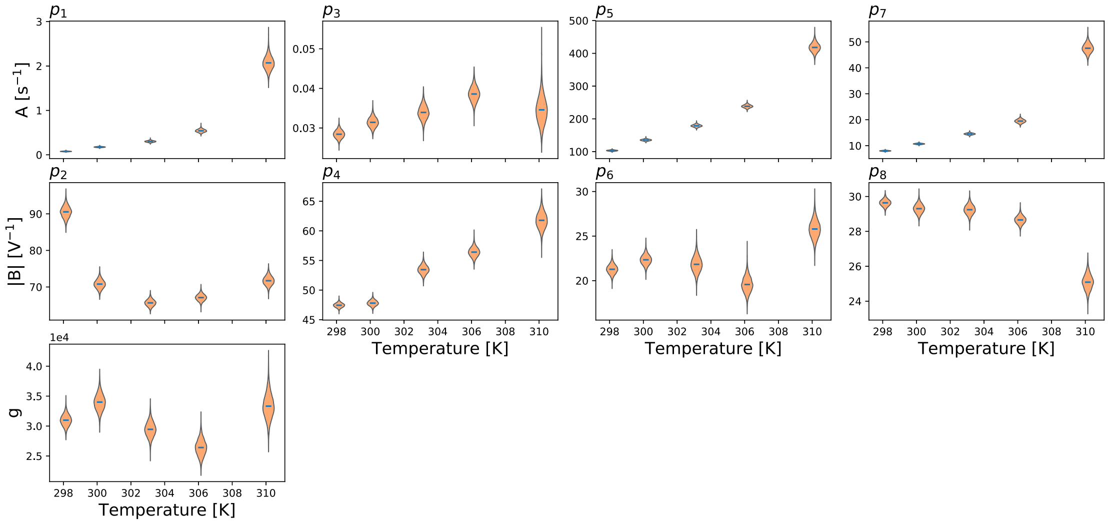

FIGURE 5 Model parameters plotted as a function of temperature. Here, only the inferred distribution of the hyperparameter mean vector $\boldsymbol{\mu}$ (Eq. 24) using the simplified pseudo-MwG at each temperature is shown. Parameters A and B refer to Eq. 2. Model parameters show different degrees of temperature dependency. The conductance g does not show a prominent change as the temperature increases.

protocol 1; the voltage of half-maximal activation ($V_{1/2}$) of a_∞ shifts from 7.5 mV at 25°C to 30.9 mV at 37°C, without a noticeable change in the slope factor. However, the steady state of the inactivation gate r does not show a prominent change over temperatures.

The time constant of both gates τ_a and τ_r show a similar effect as temperature increases; the maximal τ_a drops from 13.2 s at 25°C to 2.2 s at 37°C, and the maximal τ_r drops from 14.3 ms at 25°C to 3.6 ms at 37°C. Note that τ_a is in the order of seconds, whereas τ_r is in milliseconds. The voltage that maximizes the time constant shifts from 11.6 mV at 25°C to 31.7 mV at 37°C for the activation gate, although it does not show a noticeable change for the inactivation gate.

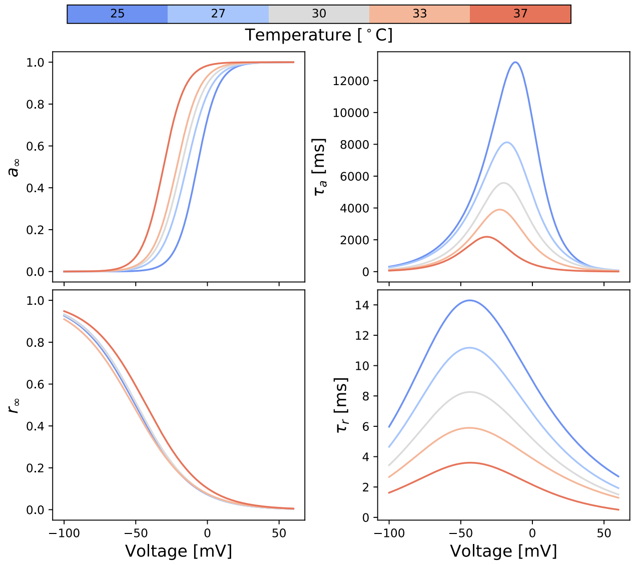

FIGURE 6 Predicted voltage dependency of steady states and time constants of the model gates a and r at different temperatures. These lines are calculated directly from inferred parameters using Eqs. 16 and 17 with the independently fitted hierarchical Bayesian model mean values. To see this figure in color, go online.

We compared the model given by the mean of the posterior for $\boldsymbol{\mu}$ at 37°C (Table S2) with existing I_{Kr} models from within action potential models by using the Cardiac Electrophysiology Web Lab (50,51). The CellML description (www.cellml.org (52)) is available in the Supporting Materials and Methods. Interestingly, the new model shows a striking concordance for predicted current under action potential clamps with the Markov model by Fink et al. (14); results are shown in Fig. S21.

Comparing models of temperature dependence

Fig. 7 shows the Generalized Eyring relationship and the Q_{10} equation fitted to the inferred parameters shown in Fig. 5 (orange violin plot). The results are shown in the Eyring plot form: $\ln(A/T)$ and $|B|$ as functions of T^{-1}. A version of Fig. 7 with model parameters plotted directly against the temperature is shown in Fig. S9. The Generalized Eyring fits are shown as green fan charts with the first three SDs; the Q_{10} fits are shown similarly in red. The obtained parameters for the Generalized Eyring equation (Eq. 4) and the Q_{10} equation (Eq. 9) are given in the bottom right tables, one set for each rate k_i, for $i = 1$, 2, 3, and 4. Reassuringly, the values in the tables are comparable to (the same orders of magnitude as) typical literature values for ion channel models (1,3,4,14,19,23).

From the illustration in Fig. 1, we expect the Generalized Eyring and Q_{10} formulations to be indistinguishable for the A parameters, and indeed, in Fig. 7, the green fan charts (Generalized Eyring) are on top of the red fan charts (Q_{10})

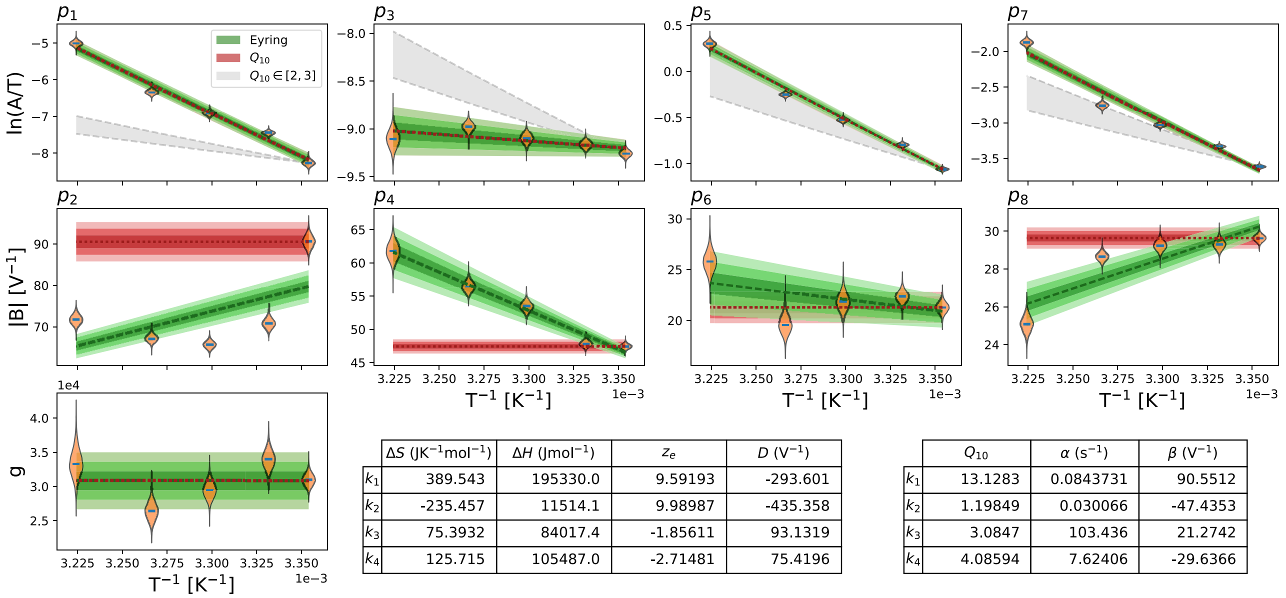

	ΔS (JK^{-1}mol^{-1})	ΔH (Jmol^{-1})	z_e	D (V^{-1})
k_1	389.543	195330.0	9.59193	-293.601
k_2	-235.457	11514.1	9.98987	-435.358
k_3	75.3932	84017.4	-1.85611	93.1319
k_4	125.715	105487.0	-2.71481	75.4196

	Q_{10}	α (s^{-1})	β (V^{-1})
k_1	13.1283	0.0843731	90.5512
k_2	1.19849	0.030066	-47.4353
k_3	3.0847	103.436	21.2742
k_4	4.08594	7.62406	-29.6366

FIGURE 7 Fitting of Generalized Eyring equation and Q_{10} equation to the distribution of the mean parameter values (mean over all wells, $\boldsymbol{\mu}$, shown with an *orange* violin plot) on the Eyring axes. The obtained Generalized Eyring fits are shown as green fan charts with the first three standard deviations; the obtained Q_{10} fits are shown in red. The fitted parameters for the Generalized Eyring and Q_{10} equations are shown in the bottom right tables, one set for each k_i, with $i = 1$, 2, 3, and 4. For Q_{10} equations, $T_{ref} = 298.15$ K was used. Note that the nonzero estimations of D in the Generalized Eyring relationship indicate that the Typical Eyring cannot fit to all B parameters as it is required to go through the origin. For comparison to typical Q_{10} values in literature, in which Q_{10} values are commonly assumed to be around 2 to 3, we show a $Q_{10} \in [2, 3]$ relationship with the gray shaded region. To see this figure in color, go online.

in the first row; both formulations are able to fit to the model's inferred A parameters.

Fig. 7 shows that the Generalized Eyring equations fit better to the inferred B parameters than the Q_{10} equations. The Generalized Eyring equations are able to fit the inferred model parameters to a large extent, except for p_2, whereas the B parameters in the Q_{10} equations are not temperature dependent (by definition), which is contradicted by our observations.

Furthermore, it is evident that for parameters p_4 and p_6, the two lines cannot intercept the y axis close to the origin because they are decreasing rather than increasing on these plots. Parameters p_2 and p_8 also have nonzero estimates of D in the Generalized Eyring relationship, indicating that the Typical Eyring relationship cannot be fit to any of our B parameters. The example shown earlier in the bottom panel of Fig. 1 is based on the Generalised Eyring, Typical Eyring and Q_{10} fits for k_4 (p_7 and p_8) shown in Fig. 7. The gradient of the Generalized Eyring fit is approximately twice as steep as the Typical Eyring fit would require for p_8.

In the literature, Q_{10} coefficients for biological processes such as channel gating are commonly thought to take values from around 2 to 3 (53). To investigate this assumption, we projected our 25°C model parameters directly using Eq. 9 with $Q_{10} \in [2,3]$, which is shown as the gray shaded region in Fig. 7. Parameter p_5 in the inactivation rate (k_3) gives a Q_{10} just above 3, but none of our other inferred relationships for parameter A is close to the range $Q_{10} \in [2,3]$.

We further assess the performance of the temperature dependence models by comparing their mean model predictions against the data and the temperature-specific models. Fig. 8 shows the mean model predictions from the temperature-specific parameters (orange), the Generalized Eyring formulation (dotted green), and the Q_{10} coefficient (dashed red) for the staircase protocol. All predictions are generated with the same mathematical model Eq. 13, where the rate constants in Eqs. 16 and 17 are replaced by Eq. 5 (for the Generalized Eyring formulation) and Eq. 9 (for the Q_{10} coefficient) computed with the inferred parameters shown in the tables of Fig. 7, with Q_{10}-based predictions based on extrapolation from 25°C. The top panel shows the staircase protocol, followed by the normalized current at five different temperatures. Data (in Fig. 2 *A*) are shown in fan chart style with the 30th, 60th, and 90th percentiles in blue. At low temperatures, all three models agree with the data. At higher temperatures, particularly at 37°C, the predictions from the Generalized Eyring formulation (dotted green) still agree reasonably with the temperature-specific independently fitted parameters (orange), and both fit the data (blue) well. However, the prediction from the Q_{10} coefficient deviates from the data during the spikes (see zoomed-in images on the right) and does not predict the time course accurately during 4–7 and 12–13 s of the staircase protocol (see insets in Fig. 8).

Fig. 9 shows a 2 Hz action potential-like protocol prediction version of Fig. 8. All the three mean models are able to

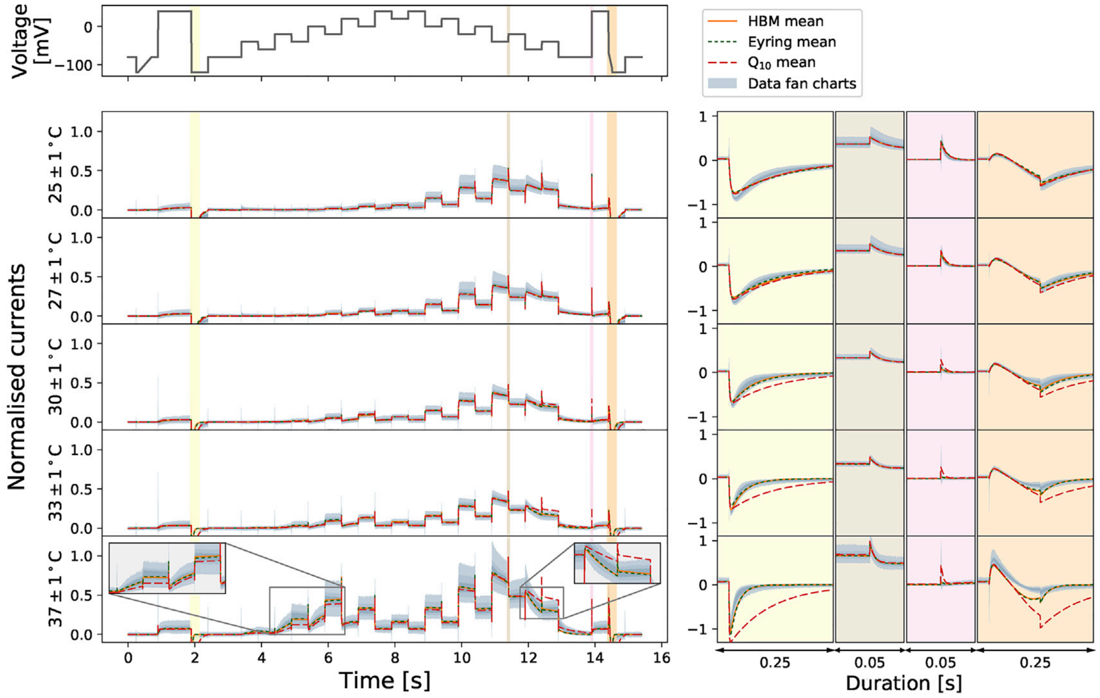

FIGURE 8 Comparison of the Generalized Eyring formulation (*dotted green*) and Q_{10} coefficient (*dashed red*) mean predictions for the staircase protocol. Top figure shows the staircase protocol, followed by the normalized current at five different temperatures. Data (in Fig. 2 *A*) are shown in fan charts style with the 90th, 60th, and 30th percentiles in blue. The mean prediction from the hierarchical Bayesian model (HBM) is shown in orange. Zoomed-in regions are shown on the right with colors matching the highlighted regions of the main plots on the left. To see this figure in color, go online.

predict the current during the repolarization of the action potential clamp very well. The spikes during the upstrokes are, however, badly predicted by the Q_{10} coefficient mean model, whereas the Generalized Eyring formulation, similar to the temperature-specific parameters, gives a prediction closer to the data.

DISCUSSION

In this study, we have examined the temperature dependence of hERG kinetics, at five temperatures ranging from room to body temperature, with 45–124 cells per temperature. We have used a mechanistic model and its parameterization to capture our knowledge of the hERG kinetics. By assuming that all cells share the same mechanism underlying hERG kinetics, we have based our study on the inferred model parameters at different temperatures to reveal the temperature dependence of hERG gating kinetics. This is, to our knowledge, the first systematic effort to have taken this approach.

Using the staircase protocol, we were able to characterize hERG kinetics to the extent that our model can replicate both the experimental training and validation data very well for all of the measured temperatures. Our models can predict the current response to the physiologically relevant action potential protocols with a very high accuracy, demonstrating that our I_{Kr} models are robust in predicting hERG current, in both healthy and arrhythmic situations. This gives us confidence that the cell-specific model parameters do represent and capture hERG kinetics at the given temperatures.

The directly fitted models reveal that the activation gate has a much higher temperature sensitivity than the inactivation gate. This effect is shown in both the comparison of steady states and time constants (Fig. 6) and the inferred Q_{10} coefficients (Fig. 7) in which the Q_{10} values for the activation gate (k_1 and k_2) are overall higher than the inactivation gate (k_3 and k_4). Our inferred Q_{10} coefficient for the rate of activation (k_1) is relatively high compared to literature results (3,4). However, our findings are not implausible when compared to other potassium channels, such as Kv2.1 and Kv4.3, which can have maximal Q_{10} values up to the 20–30 range (2). Other ion channels can also exhibit a very high temperature sensitivity, such as transient receptor potential ion channels, which were reported to have Q_{10} values ranging from 2 to 15 in Dhaka et al. (1). We then further compare our model predictions with the literature results in Vandenberg et al. (3).

Our hierarchical Bayesian models at different temperatures are not only able to predict our validation data but also able to reproduce the temperature dependence seen in previous studies (3), in which the increase of temperature caused a large increase in the overall "steady-state open probability." In Supporting Materials and Methods, Section

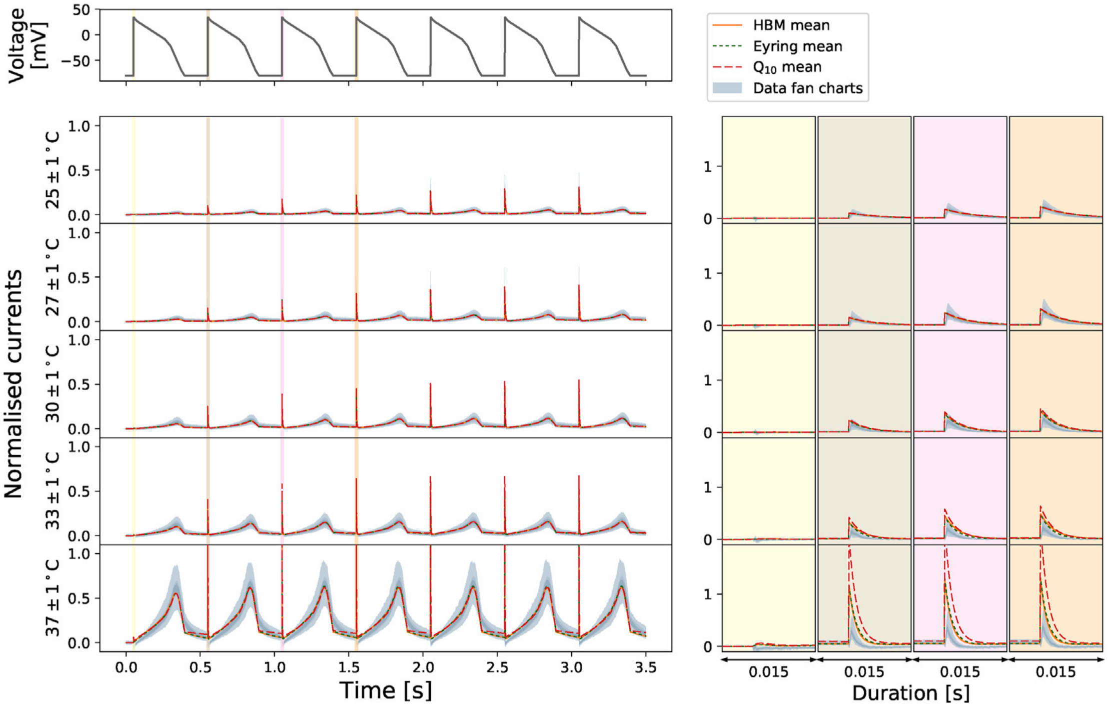

FIGURE 9 Comparison of the Generalized Eyring formulation (*dotted green*) and Q_{10} coefficient (*dashed red*) mean predictions for the 2 Hz action potential-like protocol. Top figure shows the staircase protocol, followed by the normalized current at five different temperatures. Data (Fig. 2 *A*) are shown in fan charts style with the 90th, 60th, and 30th percentiles in blue. The mean prediction from the hierarchical Bayesian model (HBM) is shown in orange. Zoomed-in regions are shown on the right with colors matching the highlighted regions of the main plots on the left. To see this figure in color, go online.

S8, we describe how we reproduced Fig. 6 of Vandenberg et al. (3). Fig. 10 shows that our simulations (right panel) are broadly consistent with the temperature effect observed in Vandenberg et al. (3) (left panel). The fan charts show the 30th, 60th, and 90th percentiles of the simulations, representing the inter-experiment (well-well) variability. There are differences between our simulations and their experimental results, with a smaller open probability at low temperatures in our simulations and a slight shift of the curves to the right. Nevertheless, our results are broadly consistent with the temperature effect observed in Vandenberg et al. (3) and predict a very similar "width" for this steady-state window of open probability and also agree with the absolute values of the probabilities at the higher temperature very well.

Q_{10} formulations have often been estimated in the past with different protocols, even for the same gating process (e.g., activation). For example, two well-known experimental studies of temperature dependence of hERG kinetics, by Zhou et al. (4) and Vandenberg et al. (3), estimated the Q_{10} coefficients using different protocols and analyses and reported two different sets of Q_{10} coefficients (see Table 1) for various gating processes. We asked the following question: if the two experiments were to be repeated with the same underlying kinetics, would they agree with one another? Using our directly fitted models at 25 and 37°C, we simulated the two different sets of experiments described in (3,4) (for details, see Supporting Materials and Methods, Section S9). We then estimated two sets of Q_{10} coefficients following the protocols and analysis in each of the articles, and the obtained values are shown in Table 1. The findings in Table 1 show strong evidence that because of different protocols, the estimated Q_{10} coefficients can disagree. Furthermore, neither of the protocols reproduces the direct estimate of Q_{10} coefficients from the model parameter temperature relationships (shown in the bottom right of Fig. 7). We conclude that extreme caution should be used when directly modifying rates in models with experimental estimations of Q_{10} coefficients.

Fitting directly to the staircase protocol at different temperatures does not require any assumption about the underlying temperature dependence of the kinetic parameters, except that the model structure does not change. The existing well-known models/approximations for temperature dependence of ion channel transition rates are the Q_{10} and Typical Eyring formulations. Our study has raised concerns about how accurate these relationships are. In terms of parameter values (Fig. 7), neither of these methods is able to capture the full temperature dependence of the directly fitted parameters, $\boldsymbol{\mu}(T)$, and predictably, this impairs their ability to fit and predict currents (Fig. 8). However, using a Generalized Eyring relationship (not commonly used in ion channel modeling) can closely mimic our full direct fitting approach (Figs. 8 and 9). Although the model

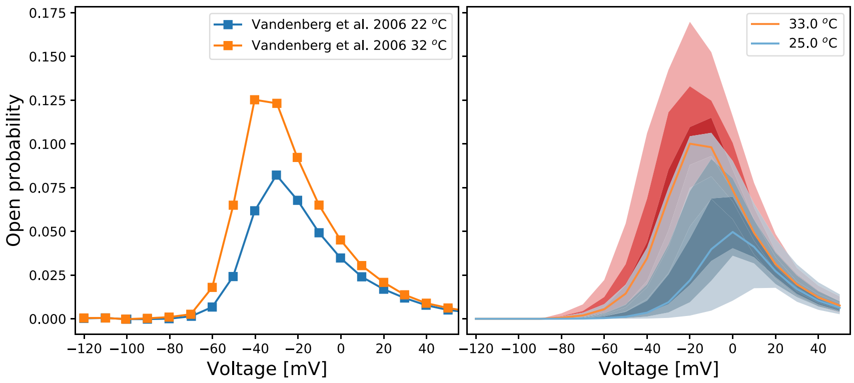

FIGURE 10 Voltage dependence of steady-state "open probability" as defined in Vandenberg et al. ((3), Fig. 6) using a multiplication of experimental approximations for the product $a_\infty r_\infty$. Left: Data were extracted from Vandenberg et al. ((3), Fig. 6). Right: The fan charts show the 90th, 60th, and 30th percentiles of the hierarchical Bayesian model simulations, representing the experiment-experiment variability. Orange/red represents 32 or 33°C, and blue represents 22 or 25°C in the respective studies. To see this figure in color, go online.

predictions using the Q_{10} formulation can generally predict overall trends in temperature effects, the predictions cannot capture the details of the current compared to the Generalized Eyring relationship or the full direct fitting approach (Figs. 8 and 9). We therefore suggest neither Q_{10} formulations nor the Typical Eyring relationship should be used; the Generalized Eyring relationship is much better for temperature predictions. But for the best results, the model should be refitted at any temperature of interest using an information-rich protocol, such as our staircase protocol (16).

The nonlinearity of some kinetic parameters on the Eyring plots implies the Generalized Eyring relationship is a reasonable but imperfect temperature model. Under the assumption that the model structure is correct, we accurately captured the kinetics at each temperature, and the model structure stays the same for all temperatures. However, we could challenge these assumptions and suppose that the Generalized, or even Typical, Eyring relationship is true for any transition of ion channel from one conformational state to another. In this case, the Eyring formulation not matching the individual temperature parameter sets could imply that, either 1) the hERG model structure that we have assumed is incorrect (i.e., the relationship not holding is a consequence of discrepancy between the model and reality); 2) our procedure did not accurately capture the kinetic parameters at each temperature, but the fact that the parameters give excellent fits and predictions (and many parameters do follow expected trends) perhaps alleviates this concern; or 3) in reality, the energy landscape of ion channel conformations changes with the temperature, and a given transition in the model represents a different jump in conformational state (i.e., the model structure should change with temperature, which has been modeled previously (54)).

In any case, applying a simple treatment such as the Q_{10} coefficient to an imperfect model that violates the assumptions above would not automatically alleviate any mismatch. Because our temperature-specific fits can replicate both the experimental training data and the validation data very well at all temperatures, the model is a good representation of hERG kinetics. Hence, it is better to apply a rapid and reproducible procedure, as illustrated here, for generating all the parameters within a model at a new temperature, whenever possible. However, if necessary, then the Generalized Eyring relationship would be a preferable choice for predicting kinetics at a new temperature in which measurements cannot be, or have not been, taken. Although further work might show our results are more generally applicable to other channels, for now, they should be interpreted as being specific to hERG1a.

Our results have strong implications for how drug screening assays should be performed and interpreted. Because many of the drug screening platforms work only at an ambient temperature, measurements at different temperatures not only give rise to a large source of (deterministic) variation but also introduce the problem of translation of their findings to physiological temperatures. This translation is particularly problematic when an imperfect temperature model is used, such as the commonly used Q_{10} coefficient, as shown in this study. Extreme caution should be taken when using temperature-extrapolated in vitro drug screening data in in silico models for risk prediction.

Given Q_{10} coefficients cannot capture the full temperature dependence of hERG kinetics (as shown in Fig. 7) and different drugs target different kinetics, then a previous finding that there are no common sets of Q_{10} coefficients to describe the kinetics of drug block (55) is consistent with our results. In future, one could use our models to

TABLE 1 The Protocol Dependence of Q_{10} Coefficient Estimates for Each Gating Process

	Zhou et al. (4)		Vandenberg et al. (3)	
	Reported Values	Model Estimation	Reported Values	Model Estimation
Activation	6.25 ± 2.55	10.668 ± 7.482	2.1 ± 0.30	7.400 ± 4.111
Deactivation	–	2.016 ± 0.764	17 ± 0.30	3.692 ± 1.224
Inactivation	3.55 ± 0.87	3.421 ± 1.028	2.5 ± 0.53	2.750 ± 0.900
Recovery	3.65 ± 0.73	2.991 ± 0.730	2.6 ± 0.26	4.436 ± 2.763

The model estimates were derived from simulated currents using the same temperature-specific parameters (from fits at 25 and 37°C) under the different protocols performed in the two literature studies.

study some of the temperature effects observed in drug studies (11).

CONCLUSIONS

We have studied the temperature dependence of hERG kinetics using a 15-second high-information content protocol developed in Part I of this study (16). We characterized the temperature dependence by fitting a mathematical model of hERG channel kinetics to data obtained at five distinct temperatures between 25 and 37°C. We constructed between 45 and 124 cell-specific hERG models at each temperature using the 15-second calibration protocol, and our cell-specific variants of the hERG model were able to predict currents under eight independent validation protocols with high accuracy. We represented the variability in parameters using a hierarchical Bayesian model and were able to reproduce the temperature dependence observed in previous literature studies. Our models reveal that the hERG activation process has a higher temperature sensitivity than the inactivation process. The temperature dependence of the kinetic parameters we obtained takes a more complicated form than that predicted by Q_{10} coefficients or a Typical Eyring approach, although it broadly follows a Generalized Eyring relationship. Our results show that a direct fit to the 15-second protocol is the best representation of hERG kinetics at a given temperature, although predictions from the Generalized Eyring theory may be preferentially used if no such data are available.

SUPPORTING MATERIAL

Supporting Material can be found online at https://doi.org/10.1016/j.bpj.2019.07.030.

AUTHOR CONTRIBUTIONS

C.L.L., M.C., K.A.B., D.J.G., L.P., K.W., and G.R.M. designed the research. C.L.L., D.M., J.C.H., K.A.B., L.P., and K.W. carried out pilot studies and the experiments shown here. C.L.L., M.C., D.J.G., and G.R.M. designed the computational analysis. C.L.L. wrote simulation codes, performed the analysis, and generated the results figures. All authors wrote and approved the final version of the manuscript.

ACKNOWLEDGMENTS

We thank Adam Hill and Jamie Vandenberg from the Victor Chang Cardiac Research Institute (Sydney, NSW, Australia) for helpful discussions.

This work was supported by the Wellcome Trust (grants 101222/Z/13/Z and 212203/Z/18/Z); the Engineering and Physical Sciences Research Council (grants EP/G037280/1, EP/I017909/1, EP/K503769/1, and Medical Research Council (grant EP/L016044/1); and the Biotechnology and Biological Sciences Research Council (grant BB/P010008/1). C.L.L. acknowledges support from the Clarendon Scholarship Fund. C.L.L and K.A.B. acknowledge the Engineering and Physical Sciences Research Council, Medical Research Council, GlaxoSmithKline, and F. Hoffman-LaRoche for studentship support via the Oxford Systems Approaches to Biomedical Science Centre for Doctoral Training. K.A.B. also acknowledges support from the Engineering and Physical Sciences Research Council via the 2020 Science programme and an Impact Acceleration Award. M.C. and D.J.G. acknowledge support from a Biotechnology and Biological Sciences Research Council project grant. G.R.M. acknowledges support from the Wellcome Trust and Royal Society via a Sir Henry Dale Fellowship and a Wellcome Trust Senior Research Fellowship. L.P. and K.W. are employees of F. Hoffman-LaRoche, and K.W. is a shareholder. K.A.B. is an employee and shareholder of GlaxoSmithKline.

SUPPORTING CITATIONS

Reference (56) appears in the Supporting Material

REFERENCES

1. Dhaka, A., V. Viswanath, and A. Patapoutian. 2006. Trp ion channels and temperature sensation. *Annu. Rev. Neurosci.* 29:135–161.
2. Yang, F., and J. Zheng. 2014. High temperature sensitivity is intrinsic to voltage-gated potassium channels. *eLife.* 3:e03255.
3. Vandenberg, J. I., A. Varghese, …, C. L. Huang. 2006. Temperature dependence of human ether-a-go-go-related gene K+ currents. *Am. J. Physiol. Cell Physiol.* 291:C165–C175.
4. Zhou, Z., Q. Gong, …, C. T. January. 1998. Properties of HERG channels stably expressed in HEK 293 cells studied at physiological temperature. *Biophys. J.* 74:230–241.
5. Mauerhöfer, M., and C. K. Bauer. 2016. Effects of temperature on heteromeric Kv11.1a/1b and Kv11.3 channels. *Biophys. J.* 111:504–523.
6. Sanguinetti, M. C., C. Jiang, …, M. T. Keating. 1995. A mechanistic link between an inherited and an acquired cardiac arrhythmia: HERG encodes the IKr potassium channel. *Cell.* 81:299–307.
7. Jurkiewicz, N. K., and M. C. Sanguinetti. 1993. Rate-dependent prolongation of cardiac action potentials by a methanesulfonanilide class III antiarrhythmic agent. Specific block of rapidly activating delayed rectifier K+ current by dofetilide. *Circ. Res.* 72:75–83.
8. Malik, M., and A. J. Camm. 2001. Evaluation of drug-induced QT interval prolongation: implications for drug approval and labelling. *Drug Saf.* 24:323–351.
9. Friedrichs, G. S., L. Patmore, and A. Bass. 2005. Non-clinical evaluation of ventricular repolarization (ICH S7B): results of an interim survey of international pharmaceutical companies. *J. Pharmacol. Toxicol. Methods.* 52:6–11.
10. Redfern, W. S., L. Carlsson, …, T. G. Hammond. 2003. Relationships between preclinical cardiac electrophysiology, clinical QT interval prolongation and torsade de pointes for a broad range of drugs: evidence for a provisional safety margin in drug development. *Cardiovasc. Res.* 58:32–45.
11. Kirsch, G. E., E. S. Trepakova, …, A. M. Brown. 2004. Variability in the measurement of hERG potassium channel inhibition: effects of temperature and stimulus pattern. *J. Pharmacol. Toxicol. Methods.* 50:93–101.
12. Yao, J. A., X. Du, …, P. Atterson. 2005. Estimation of potency of HERG channel blockers: impact of voltage protocol and temperature. *J. Pharmacol. Toxicol. Methods.* 52:146–153.
13. Lee, W., M. J. Windley, …, A. P. Hill. 2019. Protocol-dependent differences in IC_{50} values measured in human ether-Á-go-go-related gene assays occur in a predictable way and can be used to quantify state preference of drug binding. *Mol. Pharmacol.* 95:537–550.
14. Fink, M., D. Noble, …, W. R. Giles. 2008. Contributions of HERG K+ current to repolarization of the human ventricular action potential. *Prog. Biophys. Mol. Biol.* 96:357–376.

15. Li, Z., S. Dutta, …, T. Colatsky. 2016. A temperature-dependent in silico model of the human ether-à-go-go-related (hERG) gene channel. *J. Pharmacol. Toxicol. Methods.* 81:233–239.
16. Lei, C. L., M. Clerx, …, K. Wang. 2019. Rapid characterisation of hERG potassium channel kinetics I: using an automated high-throughput system. *Biophys. J.* 117:XXX–XXX.
17. Hodgkin, A. L., and A. F. Huxley. 1952. A quantitative description of membrane current and its application to conduction and excitation in nerve. *J. Physiol.* 117:500–544.
18. Fink, M., and D. Noble. 2009. Markov models for ion channels: versatility versus identifiability and speed. *Philos. Trans. A Math. Phys. Eng. Sci.* 367:2161–2179.
19. Tsien, R. W., and D. Noble. 1969. A transition state theory approach to the kinetics of conductance changes in excitable membranes. *J. Membr. Biol.* 1:248–273.
20. Stevens, C. F. 1978. Interactions between intrinsic membrane protein and electric field. An approach to studying nerve excitability. *Biophys. J.* 22:295–306.
21. Hille, B. 2001. Ion Channels of Excitable Membranes. Sinauer Associates, Sunderland, MA.
22. Eyring, H. 1935. The activated complex in chemical reactions. *J. Chem. Phys.* 3:107–115.
23. Irvine, L. A., M. S. Jafri, and R. L. Winslow. 1999. Cardiac sodium channel Markov model with temperature dependence and recovery from inactivation. *Biophys. J.* 76:1868–1885.
24. Bhattacharyya, G. K., and Z. Soejoeti. 1981. Asymptotic normality and efficiency of modified least squares estimators in some accelerated life test models. *Sankhya Ser. B.* 43:18–39.
25. Escobar, L. A., and W. Q. Meeker. 2007. A review of accelerated test models. *Stat. Sci.* 21:552–577.
26. Redondo-Iglesias, E., P. Venet, and S. Pelissier. 2017. Eyring acceleration model for predicting calendar ageing of lithium-ion batteries. *J. Energy Storage.* 13:176–183.
27. Redondo-Iglesias, E., P. Venet, and S. Pelissier. 2018. Global model for self-discharge and capacity fade in lithium-ion batteries based on the generalized eyring relationship. *IEEE Trans. Vehicular Technol.* 67:104–113.
28. Běhrádek, J. 1930. Temperature coefficients in biology. *Biol. Rev. Camb. Philos. Soc.* 5:30–58.
29. Hodgkin, A. L., A. F. Huxley, and B. Katz. 1952. Measurement of current-voltage relations in the membrane of the giant axon of Loligo. *J. Physiol.* 116:424–448.
30. Collins, C. A., and E. Rojas. 1982. Temperature dependence of the sodium channel gating kinetics in the node of Ranvier. *Q. J. Exp. Physiol.* 67:41–55.
31. Ito, E., Y. Ikemoto, and T. Yoshioka. 2015. Thermodynamic implications of high Q 10 of thermo-TRP channels in living cells. *Biophysics (Nagoya-shi).* 11:33–38.
32. Greenstein, J. L., and R. L. Winslow. 2002. An integrative model of the cardiac ventricular myocyte incorporating local control of Ca2+ release. *Biophys. J.* 83:2918–2945.
33. Iyer, V., R. Mazhari, and R. L. Winslow. 2004. A computational model of the human left-ventricular epicardial myocyte. *Biophys. J.* 87:1507–1525.
34. Tanskanen, A. J., J. L. Greenstein, …, R. L. Winslow. 2005. The role of stochastic and modal gating of cardiac L-type Ca2+ channels on early after-depolarizations. *Biophys. J.* 88:85–95.
35. Sampson, K. J., V. Iyer, …, R. S. Kass. 2010. A computational model of Purkinje fibre single cell electrophysiology: implications for the long QT syndrome. *J. Physiol.* 588:2643–2655.
36. Mazhari, R., J. L. Greenstein, …, H. B. Nuss. 2001. Molecular interactions between two long-QT syndrome gene products, HERG and KCNE2, rationalized by in vitro and in silico analysis. *Circ. Res.* 89:33–38.
37. Clerx, M. 2018. Personalization of cellular electrophysiology models: utopia? *Comput. Cardiol.* 45:10.22489/CinC.2018.063.
38. Christé, G., O. Thériault, …, P. Chevalier. 2008. A new C-terminal hERG mutation A915fs+47X associated with symptomatic LQT2 and auditory-trigger syncope. *Heart Rhythm.* 5:1577–1586.
39. Niederer, S. A., M. Fink, …, N. P. Smith. 2009. A meta-analysis of cardiac electrophysiology computational models. *Exp. Physiol.* 94:486–495.
40. Courtemanche, M., R. J. Ramirez, and S. Nattel. 1998. Ionic mechanisms underlying human atrial action potential properties: insights from a mathematical model. *Am. J. Physiol.* 275:H301–H321.
41. ten Tusscher, K. H., D. Noble, …, A. V. Panfilov. 2004. A model for human ventricular tissue. *Am. J. Physiol. Heart Circ. Physiol.* 286:H1573–H1589.
42. O'Hara, T., L. Virág, …, Y. Rudy. 2011. Simulation of the undiseased human cardiac ventricular action potential: model formulation and experimental validation. *PLoS Comput. Biol.* 7:e1002061.
43. Paci, M., J. Hyttinen, …, S. Severi. 2013. Computational models of ventricular- and atrial-like human induced pluripotent stem cell derived cardiomyocytes. *Ann. Biomed. Eng.* 41:2334–2348.
44. Lei, C. L., K. Wang, …, L. Polonchuk. 2017. Tailoring mathematical models to stem-cell derived cardiomyocyte lines can improve predictions of drug-induced changes to their electrophysiology. *Front. Physiol.* 8:986.
45. Beattie, K. A., A. P. Hill, …, G. R. Mirams. 2018. Sinusoidal voltage protocols for rapid characterisation of ion channel kinetics. *J. Physiol.* 596:1813–1828.
46. Clerx, M., P. Collins, …, P. G. Volders. 2016. Myokit: a simple interface to cardiac cellular electrophysiology. *Prog. Biophys. Mol. Biol.* 120:100–114.
47. Hindmarsh, A. C., P. N. Brown, …, C. S. Woodward. 2005. SUNDIALS: suite of nonlinear and differential/algebraic equation solvers. *ACM Trans. Math. Softw.* 31:363–396.
48. Clerx, M., M. Robinson, …, D. J. Gavaghan. 2019. Probabilistic inference on noisy time series (PINTS). *Journal of Open Research Software.* 7:23.
49. Jones, E., T. Oliphant, …, P. Peterson. 2001. SciPy: open source scientific tools for Python. http://www.scipy.org.
50. Cooper, J., M. Scharm, and G. R. Mirams. 2016. The cardiac electrophysiology web lab. *Biophys. J.* 110:292–300.
51. Daly, A. C., M. Clerx, …, G. R. Mirams. 2018. Reproducible model development in the cardiac electrophysiology Web Lab. *Prog. Biophys. Mol. Biol.* 139:3–14.
52. Garny, A., D. P. Nickerson, …, P. J. Hunter. 2008. CellML and associated tools and techniques. *Philos. Trans. A Math. Phys. Eng. Sci.* 366:3017–3043.
53. Ranjan, R., G. Khazen, …, H. Markram. 2011. Channelpedia: an integrative and interactive database for ion channels. *Front. Neuroinform.* 5:36.
54. Di Veroli, G. Y., M. R. Davies, …, M. R. Boyett. 2013. High-throughput screening of drug-binding dynamics to HERG improves early drug safety assessment. *Am. J. Physiol. Heart Circ. Physiol.* 304:H104–H117.
55. Windley, M. J., W. Lee, …, A. P. Hill. 2018. The temperature dependence of kinetics associated with drug block of hERG channels is compound-specific and an important factor for proarrhythmic risk prediction. *Mol. Pharmacol.* 94:760–769.
56. Ku, H. H. 1966. Notes on the use of propagation of error formulas. *J. Res. Natl. Bur. Stand.* 70:263–273.

Biophysical Journal, Volume *117*

Supplemental Information

Rapid Characterization of hERG Channel Kinetics II: Temperature Dependence

Chon Lok Lei, Michael Clerx, Kylie A. Beattie, Dario Melgari, Jules C. Hancox, David J. Gavaghan, Liudmila Polonchuk, Ken Wang, and Gary R. Mirams

Contents

S1 Electrophysiology solutions 1
S2 Recording techniques 1
S3 Maximum conductance Estimation 2
S4 Temperature-dependent fits and predictions 2
S5 Main relative root mean square error (RRMSE) histograms 6
S6 Temperature dependence of inferred model parameters 10
S7 Other methods for temperature-dependent models 10
S8 Simulating literature temperature dependence 11
S9 Simulating literature Q_{10} estimation 13
S10 Mean model parameters 14
S11 Remaining RRMSE histograms 14
S12 Automated quality control 18
S13 A comparison with literature I_{Kr} models 21
References 21

S1 Electrophysiology solutions

The compositions of all the electrophysiology solutions, including both the external solutions (bath solutions) and the internal solution (equivalent to the pipette solution in manual patch clamp), are shown in Table S1. External solutions were added in the following order: first 'fill chip' solution to the measurement chip, and the suspended hERG cells, then the 'seal enhancer' solution for enhancing the seal by forming CaF crystal around the cells (note they have extra high concentration of Ca^{+}, so we need to reduce/dilute it later), followed by adding the extracellular 'reference' solution for Ca^{+} dilution. All the voltage clamp measurements were performed after adding all these external solutions.

The solutions were added sequentially to the wells, by removing half of the previous solutions from the wells each time. Therefore, the final ratios of the external (extracellular) solution are 1:1:2 — proportions of 0.25 of the 'Fill Chip' concentrations, 0.25 of the 'Seal Enhancer' concentrations, and 0.5 of the 'Reference' concentrations, as shown in the 'Final Extracellular' solution in Table S1.

**Solution**<br>**pH value (titrated with)**<br>**Osmolarity [mOsm]**		*Intracellular*<br>*pH 7.2 (KOH)*<br>*260-300*	*Fill Chip*<br>*pH 7.4 (NaOH)*<br>*300-330*	*Seal Enhancer*<br>*pH 7.4 (HCl)*<br>*290-330*	*Reference*<br>*pH 7.4 (HCl)*<br>*290-330*	*Final Extracellular*
**Chemicals**	**Source / Cat#**	**[ ] in mM**	**[ ] in mM**	**[ ] in mM**	**[ ] in mM**	**[ ] in mM**
NaCl	Merck / K38447104807	10	150	80	80	97.5
KCl	Merck / K36782536	10	4	4	4	4
KF	Acros Organics / 201352500	100	—	—	—	—
$MgCl_2$	Merck / A914133908	—	1	1	1	1
$CaCl_2$	Acros Organics/ 349615000	—	1.2	5	1	2.05
HEPES	Applichem A1069	10	10	10	10	10
Glucose	Fluka / 49159	—	5	5	5	5
NMDG	Fluka 66930	—	—	60	40	35
EGTA	Fluka / 03778	20	—	—	—	—
Sorbitol	Sigma / S1876	—	—	—	40	20

Table S1. Electrophysiology solutions for hERG assay on the Nanion SyncroPatch 384PE machine, all solutions are sterile filtered. All hERG cells were suspended in 1/3 Extracellular Fill Chip Solution + 2/3 Hanks' Balanced Salt Solution (HBSS).

S2 Recording techniques

All experiments were performed with Nanion SyncroPatch 384PE machine with software PatchControl384PE (v. 1.5.6 Build 22) and current traces data were exported using their complementary software DataControl384 (v. 1.5.0 Customer Release). The machine comes with a measurement chip consists of 364 wells, with 16 rows by 24 columns.

S3 Maximum conductance Estimation

Figure S1 shows an illustration of the estimation of maximum conductance value. The estimation is done by extrapolating the negative tail current (blue), after the first 40 mV to −120 mV step, back to the time the voltage step occurred (green vertical dashed line). The extrapolation is done by fitting a single exponential function (orange dashed line) to the tail current. The estimated value is indicated by the black cross. The absolute value is used as the normalisation constant of the current. Note that this value is only used for normalising plots and comparing between cells/temperatures (as the channel is close to fully active ($a \approx 1$) at this time point at all temperatures). The value is *not* used in the mathematical model fitting/validation for an individual well.

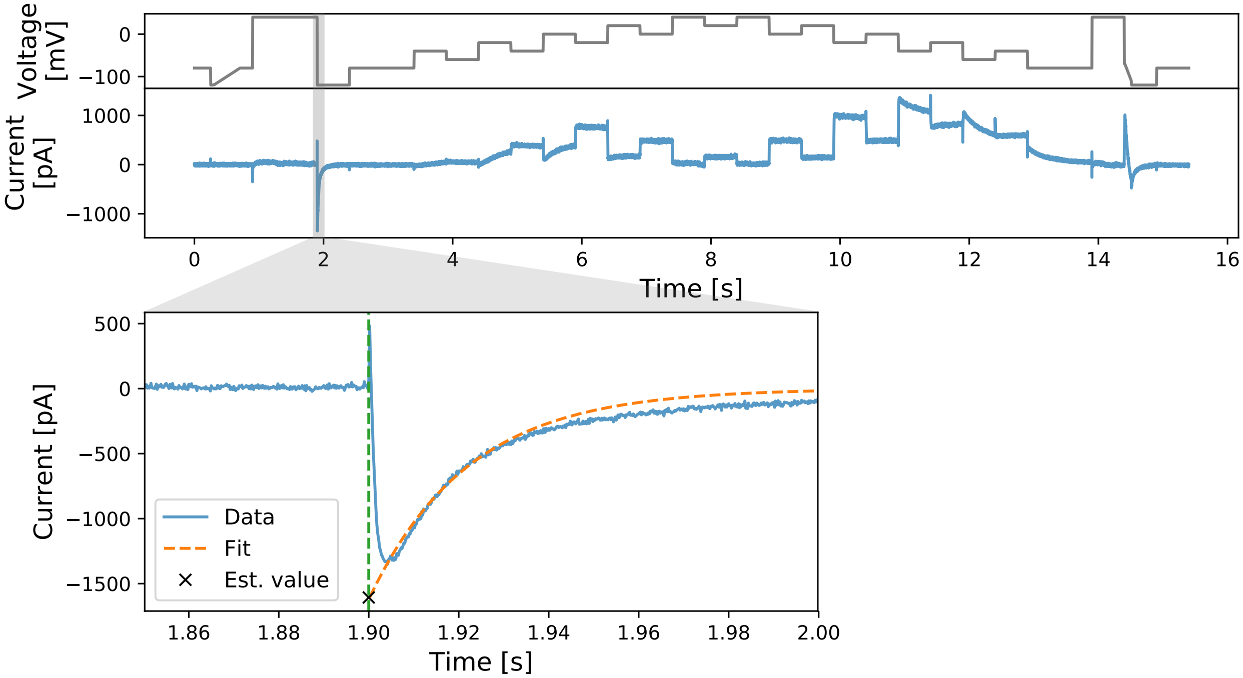

Figure S1. An illustration of the estimation of the maximum conductance value (black cross). In the magnified plot, the negative tail current (blue) after the first 40 mV to −120 mV step is extrapolated back to the time the voltage step occurred (indicated by the green vertical dashed line). The extrapolation is done by fitting a single exponential function (orange dashed line) to the tail current.

S4 Temperature-dependent fits and predictions

Figures S2, S3 and S4 show the model fitting and validation results for all recorded cells at 27, 30, and 33 °C respectively, under the 9 different protocols. From (panel A) to (panel I): the staircase protocol, the activation current-voltage (I-V) protocol, the steady-state inactivation I-V protocol, the hERG screening protocol, the DAD-like protocol, the EAD-like protocol, and the cardiac action potential-like protocol at 0.5 Hz, 1 Hz and 2 Hz, respectively. All model predictions are compared against the experimental recordings measured under the same protocols. We fitted the model to the staircase protocol (panel A) and validated against the other 8 protocols (panels B–I). To compare the variability in hERG kinetics only, currents are normalised[1] by scaling them to minimise the absolute difference between each trace and a reference trace.

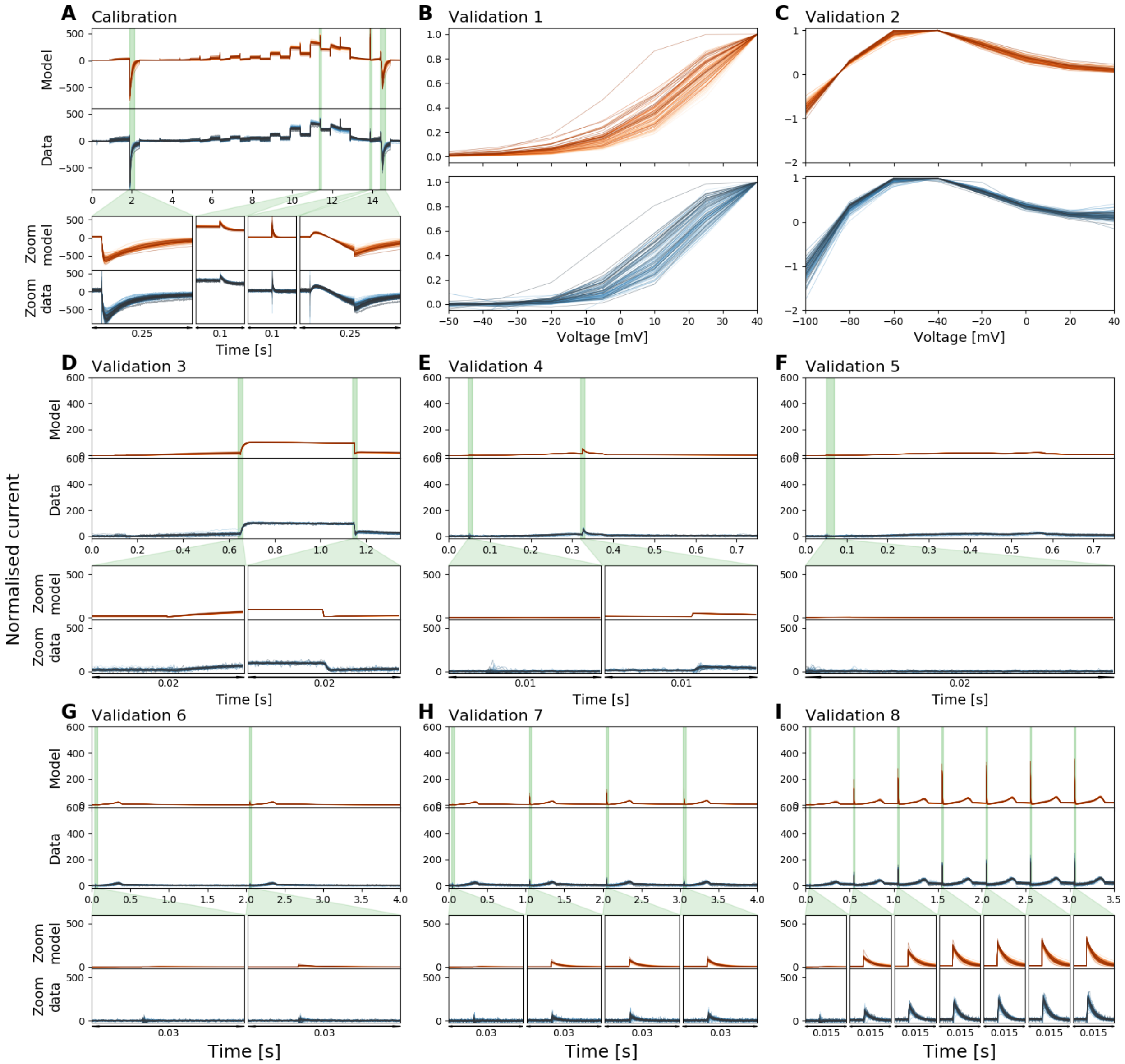

Figure S2. Normalised whole-cell patch-clamp voltage clamp recordings under 9 different protocols, and the model fitting and validation results, at 27 °C. All currents are normalised by scaling them to minimise the absolute difference between each trace and a reference trace. From **(A)** to **(I)**: The results of the staircase protocol which is used as the calibration protocol, the activation current-voltage (I-V) protocol, the steady-state inactivation I-V protocol, the hERG screening protocol, the DAD-like protocol, the EAD-like protocol, and the cardiac action potential-like protocol at 0.5 Hz, 1 Hz and 2 Hz, respectively. All the model calibration results and validation predictions are shown in the top panels (orange), and are compared against the experimental recordings shown in the bottom panels (blue). Zoomed-in of the green shaded regions are shown underneath each panel to reveal the details of the spikes, in which our models show extraordinary good predictions to the details. The normalised current for all protocols are shown except for the activation I-V protocol and the steady-state inactivation I-V protocol where the summary statistic I-V relationships are shown. Each cell is shown with a unique colour.

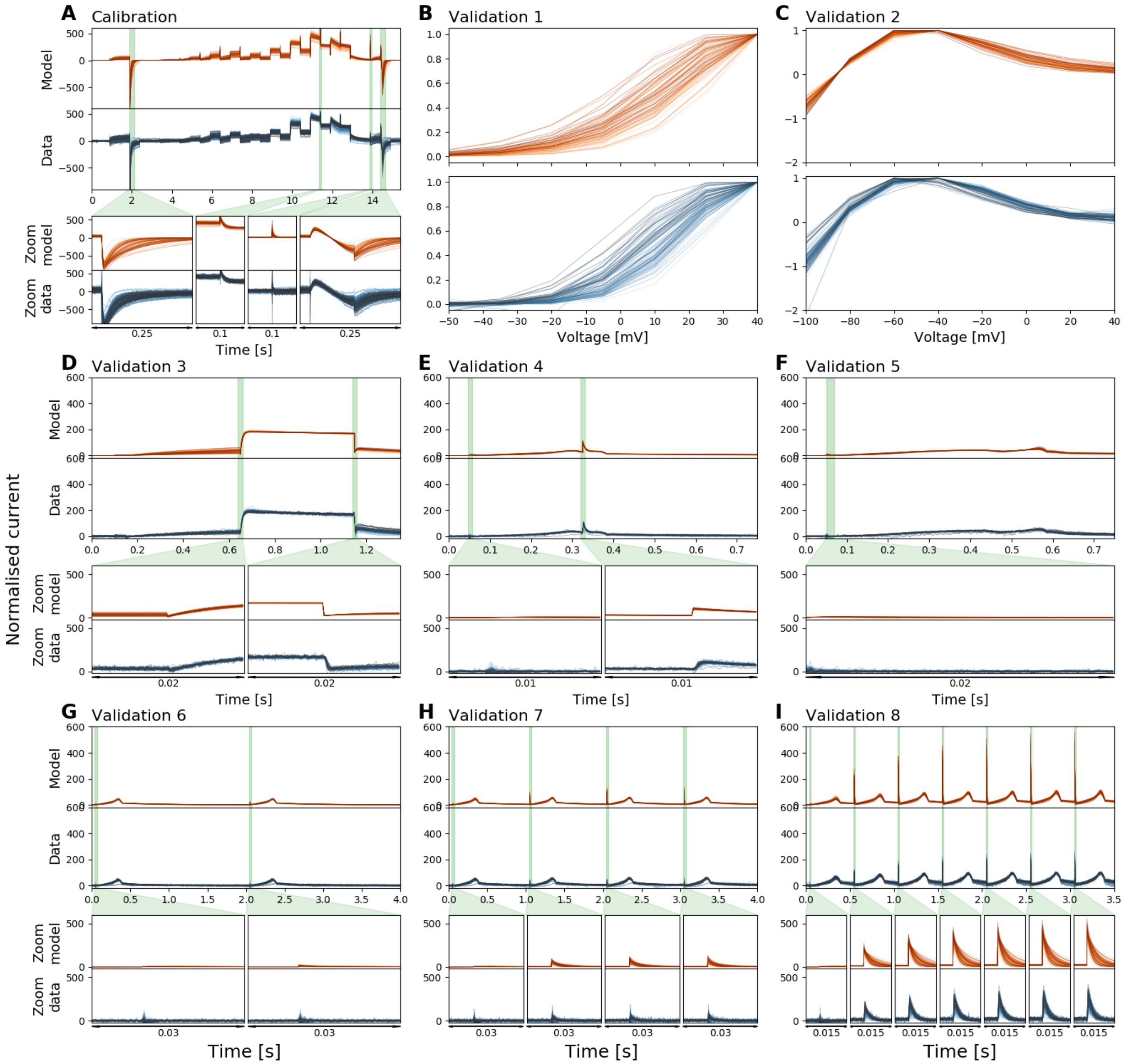

Figure S3. Normalised whole-cell patch-clamp voltage clamp recordings under 9 different protocols, and the model fitting and validation results, at 30 °C. All currents are normalised by scaling them to minimise the absolute difference between each trace and a reference trace. From **(A)** to **(I)**: The results of the staircase protocol which is used as the calibration protocol, the activation current-voltage (I-V) protocol, the steady-state inactivation I-V protocol, the hERG screening protocol, the DAD-like protocol, the EAD-like protocol, and the cardiac action potential-like protocol at 0.5 Hz, 1 Hz and 2 Hz, respectively. All the model calibration results and validation predictions are shown in the top panels (orange), and are compared against the experimental recordings shown in the bottom panels (blue). Zoomed-in of the green shaded regions are shown underneath each panel to reveal the details of the spikes, in which our models show extraordinary good predictions to the details. The normalised current for all protocols are shown except for the activation I-V protocol and the steady-state inactivation I-V protocol where the summary statistic I-V relationships are shown. Each cell is shown with a unique colour.

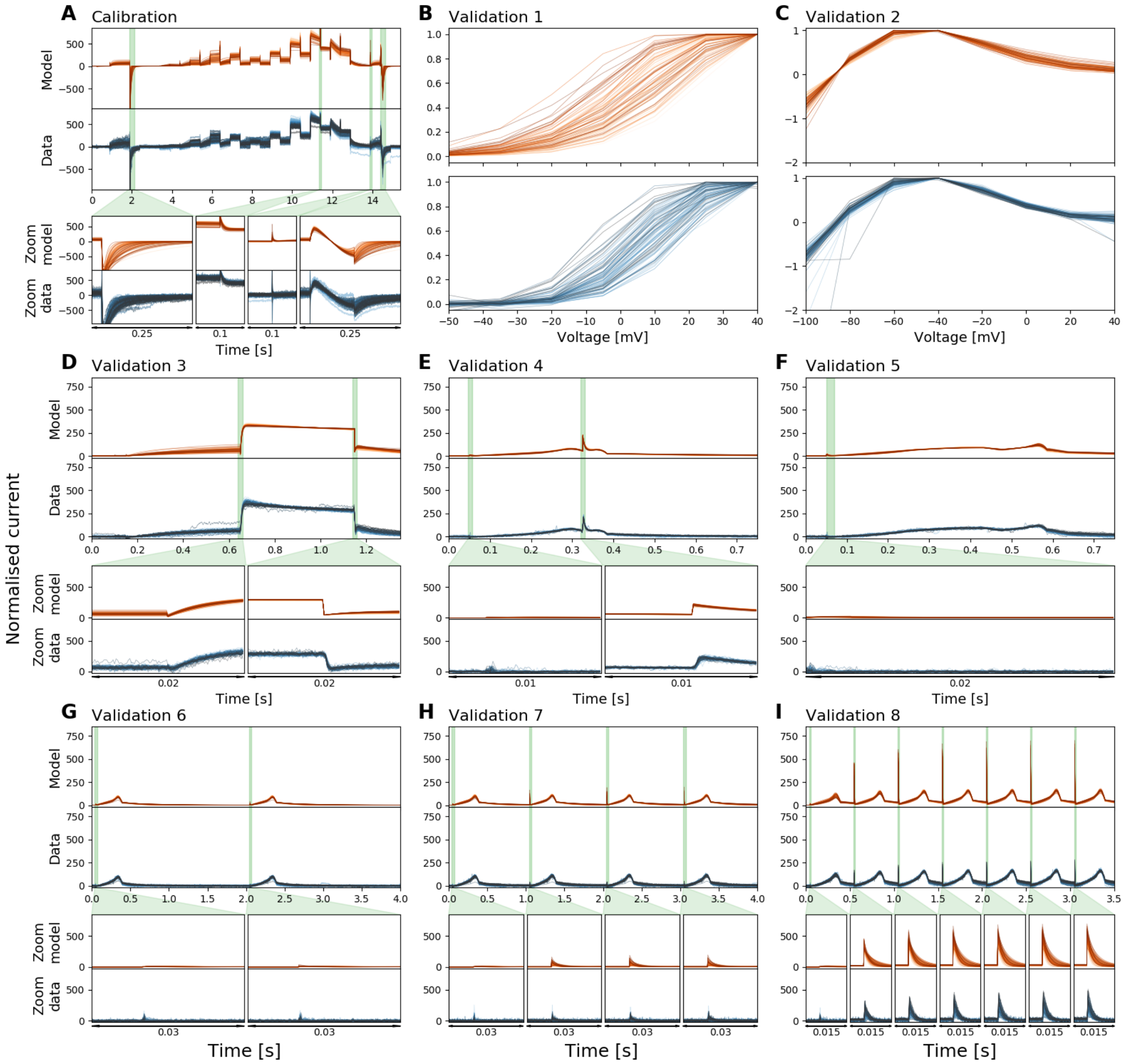

Figure S4. Normalised whole-cell patch-clamp voltage clamp recordings under 9 different protocols, and the model fitting and validation results, at 33 °C. All currents are normalised by scaling them to minimise the absolute difference between each trace and a reference trace. From **(A)** to **(I)**: The results of the staircase protocol which is used as the calibration protocol, the activation current-voltage (I-V) protocol, the steady-state inactivation I-V protocol, the hERG screening protocol, the DAD-like protocol, the EAD-like protocol, and the cardiac action potential-like protocol at 0.5 Hz, 1 Hz and 2 Hz, respectively. All the model calibration results and validation predictions are shown in the top panels (orange), and are compared against the experimental recordings shown in the bottom panels (blue). Zoomed-in of the green shaded regions are shown underneath each panel to reveal the details of the spikes, in which our models show extraordinary good predictions to the details. The normalised current for all protocols are shown except for the activation I-V protocol and the steady-state inactivation I-V protocol where the summary statistic I-V relationships are shown. Each cell is shown with a unique colour.

S5 Main relative root mean square error (RRMSE) histograms

The relative root mean square error (RRMSE, defined in Eq. 25 in the main text) analysis and the resulting histograms for 27, 30, and 33 °C are shown in Figures S5, S6, and S7 respectively. Here only the 6 main protocols are shown, for the remaining 3 protocols, see Section S11. All the results demonstrate that our hERG models are good representations of the kinetics of the cells at each corresponding temperature.

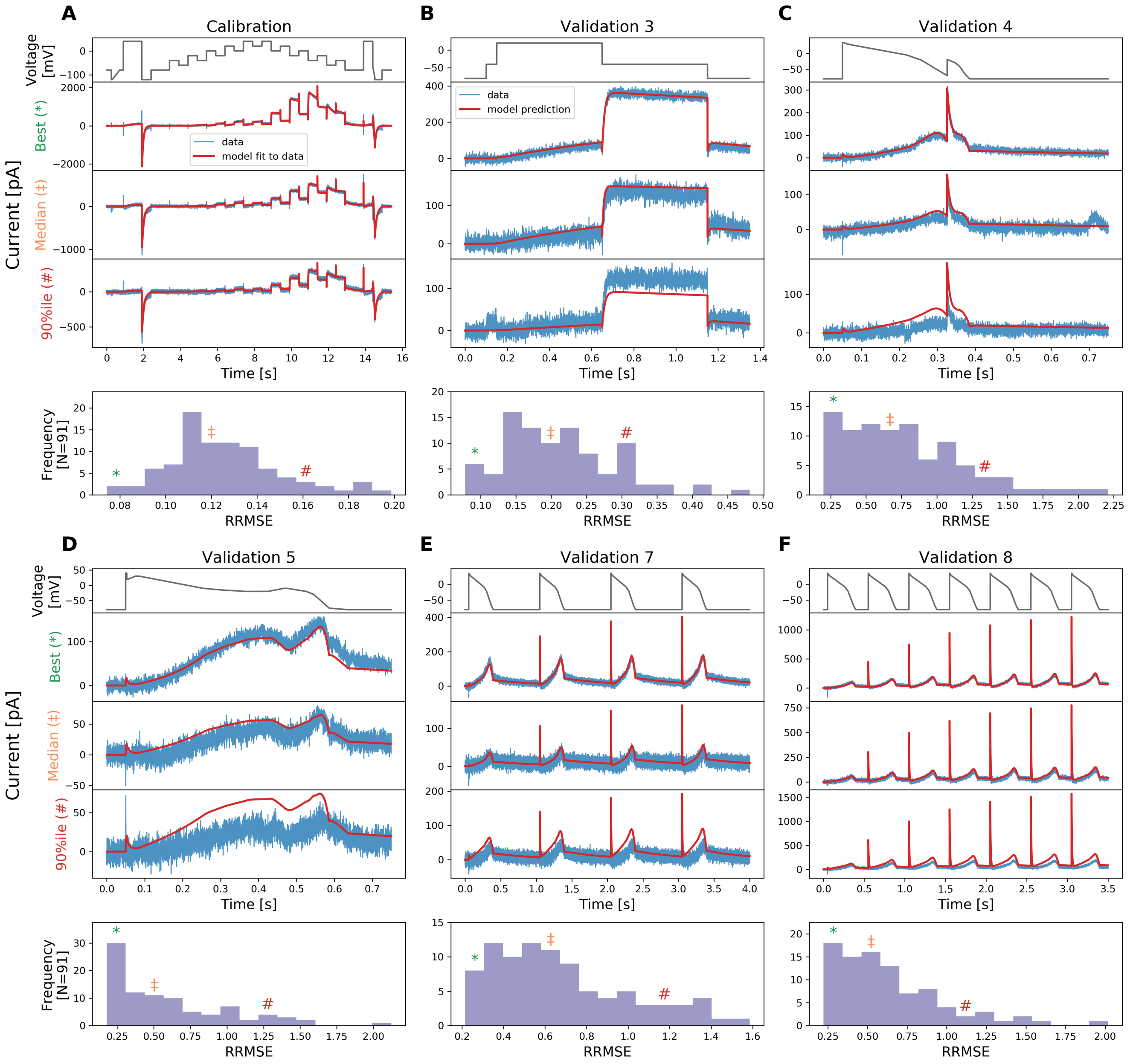

Figure S5. The relative root mean square error (RRMSE, given by Eq. 25) histograms for all cells and for 6 protocols used at 27 °C. Markers indicate the best (∗), median (‡) and 90th percentile (#) RRMSE values. The raw traces with the best, median and 90th percentile RRMSE values, for both the model (red) and data (blue), are shown in the panels above, together with the voltage protocol shown on top. Note that the currents are shown on different y-axis limits, to reveal the details of the traces.

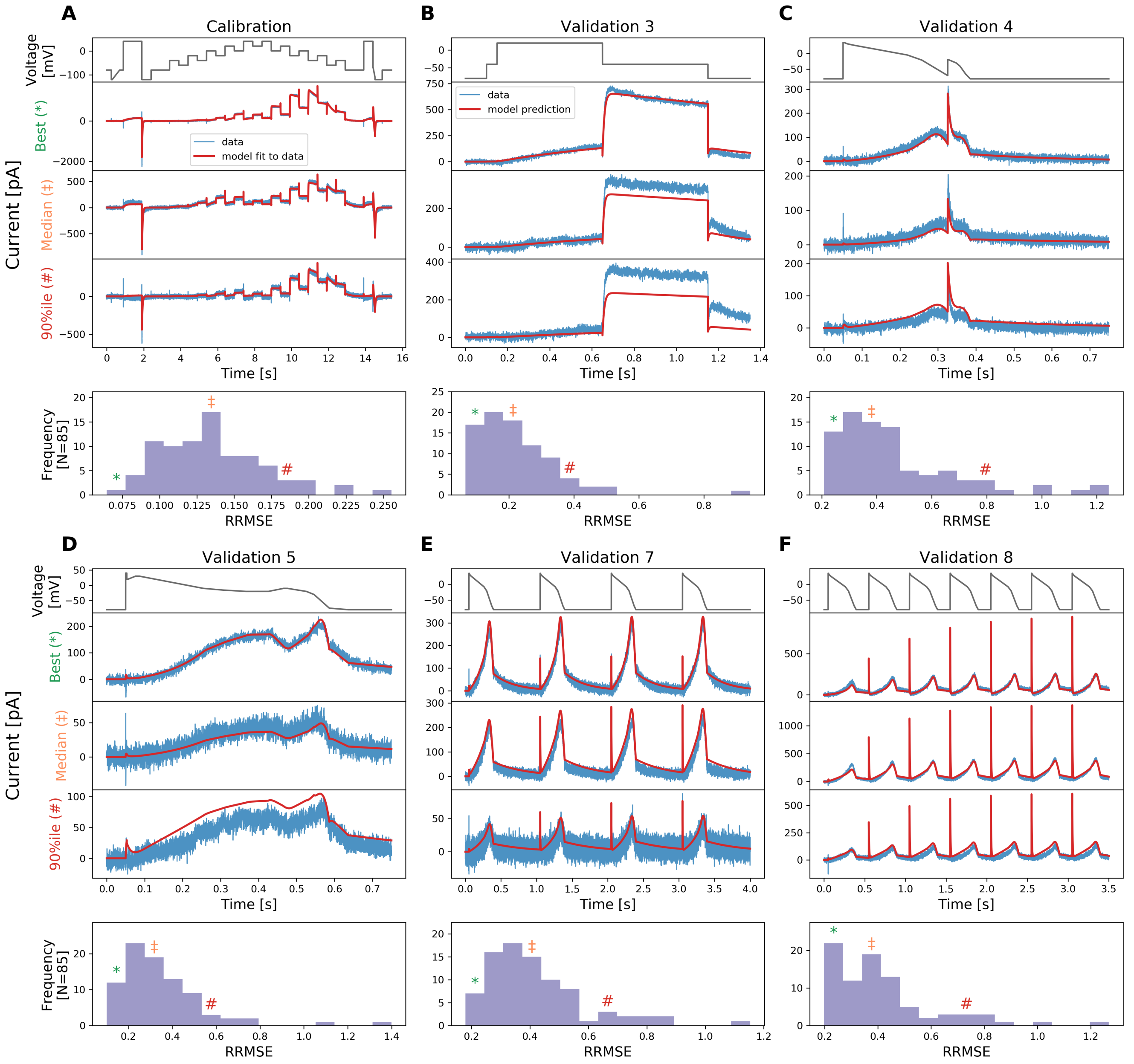

Figure S6. The relative root mean square error (RRMSE, given by Eq. 25) histograms for all cells and for 6 protocols used at 30 °C. Markers indicate the best (∗), median (‡) and 90th percentile (#) RRMSE values. The raw traces with the best, median and 90th percentile RRMSE values, for both the model (red) and data (blue), are shown in the panels above, together with the voltage protocol shown on top. Note that the currents are shown on different y-axis limits, to reveal the details of the traces.

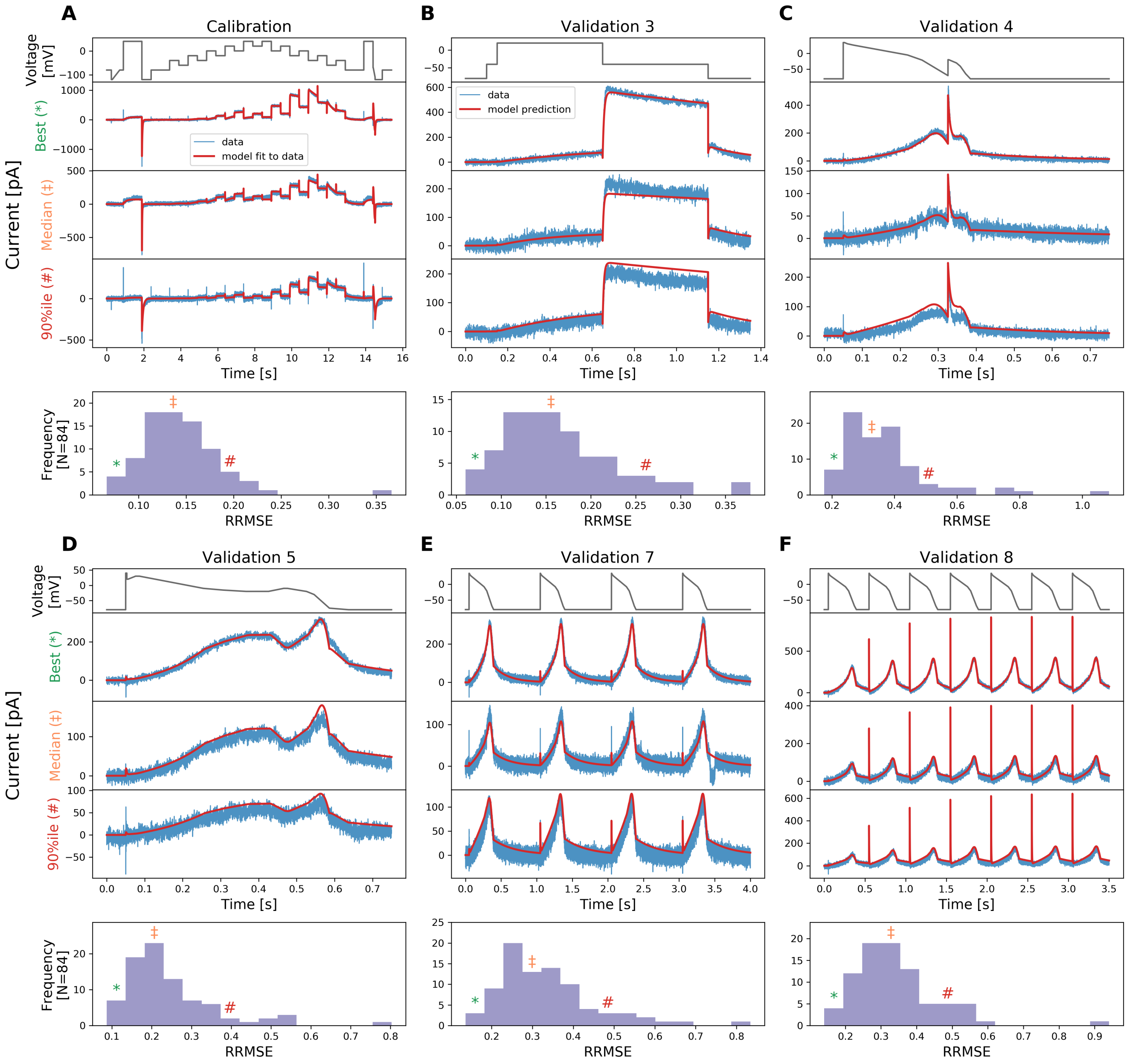

Figure S7. The relative root mean square error (RRMSE, given by Eq. 25) histograms for all cells and for 6 protocols used at 33 °C. Markers indicate the best (∗), median (‡) and 90th percentile (#) RRMSE values. The raw traces with the best, median and 90th percentile RRMSE values, for both the model (red) and data (blue), are shown in the panels above, together with the voltage protocol shown on top. Note that the currents are shown on different y-axis limits, to reveal the details of the traces.

S6 Temperature dependence of inferred model parameters

Figure S8 shows the inferred parameter values as a function of temperature. The results are shown in the Eyring plot form, where $\ln(A/T)$ and B are plotted against T^{-1}. It shows the inferred distribution of the hyperparameter mean μ (Eq. 24 in the main text) using the simplified pseudo-MwG at each temperature in a violin plot. In Figure S8, parameters $p_1, p_3, p_4, p_5, p_7, p_8$ show a linear trend as temperature increases, as predicted by Figure 1 in the main text; whereas parameters p_2, p_6 take a slightly more complicated form.

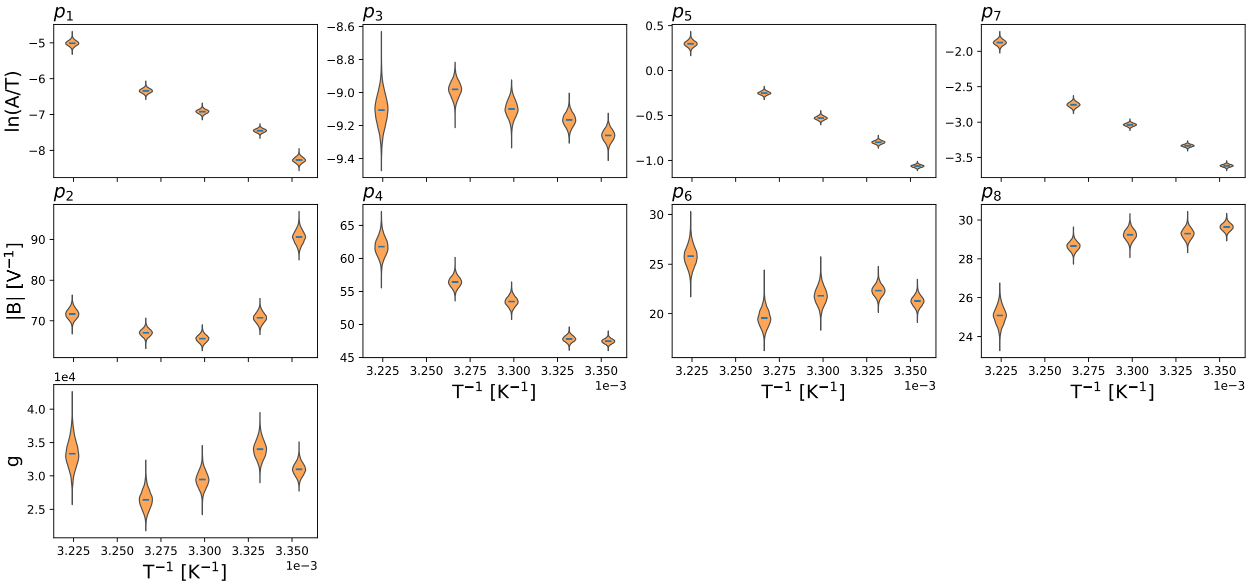

Figure S8. Model parameters plotted as a function of temperature in an Eyring plot: $\ln(A/T)$ and B are plotted against T^{-1}. Here only the inferred distribution of the hyperparameter mean μ (Eq. 24 in the main text) using the simplified pseudo-MwG at each temperature is shown. Model parameters show different degrees of temperature dependency. The conductance g does not show a prominent change as temperature increases.

S7 Other methods for temperature-dependent models

Figure S9 shows the fitted Generalised Eyring relationship and the Q_{10} equation to the inferred parameters shown in Figure S8 (orange violin plot). The obtained Generalised Eyring fits are shown as green fan charts with the first three standard deviations in green; the obtained Q_{10} fits are shown in red. The fitted parameters for the Generalised Eyring equation (Eq. 4 in the main text) and the Q_{10} equation (Eq. 9 in the main text) are given in the bottom right tables, one set for each k_i, $i = 1,2,3,4$.

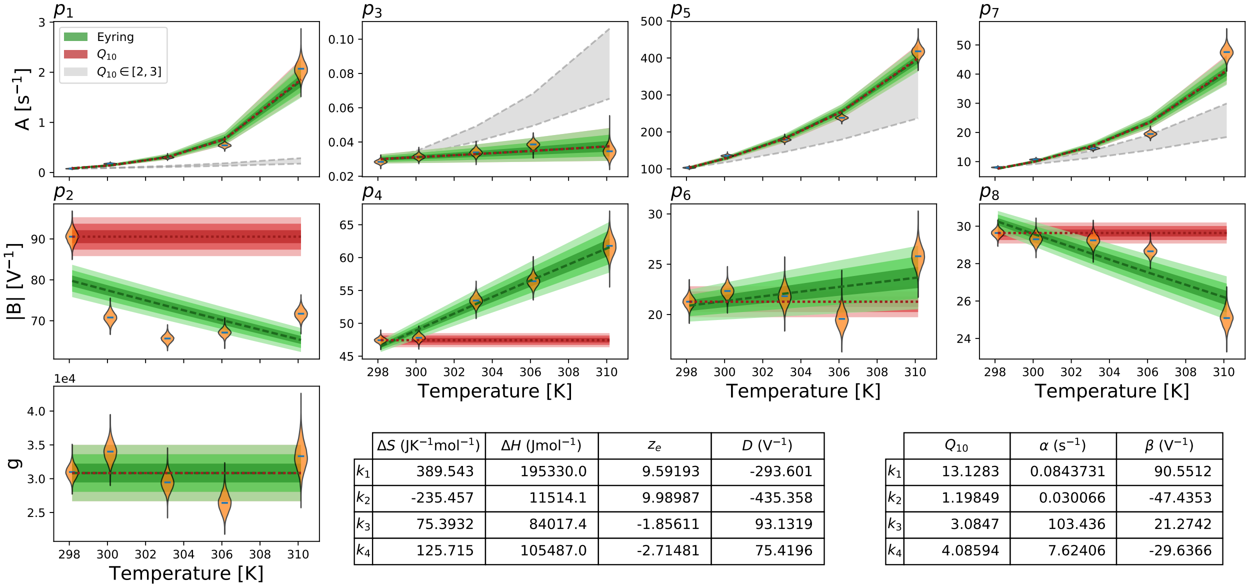

	ΔS (JK^{-1}mol^{-1})	ΔH (Jmol^{-1})	z_e	D (V^{-1})
k_1	389.543	195330.0	9.59193	-293.601
k_2	-235.457	11514.1	9.98987	-435.358
k_3	75.3932	84017.4	-1.85611	93.1319
k_4	125.715	105487.0	-2.71481	75.4196

	Q_{10}	α (s^{-1})	β (V^{-1})
k_1	13.1283	0.0843731	90.5512
k_2	1.19849	0.030066	-47.4353
k_3	3.0847	103.436	21.2742
k_4	4.08594	7.62406	-29.6366

Figure S9. Fitting of Generalised Eyring equation and Q_{10} equation to the mean distribution μ inferred using the simplified psuedo-MwG (orange violin plot). The obtained Generalised Eyring fits are shown as green fan charts with the first three standard deviations in green; the obtained Q_{10} fits are shown in red. The fitted parameters for the Generalised Eyring and Q_{10} equations are shown in the bottom right tables, one set for each k_i, $i = 1,2,3,4$. For comparison to typical Q_{10} values in literature, where Q_{10} values are usually assumed to be around 2 to 3, we show the parameters prediction using $Q_{10} \in [2,3]$ as the grey shaded region.

S8 Simulating literature temperature dependence

Here we describe the procedure for reproducing the literature results of Vandenberg *et al.*[2] (Figure 6) through model simulations. These authors estimated the 'open probability' through experimental measurements. We use our directly fitted hierarchical Bayesian models at 25 °C and 33 °C for simulation, and compare to their reported temperature induced changes with measurements performed at 22 °C and 32 °C. The results are shown in Figure 10 and are discussed in the Discussion section in the main text.

First, we simulate the voltage dependence of activation using the 'isochronal tail current protocol'[2]: a holding potential of −80 mV, followed by 30 s depolarising pulses to voltages from −80 to 40 mV, then 500 ms steps to −120 mV. The tail currents during the −120 mV steps are recorded to construct the voltage dependence of activation curves shown in Figure S10. Our results here are compared to Figure 3C in Vandenberg *et al.*[2].

Second, we simulate the steady-state inactivation using the 'double-pulse protocol'[2]: a holding potential of −80 mV, followed by 1 s depolarising pulses to voltage 40 mV, then 500 ms steps to voltages in the range −120 to 40 mV. The peak amplitude of the tail current is corrected using the method as described in Section S3, the same method used in Vandenberg *et al.*[2]. These corrected current values are then converted to conductances by dividing by the electrochemical driving force $(V - E_K)$. These are described as the steady-state inactivation curves[2] and are shown in Figure S11. Our results here are compared to Figure 5D in Vandenberg *et al.*[2].

The product of the voltage dependence of activation (Figure S10) and the steady-state inactivation (Figure S11) gives the 'open probability' defined in Vandenberg *et al.*[2] (Figure 6), which is shown in Figure 10 in the main text. Strictly speaking, this 'open probability' is *not* the open probability of the model $(O = a \cdot r)$, but an approximate of O from the simulation results following simulation of the experimental protocols and a repeat of the analyses in Vandenberg *et al.*[2] based on the simulated currents.

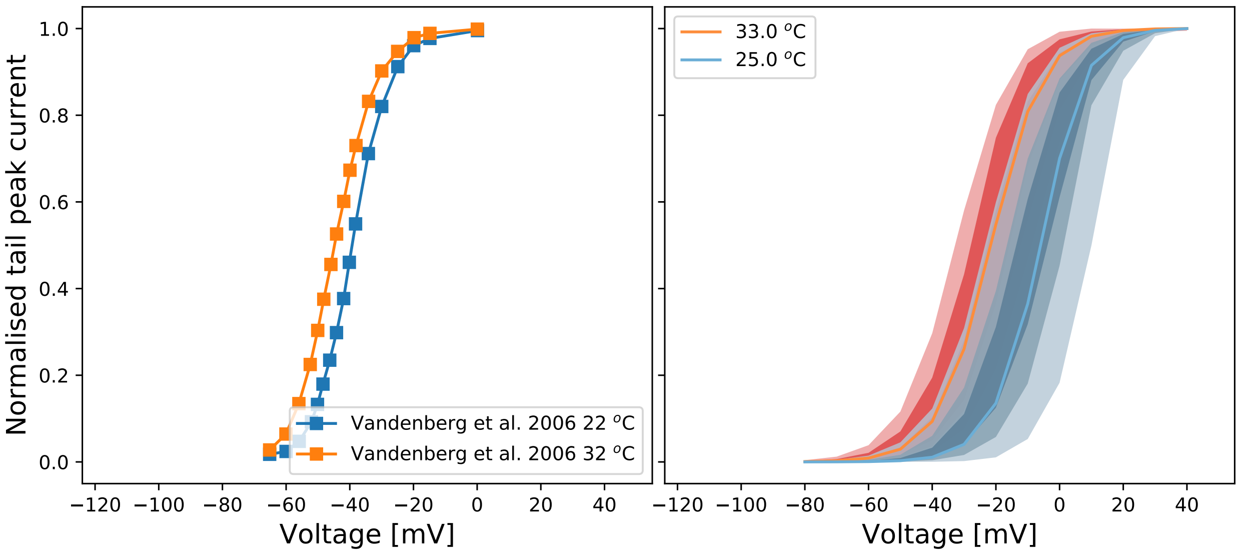

Figure S10. Voltage dependence of activation defined in Vandenberg *et al.*[2] (Figure 3C). **Left:** Data reproduced from Vandenberg *et al.*[2] (Figure 3C). **Right:** The fan charts show the the 90th, 60th and 30th percentiles of the hierarchical Bayesian model simulations, representing the *experiment-experiment* variability. Orange/red represents 32 °C to 33 °C, and blue represents 22 °C to 25 °C.

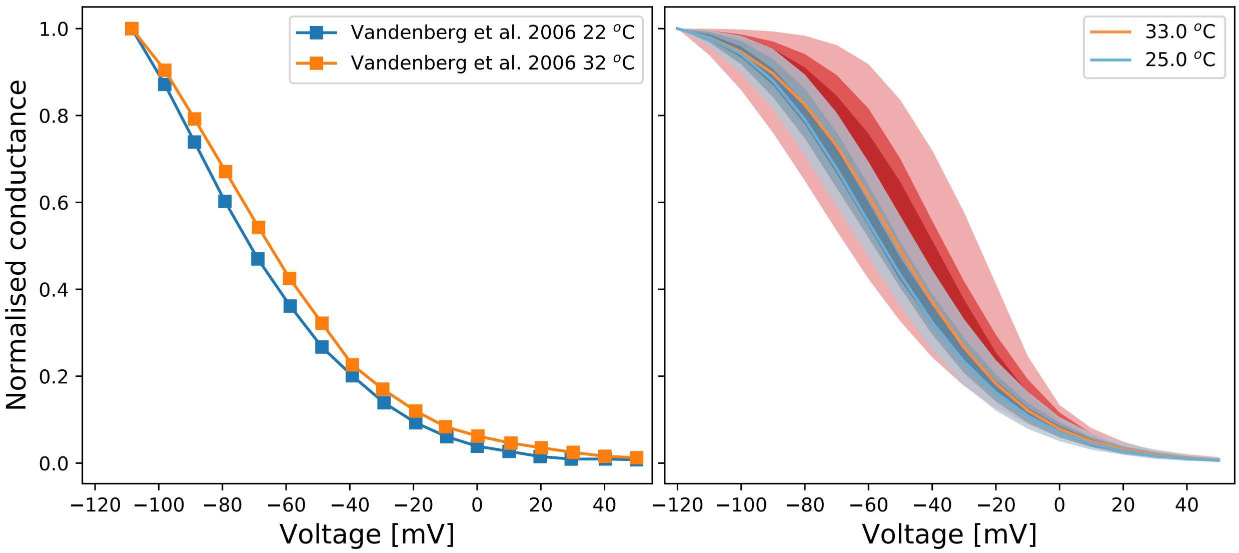

Figure S11. Steady-state inactivation defined in Vandenberg *et al.*[2] (Figure 5D). **Left:** Data reproduced from Vandenberg *et al.*[2] (Figure 5D). **Right:** The fan charts show the the 90th, 60th and 30th percentiles of the hierarchical Bayesian model simulations, representing the *experiment-experiment* variability. Orange/red represents 32 °C to 33 °C, and blue represents 22 °C to 25 °C.

We also compare the temperature effect under action potential clamps. In Figure S12, we plot experimental data from our high-throughput measurements on the left, and compare with the simulations under the protocol shown in Figure 1B of Vandenberg *et al.*[2]. These figures show similar temperature effects due to increasing temperature from 25 °C (blue) to 33 °C (red). The simulation on the right is comparable to that in Figure 1B of Vandenberg *et al.*[2]. We are able to reproduce the results that are broadly consistent with Vandenberg *et al.*[2].

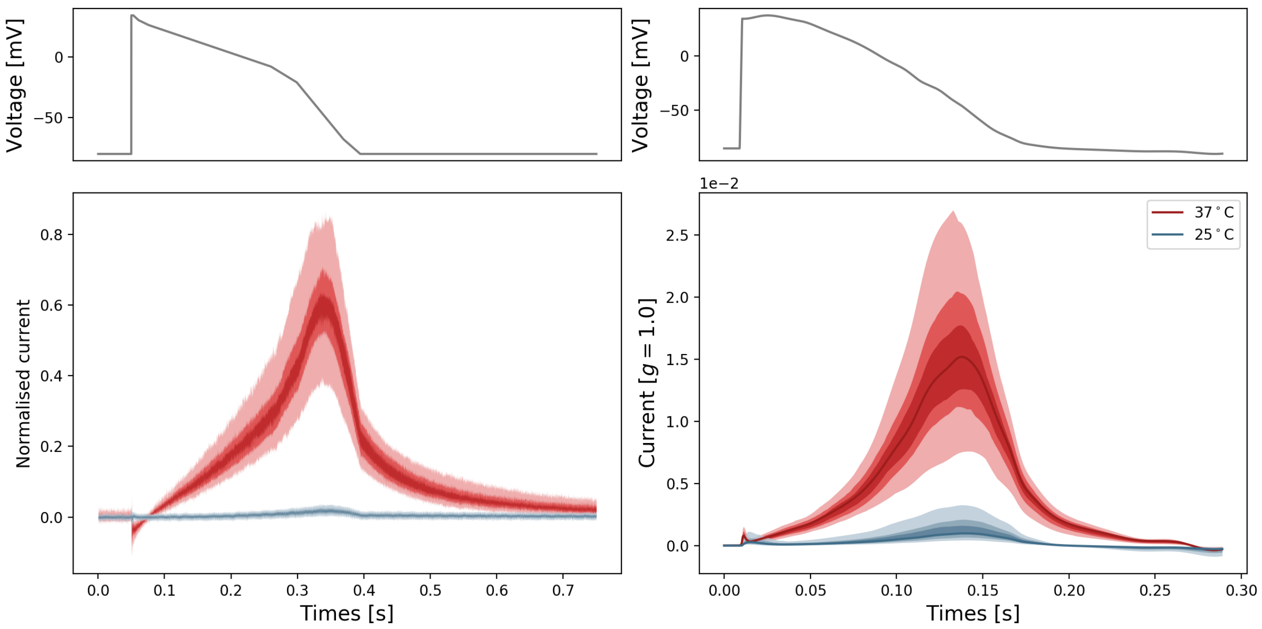

Figure S12. Action potential clamp (left) data from our high-throughput measurements and (Right) simulations under the protocol in Vandenberg *et al.*[2] (Figure 1B). The fan charts show the the 30th, 60th and 90th percentiles of the data and the hierarchical Bayesian model simulations, representing the *experiment-experiment* variability. Orange/red represents 32 °C to 33 °C, and blue represents 22 °C to 25 °C.

S9 Simulating literature Q_{10} estimation

Here we describe the procedure for reproducing the Q_{10} coefficients reported by Zhou *et al.*[3] and Vandenberg *et al.*[2] through model simulations. We use our directly fitted hierarchical Bayesian models at 25 °C and 37 °C to compute Q_{10} coefficients by simulating the experimental procedure that was used to derived the literature Q_{10} coefficients. The obtained Q_{10} are shown in Table 1 and are discussed in the Discussion section in the main text.

Q_{10} coefficients in Zhou *et al.*[3]

We simulate a depolarising step from −80 mV (holding potential) to 0 mV for 5 s which followed by a repolarising step to −50 mV for 5 s. The time constant, fitted with a single exponential, of the current during 0 mV is called the activation time constant. The fitted time constant of the tail current during the −50 mV step is called the deactivation time constant.

We then simulate the ‘three-pulse protocol’[3]: a holding potential of −80 mV, a 200 ms depolarising step to 60 mV followed by a repolarising step to −100 mV for 2 ms, then a step to voltage 0 mV for 200 ms. The fitted time constant of the current during 0 mV is called the inactivation time constant. We finally simulate the two-pulse protocol[3]: a holding potential of −80 mV, followed by a 200 ms depolarising step to 60 mV before a repolarising step to −50 mV for 200 ms. The fitted time constant of the tail current rising phase during −50 mV is called the recovery (from inactivation) time constant.

Q_{10} coefficients in Vandenberg *et al.*[2]

We simulate the ‘envelope of tails protocol’[2]: a holding potential of −80 mV, followed by a depolarising step to 0 mV for variable durations before stepping voltage to −50 mV for 500 ms. The peak amplitudes of the tail currents during the −50 mV steps are plotted against the duration of the 0 mV step. The time constant, fitted with a single exponential function, of the peak current-step duration curve is called the activation time constant.

We then simulate the 'triple pulse protocol'[2]: a holding potential of −80 mV, a 1 s depolarising step to 40 mV followed by a repolarising step to −80 mV for 20 ms, then a step to voltage 40 mV for 100 ms, and finally a step to voltage −120 mV for 200 ms. The fitted time constant of the current during the second 40 mV is called the inactivation time constant. Two time constants τ_1, τ_2 are obtained by fitting a 'double exponential' function

$$f_{\text{double exponential}}(t) = A\exp(-t/\tau_1) - B\exp(-t/\tau_2)\,, \tag{S1}$$

with constants A, B, to the tail current during −120 mV. The time constant corresponding to the initial increase in inward current is called the recovery (from inactivation) time constant; the time constant of the decrease in inward current is called the deactivation time constant.

Computing mean and standard deviation of Q_{10} coefficients

We compare the results using mean and standard deviation, such that we can take the variability of experiments into account. Instead of the time constant, we use its inverse, the rate constant κ, for further calculation. For each type of rate constant (activation, deactivation, inactivation, and recovery), we compute the mean $\hat{\kappa}$ and standard deviation $\delta\kappa$ using all the simulated results for each temperature.

Since Q_{10} is given by

$$Q_{10} = \left(\frac{\kappa_1}{\kappa_2}\right)^{10°\text{C}/(T_2-T_1)}, \tag{S2}$$

where κ_1, κ_2 are rate constants at temperature $T_1 = 25\,°\text{C}$, $T_2 = 37\,°\text{C}$ respectively. We assume a linear error and independent variables, and denote the exponent $10°\text{C}/(T_2 - T_1)$ as ΔT, the standard deviation of a variable x as δx, and its mean estimator as $\hat{x}$. Then we apply the propagation of error equation[4] which gives

$$\delta Q_{10} = \left\{ \delta\kappa_1^2 \left(\frac{\partial Q_{10}}{\partial \kappa_1}\bigg|_{\hat{Q}_{10}}\right)^2 + \delta\kappa_2^2 \left(\frac{\partial Q_{10}}{\partial \kappa_2}\bigg|_{\hat{Q}_{10}}\right)^2 + \delta T_1^2 \left(\frac{\partial Q_{10}}{\partial T_1}\bigg|_{\hat{Q}_{10}}\right)^2 + \delta T_2^2 \left(\frac{\partial Q_{10}}{\partial T_2}\bigg|_{\hat{Q}_{10}}\right)^2 \right\}^{-1/2} \tag{S3}$$

$$= \left\{ \left(\delta\kappa_1 \frac{\hat{\Delta T}}{\hat{\kappa}_1}\hat{Q}_{10}\right)^2 + \left(\delta\kappa_2 \frac{\hat{\Delta T}}{\hat{\kappa}_2}\hat{Q}_{10}\right)^2 + \left(\delta T_1 \hat{Q}_{10} \ln\frac{\hat{\kappa}_1}{\hat{\kappa}_2}\frac{\hat{\Delta T}^2}{10°\text{C}}\right)^2 + \left(\delta T_2 \hat{Q}_{10} \ln\frac{\hat{\kappa}_1}{\hat{\kappa}_2}\frac{\hat{\Delta T}^2}{10°\text{C}}\right)^2 \right\}^{-1/2}, \tag{S4}$$

where we assume $\delta T \approx 1\,°\text{C}$.

Using the mean values $\hat{\kappa}_1, \hat{\kappa}_2$ and Eq. S4, we compute an estimation of the mean and standard deviation of Q_{10} for each type of rate constant, for Zhou *et al.*[3] and Vandenberg *et al.*[2]. The results are shown in the "Model estimation" columns in Table 1 in the main text.

Since Zhou *et al.*[3] reported only the time constants of each type with mean and standard error of mean $\sigma_{\bar{x}}$ at two temperatures, we first estimate the mean and standard deviation for rate constants. We approximate the standard deviation with $\approx \sqrt{n}\sigma_{\bar{x}}$, where n is the number of cells reported for each time constant. Then we apply Eq. S4 to propagate the errors in rate constants at two temperatures to estimate the error in their Q_{10}coefficients. Vandenberg *et al.*[2] reported the Q_{10} coefficient with mean and standard error of mean for each gating process, we convert the standard error of mean to standard deviation for comparison. The results are shown in the "Reported values" columns in Table 1.

S10 Mean model parameters

Table S2 shows the mean values of the model parameters μ (in Eq. 24 in the main text) for all temperatures. Table S3 shows the 95th percentile, or 95% credible intervals, of the samples of μ for all temperatures.

S11 Remaining RRMSE histograms

Here we include the relative root mean square error (RRMSE, given by Eq. 25 in the main text) histograms for the remaining validation protocols 1, 2, and 6 that are not included in Section S5 and in the main text due to the space limit. For 27, 30, 33, and 37 °C see Figure S13, S14, S15, and S16 respectively.

	g_{Kr} $[pS]$	p_1 $[s^{-1}]$	p_2 $[V^{-1}]$	p_3 $[s^{-1}]$	p_4 $[V^{-1}]$	p_5 $[s^{-1}]$	p_6 $[V^{-1}]$	p_7 $[s^{-1}]$	p_8 $[V^{-1}]$
$T = 25°C$	3.1e+4	7.65e-2	9.05e+1	2.84e-2	4.74e+1	1.03e+2	2.13e+1	8.01e+0	2.96e+1
$T = 27°C$	3.4e+4	1.75e-1	7.08e+1	3.14e-2	4.78e+1	1.35e+2	2.23e+1	1.07e+1	2.93e+1
$T = 30°C$	2.94e+4	3.e-1	6.57e+1	3.39e-2	5.35e+1	1.79e+2	2.18e+1	1.45e+1	2.92e+1
$T = 33°C$	2.64e+4	5.40e-1	6.71e+1	3.85e-2	5.64e+1	2.38e+2	1.96e+1	1.95e+1	2.87e+1
$T = 37°C$	3.33e+4	2.07e+0	7.17e+1	3.44e-2	6.18e+1	4.18e+2	2.58e+1	4.75e+1	2.51e+1

Table S2. The mean values of the model parameters μ (in Eq. 24 in the main text) for all temperatures.

	g_{Kr} $[pS]$	p_1 $[s^{-1}]$	p_2 $[V^{-1}]$	p_3 $[s^{-1}]$	p_4 $[V^{-1}]$	p_5 $[s^{-1}]$	p_6 $[V^{-1}]$	p_7 $[s^{-1}]$	p_8 $[V^{-1}]$
$T = 25°C$	2.91e+4	6.56e-2	8.74e+1	2.65e-2	4.67e+1	1.00e+2	2.03e+1	7.73e+0	2.93e+1
	3.29e+4	8.92e-2	9.37e+1	3.05e-2	4.82e+1	1.06e+2	2.23e+1	8.29e+0	3.00e+1
$T = 27°C$	3.13e+4	1.58e-1	6.84e+1	2.90e-2	4.69e+1	1.30e+2	2.12e+1	1.03e+1	2.88e+1
	3.68e+4	1.93e-1	7.33e+1	3.4e-2	4.87e+1	1.40e+2	2.35e+1	1.11e+1	2.98e+1
$T = 30°C$	2.71e+4	2.68e-1	6.39e+1	3.05e-2	5.2e+1	1.71e+2	2.01e+1	1.39e+1	2.87e+1
	3.2e+4	3.36e-1	6.75e+1	3.76e-2	5.49e+1	1.86e+2	2.36e+1	1.52e+1	2.98e+1
$T = 33°C$	2.38e+4	4.77e-1	6.53e+1	3.5e-2	5.49e+1	2.29e+2	1.79e+1	1.81e+1	2.82e+1
	2.92e+4	6.12e-1	6.89e+1	4.24e-2	5.80e+1	2.48e+2	2.14e+1	2.09e+1	2.91e+1
$T = 37°C$	2.94e+4	1.78e+0	6.93e+1	2.84e-2	5.90e+1	3.91e+2	2.38e+1	4.4e+1	2.42e+1
	3.77e+4	2.39e+0	7.42e+1	4.18e-2	6.46e+1	4.46e+2	2.79e+1	5.13e+1	2.6e+1

Table S3. The 95th percentile of the samples, or 95% credible intervals, of the model parameters μ (in Eq. 24 in the main text) for all temperatures.

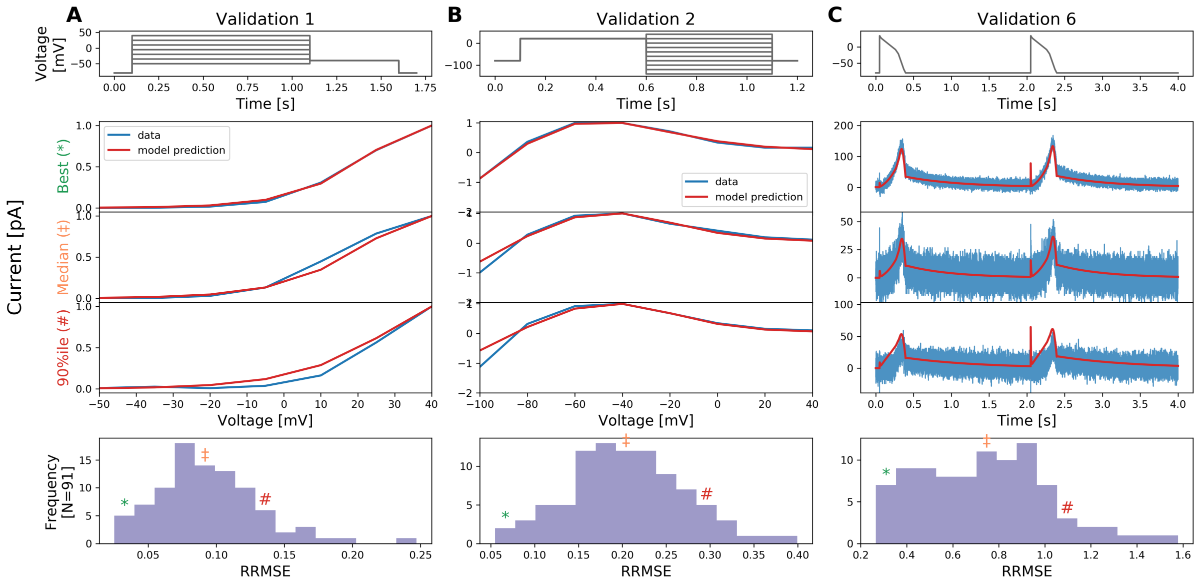

Figure S13. The relative root mean square error (RRMSE, given by Eq. 25 in the main text) histograms for all cells and for validation protocols 1, 2, and 6, at 27 °C. Markers indicate the best (∗), median (‡) and 90th percentile (#) RRMSE values. For each protocol, the raw traces with the best, median and 90th percentile RRMSE values, for both the model (red) and data (blue) are shown, with the voltage clamp above. Note that the currents are shown on different scales, to reveal the details of the traces.

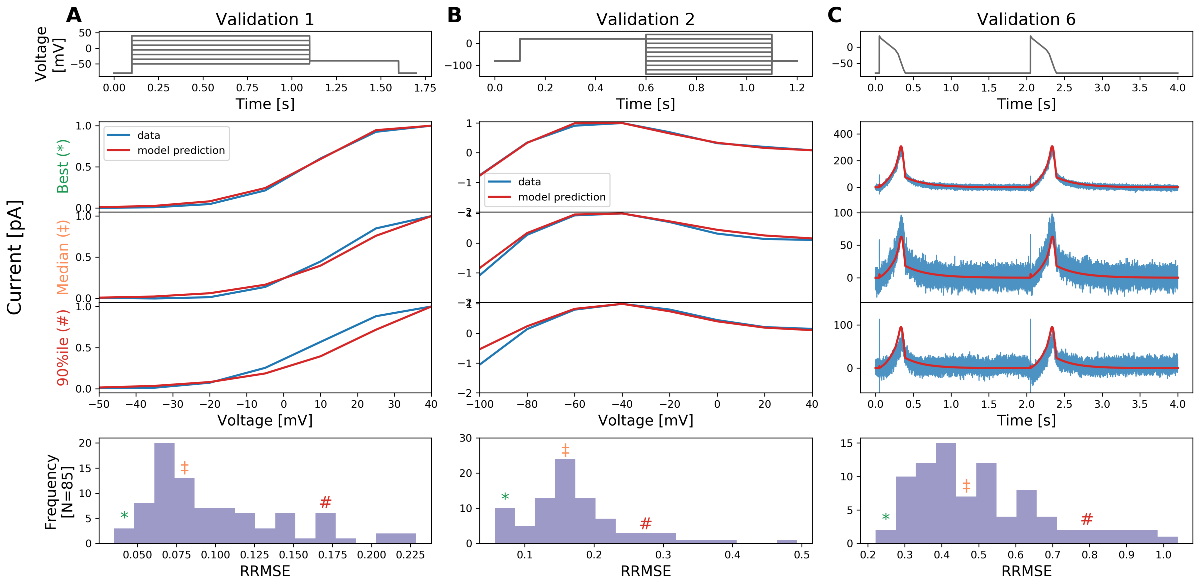

Figure S14. The relative root mean square error (RRMSE, given by Eq. 25 in the main text) histograms for all cells and for validation protocols 1, 2, and 6, at 30 °C. Markers indicate the best (∗), median (‡) and 90th percentile (#) RRMSE values. For each protocol, the raw traces with the best, median and 90th percentile RRMSE values, for both the model (red) and data (blue) are shown, with the voltage clamp above. Note that the currents are shown on different scales, to reveal the details of the traces.

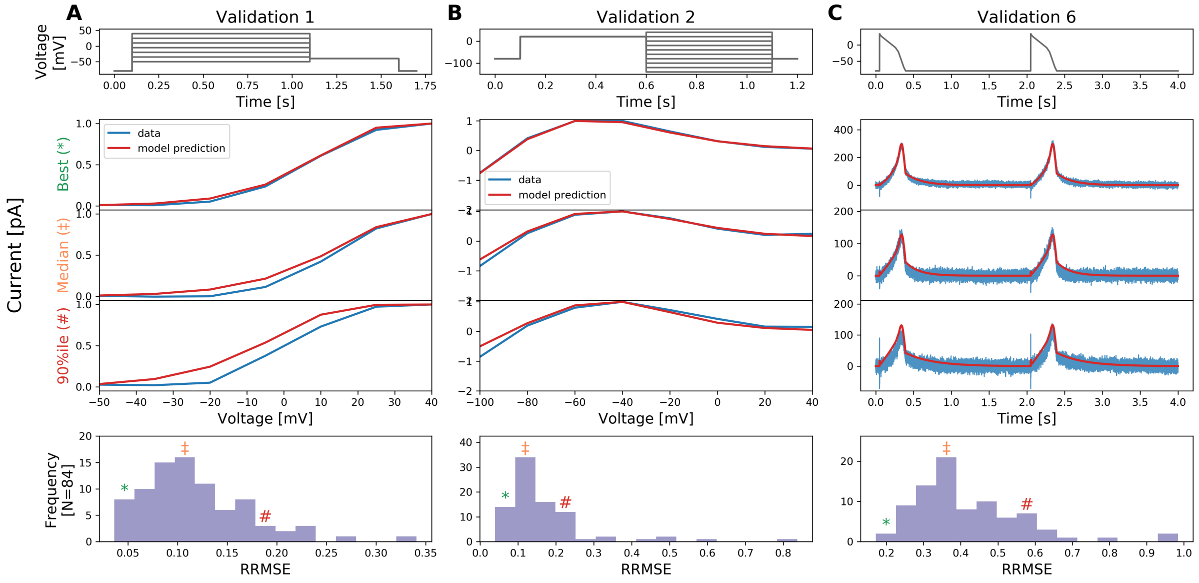

Figure S15. The relative root mean square error (RRMSE, given by Eq. 25 in the main text) histograms for all cells and for validation protocols 1, 2, and 6, at 33 °C. Markers indicate the best (∗), median (‡) and 90th percentile (#) RRMSE values. For each protocol, the raw traces with the best, median and 90th percentile RRMSE values, for both the model (red) and data (blue) are shown, with the voltage clamp above. Note that the currents are shown on different scales, to reveal the details of the traces.

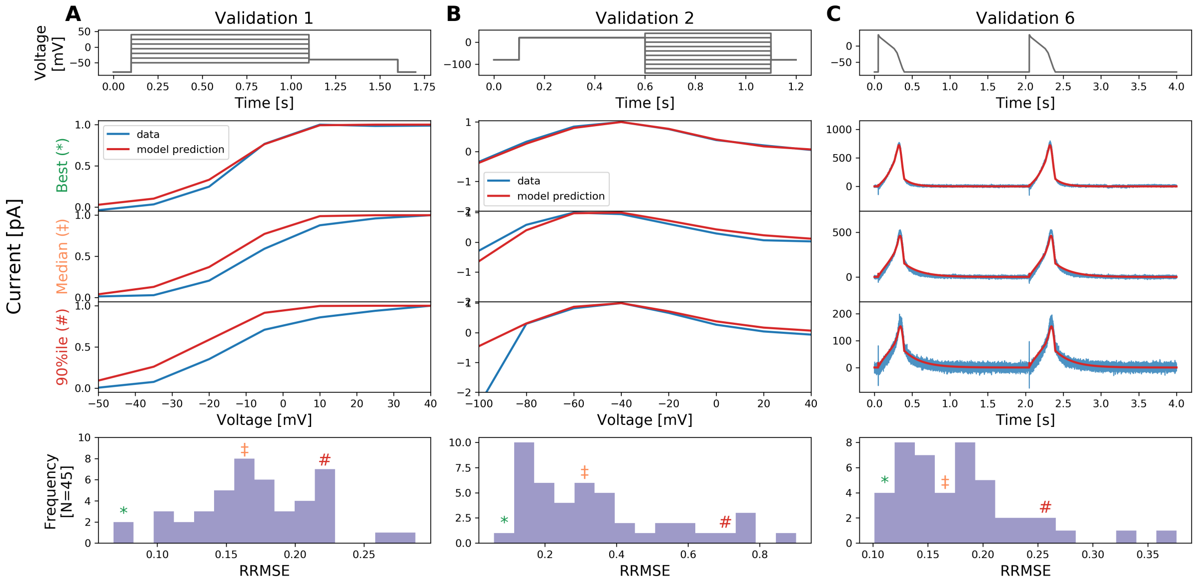

Figure S16. The relative root mean square error (RRMSE, given by Eq. 25 in the main text) histograms for all cells and for validation protocols 1, 2, and 6, at 37 °C. Markers indicate the best (∗), median (‡) and 90th percentile (#) RRMSE values. For each protocol, the raw traces with the best, median and 90th percentile RRMSE values, for both the model (red) and data (blue) are shown, with the voltage clamp above. Note that the currents are shown on different scales, to reveal the details of the traces.

S12 Automated quality control

Here we present a detailed selection results of our quality control which does not require any manual intervention. The full details of our automated quality control criteria are summarised in Lei et al. 2019[1] Table 1. A well must pass all the listed criteria in order to be selected.

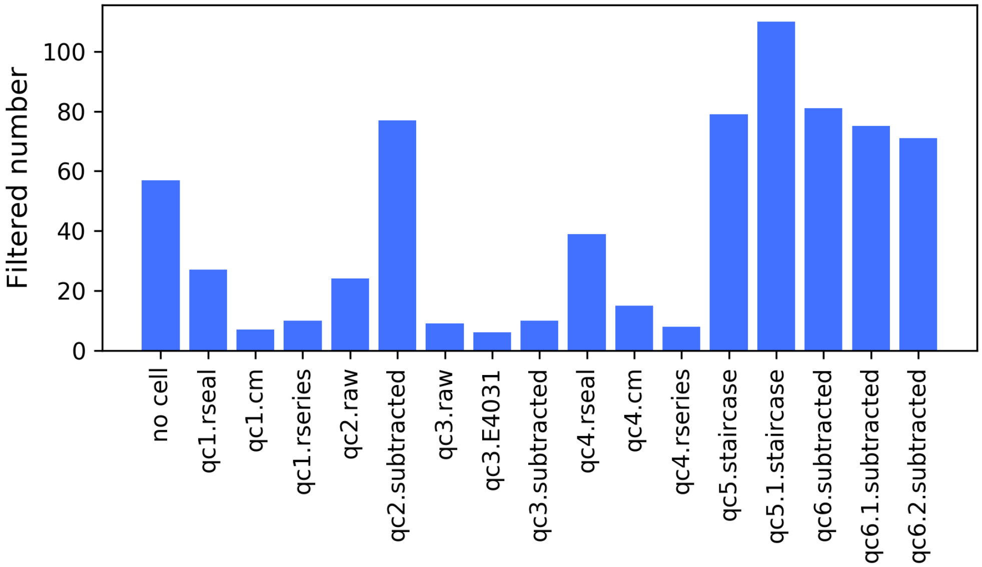

Figure S17. Selection results of each criterion from the automated quality control, showing the number of wells filtered out by each quality control criterion as bar chart, at 27 °C.

Figures S17–S20 show the results of each criterion in the automated quality control: the number of wells removed by each quality control criterion for the different temperature experiments. A similar Figure for 25 °C is shown in the partner paper Lei et al. 2019[1] (Supplement Figure S3). Figure S20 shows that for 37 °C more than 150 wells failed (almost half of 384 wells) because no cell was captured. QC4s compare the change of R_{seal}, C_{m}, and R_{series} values before and after drug addition. The lower success rate at 37 °C, compared to 25 °C (shown in the supplement of Lei et al. 2019[1]), was therefore mostly due to the cell capturing step in the high-throughput machine, before we started our recordings.

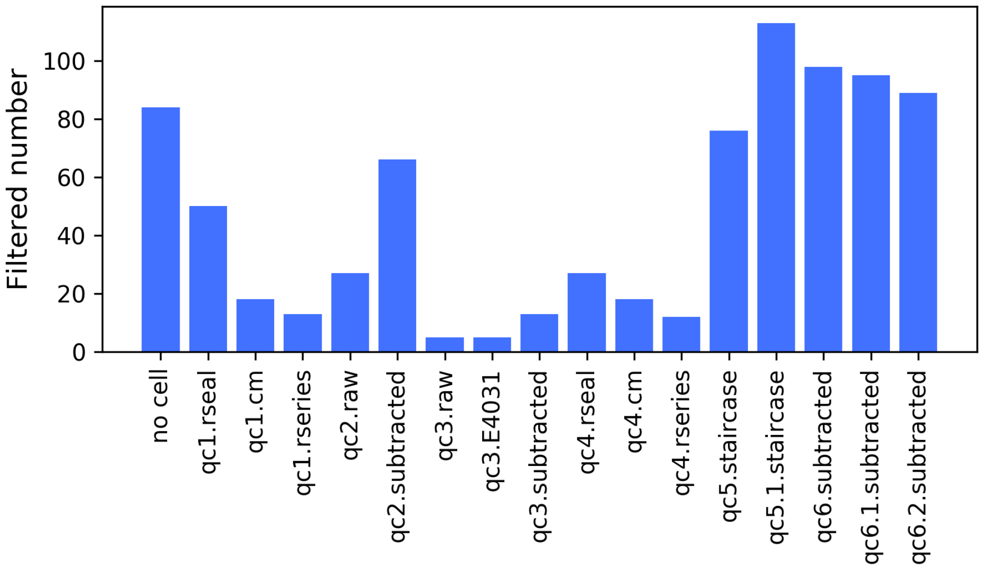

Figure S18. Selection results of each criterion from the automated quality control, showing the number of wells filtered out by each quality control criterion as bar chart, at 30 °C.

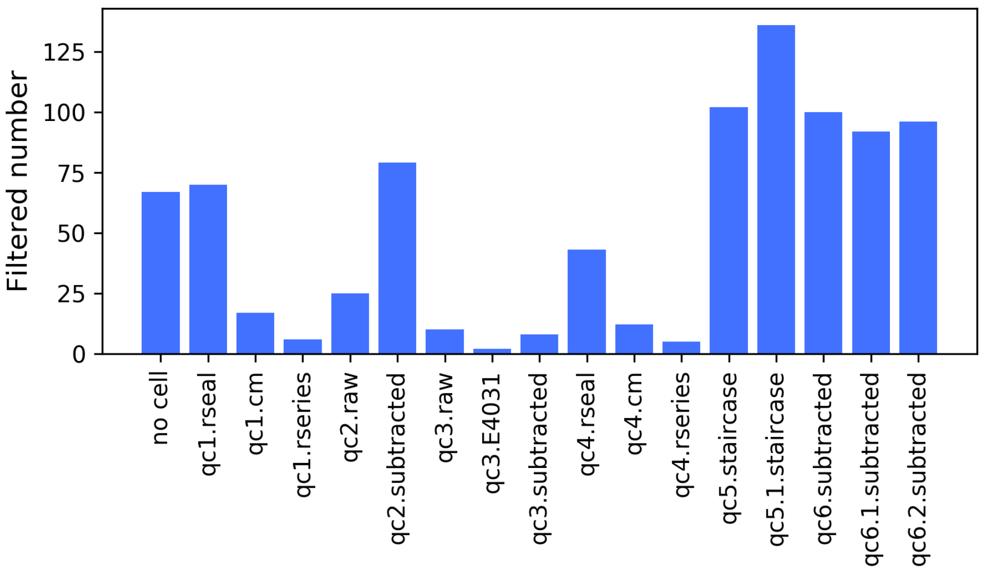

Figure S19. Selection results of each criterion from the automated quality control, showing the number of wells filtered out by each quality control criterion as bar chart, at 33 °C.

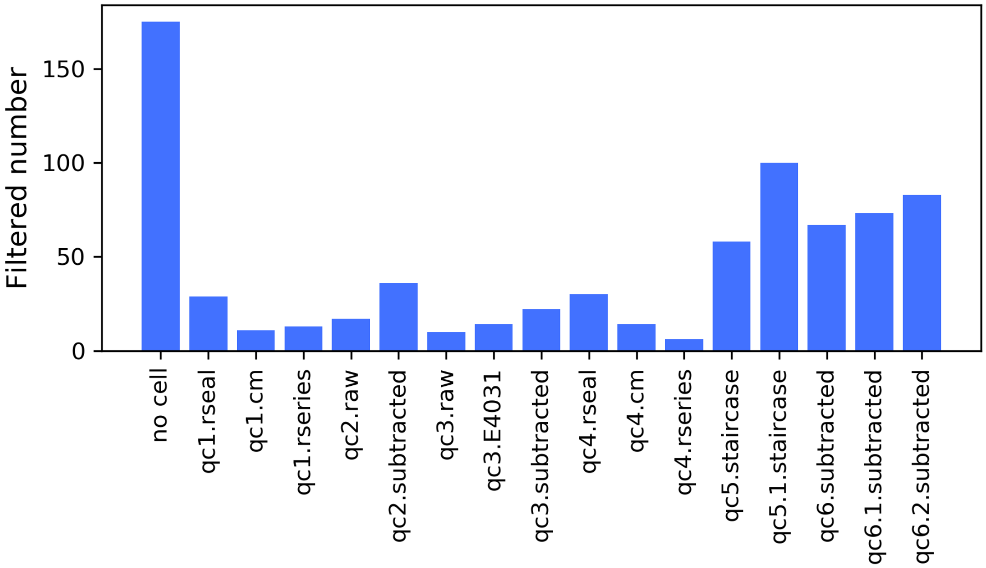

Figure S20. Selection results of each criterion from the automated quality control, showing the number of wells filtered out by each quality control criterion as bar chart, at 37 °C.

S13 A comparison with literature I_{Kr} models

Figure S21 shows a comparison of the model given by the mean of the posterior for μ at 37 °C (Table S2) with existing I_{Kr} models from within action potential models by using the Cardiac Electrophysiology Web Lab[5,6]. Interestingly, the new model shows a striking concordance for predicted current under action potential clamps with the Markov model by Fink et al.[7].

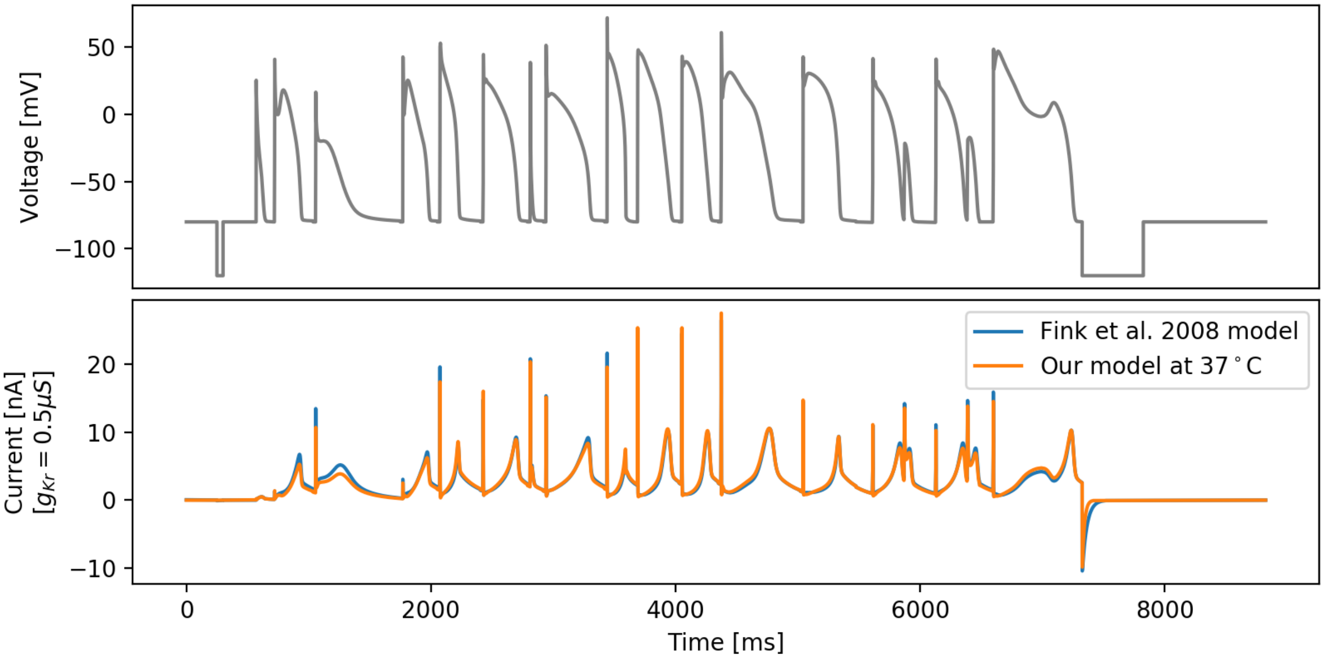

Figure S21. A comparison of predictions of Fink et al.[7] I_{Kr} model and our mean model using the posterior for μ at 37 °C, under a series of action potential clamps. Both models have the same fixed $g_{Kr} = 0.5\,\mu S$ to compare the differences in kinetics.

References

1. Lei, C. L. *et al.* Rapid characterisation of hERG potassium channel kinetics I: using an automated high-throughput system. *Biophys. J.* **this issue** (2019).
2. Vandenberg, J. I. *et al.* Temperature dependence of human ether-a-go-go-related gene K+ currents. *Am. J. Physiol. Cell Physiol.* **291**, C165–75 (2006). DOI 10.1152/ajpcell.00596.2005.
3. Zhou, Z. *et al.* Properties of HERG channels stably expressed in HEK 293 cells studied at physiological temperature. *Biophys. J.* **74**, 230–241 (1998).
4. Ku, H. H. Notes on the use of propagation of error formulas. *J. Res. Natl. Bureau Standards* **70** (1966).
5. Cooper, J., Scharm, M. & Mirams, G. R. The cardiac electrophysiology web lab. *Biophys. journal* **110**, 292–300 (2016).
6. Daly, A. C. *et al.* Reproducible model development in the cardiac electrophysiology web lab. *Prog. biophysics molecular biology* **139**, 3–14 (2018).
7. Fink, M., Noble, D., Virag, L., Varro, A. & Giles, W. R. Contributions of HERG K+ current to repolarization of the human ventricular action potential. *Prog. Biophys. & Mol. Biol.* **96**, 357–76 (2008). DOI 10.1016/j.pbiomolbio.2007.07.011.
